# Revisiting insulin resistance in human cancer cachexia – a systematic review and meta-analysis

**DOI:** 10.2340/1651-226X.2025.44280

**Published:** 2025-12-09

**Authors:** Jonas Sørensen, Anna Hammershøi, Joan Miquel Màrmol, Louise Lang Lehrskov, Ole Nørgaard, Lykke Sylow

**Affiliations:** a Department of Biomedical Sciences, Faculty of Health and Medical Sciences, University of Copenhagen, Copenhagen, Denmark; b Department of Nutrition, Exercise, and Sports, Faculty of Science, University of Copenhagen, Copenhagen, Denmark; c Center for Physical Activity Research (CFAS), Centre for Cancer and Organ Diseases, Copenhagen University Hospital – Rigshospitalet, Copenhagen, Denmark; d Department of Oncology, Copenhagen University Hospital – Herlev and Gentofte, Herlev, Denmark; e Department of Education, Danish Diabetes Knowledge Center, Copenhagen University Hospital – Steno Diabetes Center Copenhagen, Herlev, Denmark

**Keywords:** Cancer, cachexia, insulin resistance, metabolism, weight loss

## Abstract

**Background:**

In patients with cancer, unintentional weight loss and cancer-associated cachexia (CAC) reduce overall survival and impair the quality of life. Because of insulin’s anabolic effects, insulin resistance could contribute to CAC progression. However, the role of insulin resistance in CAC remains unclear, and this study aimed to investigate the association between insulin resistance and CAC. Addressing this knowledge gap may help identify treatable targets to improve patient outcomes.

**Methods:**

We performed a systematic review and meta-analysis. By including studies reporting both fasting levels of circulating insulin and glucose in patients with cancer and CAC according to the internationally accepted CAC definition, we calculated the HOMA-IR (Homeostatic Model Assessment for Insulin Resistance) index to estimate the level of insulin resistance (defined as HOMA-IR above 2.0) in patients with CAC. A subgroup analysis was conducted from studies reporting a HOMA-IR index both from a group of patients with CAC and a group without CAC (nonCAC).

**Results:**

Seventeen studies were included, with a total of 197 patients. The mean HOMA-IR of all studies was 1.84 (95% confidence interval [CI]: 1.77–1.91). Twelve studies found HOMA-IR below 2.0. Five of the 17 studies also reported HOMA-IR from a group of patients with cancer without CAC. We observed a mean difference of −0.42 (95% CI: −2.24 to 1.40) in favour of a lower HOMA-IR in patients with CAC compared to nonCAC, and thus no statistically significant difference between the groups.

**Interpretation:**

This systematic review suggests no clear association between insulin resistance and CAC. However, the limited sample sizes and study heterogeneity highlight the need for larger, longitudinal investigations.

## Introduction

Cancer-associated weight loss – termed cancer-associated cachexia (CAC) – lowers tolerance to anticancer treatment, reduces quality of life and impairs survival [1–3]. CAC is estimated to directly contribute to one in five cancer-related deaths [4], yet no U.S. Food and Drug Administration (FDA) or the European Medicines Agency (EMA)-approved drug to treat CAC is available. The underlying metabolic disturbance of CAC is not fully understood but involves the rapid depletion of both adipose tissue and skeletal muscle mass [5–7]. Insulin is a major inhibitor of adipose tissue lipolysis and controls muscle proteolysis and therefore could play a role in tissue wasting processes. While certain rodent studies indicate that insulin resistance contributes to CAC [8, 9], human data remain scarce, and the potential association between insulin resistance and CAC requires further investigation.

We recently established that patients with various cancers were markedly insulin-resistant [10]. Insulin resistance is a primary defect in type 2 diabetes, and accordingly, patients with cancer have an increased risk of new-onset type 2 diabetes after their cancer diagnosis [11, 12].

The development of CAC could be related to insulin resistance, as preexisting diabetes is related to a greater weight loss in patients with colorectal and pancreatic cancer compared to patients without diabetes [13]. However, that study did not directly assess insulin resistance and thus could not address whether there was an association between insulin resistance and CAC. Another study, which documented insulin resistance in patients with cancer using the gold standard hyperinsulinaemic euglycaemic clamp method, did not include information on weight loss [10], making it difficult to conclude on the association between insulin resistance and CAC. Molecularly, insulin’s effect on muscle protein synthesis is reduced in lung cancer patients with CAC compared to healthy controls, although no nonCAC cancer control patients were included [14]. Other clinical studies have aimed to untangle the role of insulin resistance in CAC using fasting insulin as a surrogate measure, but the findings are inconclusive and inconsistent [15, 16]. It therefore remains an unresolved question whether the documented association between cancer and insulin resistance [10] is related to the development of CAC. Such effort is challenging because the direct measurement of insulin sensitivity in humans requires a hyperinsulinaemic euglycaemic clamp that is invasive and time-consuming. Nonetheless, insulin resistance leads to elevated circulating insulin levels with, or without, an increase in blood glucose levels. These changes can be assessed using the Homeostatic Model Assessment of Insulin Resistance (HOMA-IR). Although HOMA-IR primarily estimates hepatic insulin resistance [17], it serves as a surrogate measure of whole-body insulin resistance [18] and demonstrates correlation with the hyperinsulinaemic–euglycaemic clamp [19]. Lower HOMA-IR values reflect lower blood glucose levels in relation to circulating insulin, indicating enhanced insulin sensitivity, whereas higher values suggest increased insulin resistance.

Altogether, it remains uncertain whether the documented association between cancer and insulin resistance is connected to the development of CAC. This study aimed to investigate whether insulin resistance was associated with CAC, potentially being a driver hereof. We hypothesised that higher insulin resistance is associated with CAC and conducted a systematic review of studies measuring fasting glucose and insulin to calculate HOMA-IR.

## Patients/material and methods

This systematic review was guided by the Preferred Items for Systematic Review and Meta-Analyses (PRISMA) statement [20] and PRISMA Checklist (Table S1). A review protocol was published before data extraction and can be accessed on https://www.researchregistry.com (#1869).

### Search strategy

The databases MEDLINE (via Ovid), Embase (via Ovid) and Cochrane Central Register of Controlled Trials (CENTRAL) were searched on 4 June 2024. The search was developed around three concepts – (1) cancer in humans, (2) cachexia and (3) insulin resistance expressed by the HOMA-IR index – using a combination of subject terms from the available controlled vocabularies (Medical Subject Headings and Emtree) as well as free-text terms. No restrictions were applied to publication type or language. The final search string was constructed for MEDLINE and subsequently translated to Embase and CENTRAL (Table S2 A-C) by an information specialist (O.N.). In addition, the reference lists of the included studies (backward citation) as well as studies citing the included studies (forward citation) were screened using the online tool Citation Chaser [21].

### Eligibility criteria

Studies were included based on the following criteria: (1) patients with cancer aged 18 years or older; and (2) studies that report data on weight loss and sarcopenia, sufficient for the diagnosis of CAC. The diagnostic criterion for CAC was more than 5% loss of stable body weight over the past 6 months OR a BMI less than 20 kg/m² and ongoing weight loss of more than 2% OR sarcopenia and ongoing weight loss of more than 2% without entering the refractory stage [6]. Sarcopenia is here defined as L3 CT-derived skeletal muscle index (SMI) (cm^2^/m^2^) < 43/41 for normal or underweight men/women and < 53/41 for overweight and obese men/women [3]; and (3) studies that report data on fasting blood glucose and plasma/serum insulin levels, including glucose and plasma/serum insulin levels obtained during an OGTT. Studies were excluded based on the following criteria: (1) studies only on non-cancer patients; (2) studies only on patients with haematologic malignancies; (3) studies without specific weight loss data; (4) studies without specific fasting glucose and insulin data; (5) studies with patients with documented diabetes, reported use of anti-diabetic medication or daily use of steroids or no specific information on diabetes or use of antidiabetic drugs; (6) studies including cancer survivors or studies that did not specify the cancer status of the patients; (7) studies reusing data from previously included studies, thus reanalysing the same patients; and (8) studies on animals.

### Study selection

To select studies that both included data on CAC and fasting blood glucose and insulin, three authors (J.S., A.H. and L.L.L.) independently double-screened the titles and abstracts of all identified records for eligibility. Full-text reports of the remaining studies were double-screened by the same three authors. Discrepancies were resolved through discussions and consensus. The screening process was conducted in EPPI-Reviewer Web [22].

### Data extraction

The following data were extracted from each study and are summarised in Table 1: first author (reference), year of publication, population country, target population, number of eligible patients in the study, population sex distribution, population age, weight loss %, fasting glucose, fasting insulin and calculated HOMA-IR.

**Table 1:** Characteristics of the included studies.

First author	Year	Country	Cancer site	Patient, n (F/M)	Patient, age mean (SD)	Weight loss (%)	Glucose mean, mmol/L (SD)	Insulin mean, mU/L (SD)	HOMA-IR
Bennegård K et al. [1]	1982	Sweden	Various	19 (NS)	59 (± 2)	19	5.26 (± 0.33)	7 (± 1)	**1.64**
Bennegård K et al. [2]	1983	Sweden	Various	8 (NS)	54 (± 6)	18	5.02 (± 0.2)	7 (± 1)	**1.56**
Burt ME et al.	1983	US	Esophageal	6 (1/5)	65 (± 3)	19.9	3.45 (± 0.11)	5.3 (± 1.3)	**0.81**
Eden E et al.	1984	Sweden	Various	8 (NS)	55 (± 5)	15	4.92 (± 0.2)	7 (± 1)	**1.53**
Holroyde c et al.	1984	US	Colorectal	12 (NS)	68 (NS)	10	5 (± 0.12)	14.9 (± 2)	**3.31**
Heber D et al.	1985	US	Lung	38 (NS)	59 (± 2)	16	5.16 (± 0.1)	16 (± 2)	**3.67**
Bennegård K et al. [3]	1986	Sweden	Various	5 (NS)	60 (± 2)	18	4 (± 0.61)	5 (± 2)	**0.89**
Selberg o et al.	1990	UK	Various	4 (NS)	56 (NS)	10	4.86 (± 1.21)	4.9 (± 1.22)	**1.06**
Cersosimo E et al.	1991	US	Gastrointestinal	5 (2/3)	52 (± 5)	15	5.44 (± 0.16)	9 (± 2)	**2.18**
Heslin M et al.	1992	US	Various	8 (3/5)	55 (± 2)	18	5 (± 0.16)	8 (± 1)	**1.78**
McCall J et al.	1992	New Zealand	Gastrointestinal	3 (NS)	(NS)	14.3	4.73 (± 1.18)	12.63 (± 3.1)	**2.65**
Pisters P et al.	1992	US	Gastrointestinal	6 (NS)	56 (NS)	17.5	5.17 (± 0.16)	11 (± 3)	**2.55**
Rofe A et al.	1994	Astralia	Various	35 (NS)	(NS)	13	5.11 (± 0.1)	7.8 (± 0.2)	**1.77**
Yoshikawa T et al.	1994	Japan	Various	5 (NS)	(NS)	8.6	4.77 (± 0.06)	8.3 (± 3.2)	**1.78**
Barber MD et al.	2000	UK	Pancreatic	16 (NS)	63 (± 4)	17.7	5.5 (± 0.81)	3.3 (± 2.22)	**0.81**
Leij-Halfwerk S et al.	2000	The Netherlands	Lung	9 (NS)	67 (NS)	12	5.8 (± 0.3)	5.85 (± 2)	**1.51**
Agustsson T et al.	2007	Sweden	Gastrointestinal	15 (3/12)	65 (± 5)	20	6.4 (± 1.6)	6.7 (± 3.3)	**1.90**

F/M: female/male; SD: standard deviation; NS: not stated; HOMA-IR: Homeostatic Model Assessment of Insulin Resistance; %: percentage; mmol: millimole; mU: milliunits; L: litre.

### Quality assessment

Two review authors (J.S., A.H.) independently assessed the methodological quality of the included studies using appropriate checklists provided by Joanna Briggs Institute (JBI) critical appraisal tools [23] (Supporting Information S3–19). All eligible studies were included regardless of methodological quality, but their limitations were considered in the interpretation of results [24].

### Data analysis

The research was synthesised in a systematic manner. The data extracted from the reviewed studies were based on (1) insulin sensitivity (IR) reported as HOMA-IR = (insulin × glucose)/22.5 for the glucose concentration in mmol/L, or HOMA-IR = (insulin × glucose)/405 for glycaemia in mg/dL; in both cases, the insulin is in mU/L or uU/ml; and (2) weight loss% (WL%) is reported in the most detailed version possible.

Insulin resistance has been defined as a HOMA-IR cut-off of 2.0, representing 25% of the population with the highest fasting insulin concentrations [18, 25]. This cut-off was applied, and the prevalence of insulin resistance in the CAC patients was calculated.

Following meta-analytic methods, we calculated a pooled mean HOMA-IR, and through its standard deviation (SD), a 95% confidence interval (CI) was calculated using the inverse variance method and a random-effects model. Heterogeneity detected by the I square (I^2^) test, significant heterogeneity was defined as I^2^ > 50% with a *P* < 0.05. Subgroup analysis was performed to discover the source of heterogeneity. We used the Cohen statistic and investigated the influence of each study on pooled effect size.

## Results

### Eligible studies

**Figure 1:**
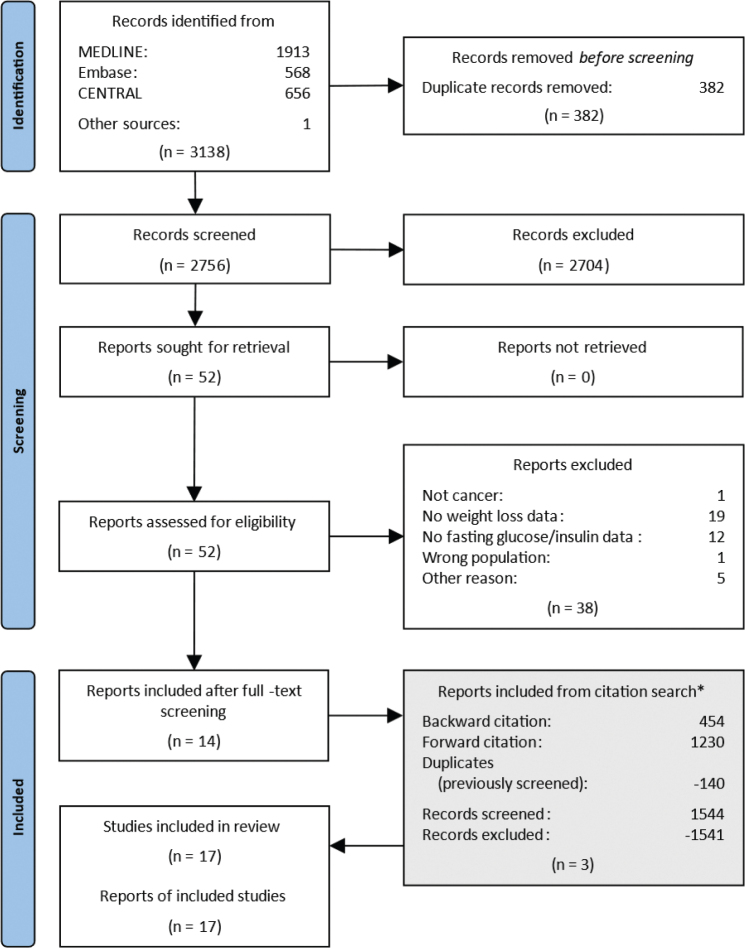
Flow diagram of study selection. *The reports identified from the citation search are located at the bottom to maintain a chronological flow in the diagram. However, they have gone through the same process of deduplication, title and abstract screening, and full text screening.

Our search yielded 3,138 records. After removal of 382 duplicates, 2,756 records remained for title and abstract screening (Figure 1). Following this screening, 52 records remained for full-text screening. It was possible to retrieve all the full-text articles. Thirty-eight records were excluded for not meeting our eligibility criteria (see reasons for exclusion in Table S20). Additionally, three reports were identified through citation searching. In total, 17 studies including 197 patients with cancer and CAC reported measures on fasting glucose and insulin and were included in the analysis. Studies were published from 1982 to 2007.

For all eligible studies, direct personal contact was attempted by contacting the authors via email. In two cases, a personal reply from the 1st author resulted in individual values from three patients [26] and four patients [16], respectively. No other authors were able to share individual values on fasting glucose and insulin. For that reason, individual values for each patient in this review were not available, and therefore one mean HOMA-IR value was used per study. Studies reporting blood glucose and insulin values from arterial blood levels were accepted [27, 28]; because of the fasting and resting condition of the patients, the arterial-venous difference was assumed to be zero [29]. In two studies, it was possible from a manuscript figure to determine the mean values of glucose [30] and insulin [31], respectively, by using the tool PlotDigitizer [32]. In these two studies, the SD was estimated as one-quarter of the mean. Where glucose and insulin levels were reported with SE (standard error), SD was calculated by multiplying SE by the square root of the sample size. One study reported glucose and insulin in median/IQR [33] – here we assumed normal distribution (mean = median) and estimated SD = IQR/1.35. SD for the calculated HOMA-IR could be calculated as the square root of the HOMA-IR variance, and 95% CI = mean ± 1.96 × SD/√*n*.

### Study characteristics

Table 1 summarises the main characteristics of the included studies. Of the 17 studies included with a total of 197 patients with CAC, the HOMA-IR values ranged from 0.81 to 3.67. All studies except three [26, 34, 35] reported the mean age, ranging from 52 to 68 years. Five studies reported on the distribution between females and males in patients with CAC [16, 36-39]. The sample size in each study ranged from three to 38 patients. One study [39] included two separate groups of patients with CAC – one with and one without secondary nutrition impact symptoms, for example, anorexia. Without contradicting the inclusion criteria, these two CAC groups were merged into one.

Two studies included solely patients with lung cancer [31, 40]; seven studies included patients with gastrointestinal cancers, oesophageal cancer [36], colorectal cancer [30], upper gastrointestinal cancer [41], gastrointestinal cancers not otherwise specified [26, 37, 39], pancreatic cancer [33]; and eight studies included patients with various cancer diagnoses [15, 16, 27, 28, 34, 35, 38, 42]. Eight studies reported that all included patients were untreated for their cancer [15, 27, 28, 33, 37, 39, 41, 42]. Two studies reported that a fraction of the patients included were pretreated for the cancer [31, 38]. One study reported that included patients did not have any anticancer treatment three months prior to the study [26]. One study reported that patients were included immediately prior to the next line of treatment [34]. One study stated that all patients were pretreated for the cancer [30]. Eight studies reported that some or all patients had advanced disease [26–28, 30, 31, 34, 35, 42]. Two studies reported that patients included only had local disease [36, 41]. None of the studies reported clearly about ethnicity in the patients included. All but one study [35] were performed in the US, Europe, Australia or New Zealand.

### Meta-analyses

**Figure 2:**
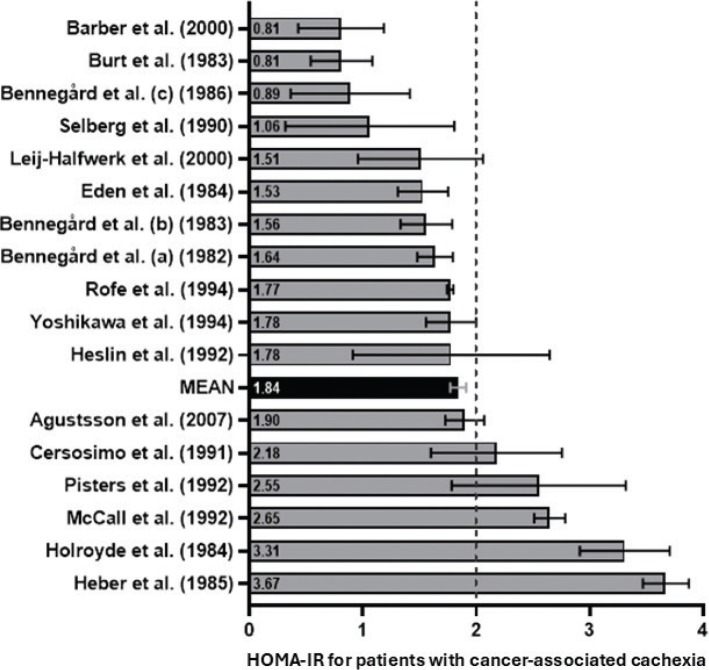
Homeostatic Model Assessment of Insulin Resistance for studies on patients with cancer-associated cachexia. Studies are lined up according to the individual calculated HOMA-IR values, including 95% confidence interval. A vertical dotted line represents the HOMA-IR cut-off value of 2.0. A mean HOMA-IR for all 17 studies including a 95% confidence interval is shown in black. HOMA-IR: Homeostatic Model Assessment of Insulin Resistance.

The mean HOMA-IR of the 17 studies with patients with CAC was 1.84 (95% CI: 1.77–1.91). Figure 2 shows a forest plot with individual HOMA-IR and 95% CI depicted along a vertical line representing the cut-off value of 2.0 (see Data analysis section). In five studies, the patients had a mean HOMA-IR higher than the defined cut-off of 2.0 [26, 30, 37, 40, 41]. In 12 studies, the patients had a mean HOMA-IR below 2.0; four below 1.1 [15, 16, 33, 36]; and in eight of the studies, the mean HOMA-IR was between 1.5 and 2.0 [27, 28, 31, 34, 35, 38, 39, 42]. Thus, overall, we did not find indications of insulin resistance in 12 of the 17 included studies, challenging the notion that insulin resistance is associated with CAC.

**Figure 3:**
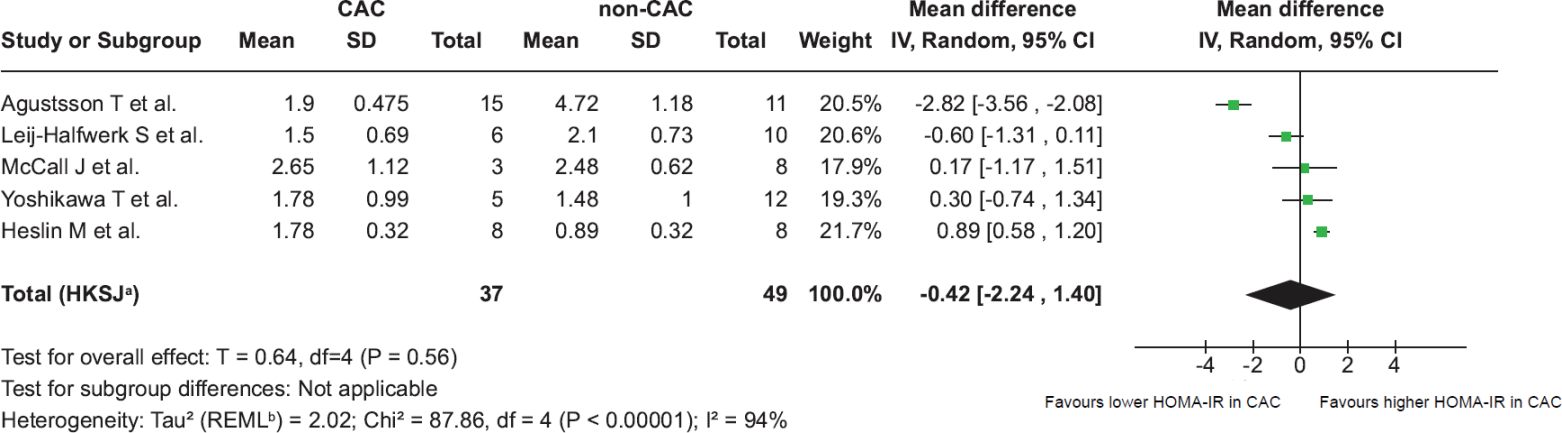
Forest plot comparing Homeostatic Model Assessment of Insulin Resistance stratifying patients with and without cancer-associated cachexia. Forest plot comparing HOMA-IR in the five studies (mean) stratifying patients with CAC and nonCAC. CAC: patients with cancer-associated cachexia; nonCAC: patients without cancer-associated cachexia; SD: standard deviation; CI: confidence interval; IV: inverse variance; HOMA-IR: Homeostatic Model Assessment of Insulin Resistance. A Random effect model is used to account for both within study and between-study variability. a 95% CI calculated by Hartung-Knapp-Sidik-Jonkman method. b Tau2 calculated by Restricted Maximum-Likelihood method.

Next, we aimed to compare HOMA-IR between patients with cancer and CAC and those with nonCAC. However, not all studies provided data allowing comparisons with a cancer population without CAC, limiting a direct assessment of the difference in HOMA-IR between the two groups. Nevertheless, we identified five out of 17 included studies that reported HOMA-IR values for a nonCAC group, defined as having no weight loss or weight loss of < 5% in the last 6 months [6] (Table 2). A secondary analysis of these studies was therefore possible [26, 31, 35, 38, 39] and we analysed HOMA-IR from five studies including 37 patients with CAC and 49 patients with nonCAC (Figure 3). Here we observed a mean difference of −0.42 (95% CI: −2.24 to 1.40). In the CAC and nonCAC groups, the HOMA-IR index ranged from 1.51 to 2.65 and 0.89 to 4.72, respectively (Figure 3). Thus, this separate meta-analysis showed a mean difference of −0.42 in favour of a lower HOMA-IR in patients with CAC compared to patients with nonCAC (Figure 3). However, the 95% CI crosses zero, and our analysis cannot demonstrate a significant association between CAC and insulin resistance.

**Table 2:** Characteristics of the non-cancer associated cachexia groups, used for subgroup analysis.

First author	Year	Cancer site	Patient, n (F/M)	Glucose mean, mmol/L (SD)	Insulin mean, mU/L (SD)	HOMA-IR
Heslin M et al.	1992	Various	8 (NS)	5 (±0.1)	4 (±1)	**0.89**
McCall J et al.	1992	Gastrointestinal	8 (NS)	4.2 (±0.11)	13.22 (±8.87)	**2.48**
Yoshikawa T et al.	1994	Various	12 (NS)	5.21 (±0.05)	6.4 (±3.1)	**1.48**
Leij-Halfwerk S et al.	2000	Lung	10 (NS)	5.3 (±0.2)	8.92 (±2.23)	**2.10**
Agustsson T et al.	2007	Gastrointestinal	11 (NS)	6.6 (±1.8)	16.1 (±3.1)	**4.72**

F/M: female/male; SD: standard deviation; NS: not stated; HOMA-IR: Homeostatic Model Assessment of Insulin Resistance.

### Quality assessment

The results of the methodological quality assessment of the studies included using the JBI Critical Appraisal Tools (Supplemental materials S3–19). We observed significant heterogeneity between the studies with *I*^2^ = 94% with a *P* < 0.05. A funnel plot assessing publication bias was opted out because of the low sample size of five studies in the subgroup analysis.

## Discussion and conclusion

To our knowledge, this is the first systematic review examining the association between CAC and insulin resistance, assessed by HOMA-IR. A total of 17 studies comprising 197 patients with cancer and CAC were included. Our results indicate that most patients with cancer and CAC are within the normal insulin sensitivity reference range.

We showed that most patients with cancer and CAC fall within the normal insulin sensitivity range, suggesting that insulin resistance is not a primary driver of CAC. These results appear to contradict previous evidence and the prevailing belief that insulin resistance is associated with CAC. Notably, patients with cancer in general exhibit insulin resistance as documented in a recent meta-analysis [10] and accordingly have a higher risk of developing type 2 diabetes [11, 12]. Moreover, patients with cancer have been reported to respond to elevated glucose levels in a diabetic manner with reduced glucose disposal curves [43, 44]. While documenting insulin resistance and dysregulated glucose metabolism, none of those studies stratified patients with CAC, which was done in our study. These present results challenge the prevailing notion that insulin resistance serves as a driving factor for CAC.

In contrast to the overall normal fasting blood glucose in all but two studies one reporting hypoglycaemia of 3.45 mmol/L [36] and one reporting hyperglycaemia of 5.8 mmol/L [31]), we observed great variation in fasting insulin levels between studies. There is no international standard normal range for fasting insulinaemia in a non-diabetic population, but a normo-insulinaemic range from 2 to 15 mU/L is accepted [25, 45]. Five studies were found in the low-normal end with insulin levels < 6 mU/L [15, 16, 31, 33, 36]. Five studies with normal to high insulin levels (≥ 9 mU/L) were identified, all of which also had a calculated HOMA-IR > 2.0 [26, 30, 37, 40, 41].

While most studies reported that patients with cancer and CAC fall within the normal insulin sensitivity range, we did identify five studies with a mean HOMA-IR > 2.0 [26, 30, 37, 40, 41], indicative of insulin resistance (Figure 2). In all five studies, the high HOMA-IR was driven by normal-high insulinaemia and not hyperglycaemia. Hyperinsulinaemia in the presence of euglycaemia is a hallmark of prediabetes that is primarily because of peripheral insulin resistance of the muscle [17]. Unfortunately, peripheral insulin resistance was not determined in this study but is highly common in the general cancer patient population [10]. No common features in relation to cancer types, treatment status, degree of weight loss, age or sex distribution from the five studies with HOMA-IR > 2.0 can be extracted besides all being conducted in the US or New Zealand. Thus, no confounding factors were identified that could explain the association or lack thereof between CAC and insulin resistance.

The findings from the meta-analysis did not have the statistical power to show a significant difference in HOMA-IR between CAC and nonCAC patients. Other studies have shown that in response to a glucose challenge, the insulin response is lower in patients with cancer and CAC compared to nonCAC [46, 47], indicative of increased insulin sensitivity in patients with CAC. Accordingly, underweight patients with cancer exhibited greater insulin sensitivity compared to their normal-weight counterparts, as estimated in response to intravenous glucose infusion [48].

Supported by our main finding, that most patients with cancer and CAC are within the normal insulin sensitivity reference range, the mentioned studies [46–48] could suggest that while patients with cancer generally exhibit insulin resistance and metabolic dysregulation, patients with CAC appear to either preserve insulin sensitivity or counteract the insulin-resistance-inducing effects of cancer to some extent.

The underlying biological mechanisms were not identified in the present study but could include several factors.

Firstly, lower insulin resistance in patients with CAC, compared to the general cancer population, may be attributed to malnutrition. Both low body weight and reduced food intake are generally associated with increased insulin sensitivity and low insulin levels in humans [48] and preclinical models [49]. Consistently, five of our included studies reported fasting insulin levels within the low-normal range in patients with CAC [15, 16, 31, 33, 36]. Therefore, the well-documented cancer-associated insulin resistance in humans [10] may be partially counteracted by malnutrition and/or progressive weight loss in patients with CAC.

Secondly, increased glucose disposal into highly glycolytic tumours may contribute to overall improved whole-body glycaemic control, leading to a lower HOMA-IR. However, it remains unclear whether tumours that induce CAC exhibit higher metabolic rates and consequently utilise more glucose compared to nonCAC-inducing tumours.

Thirdly, metabolic reprogramming in tissues undergoing wasting may contribute to the observed absence of insulin resistance in patients with CAC compared to the general cancer population. Cancer and cancer-secreted factors can induce profound metabolic disruptions, as documented in numerous preclinical [9, 50-52] and clinical studies [53-55]. In the context of CAC, the activation of catabolic pathways and the disruption of energy homeostasis in adipose and muscle tissue may paradoxically enhance insulin sensitivity or induce glucose uptake via insulin-independent pathways [56]. This metabolic reprogramming, characteristic of CAC, could play a key role in mitigating insulin resistance.

This study has several limitations that should be considered when interpreting the findings. The study populations are heterogeneous, with variations in treatment regimens, unreported comorbidities, unreported stages of the cancers and concurrent therapies. This introduced confounding factors and reduced the internal validity. The considerable heterogeneity between the studies indicates that the true effect size varies considerably, making it more difficult to interpret pooled results. To reduce the heterogeneity in the studied populations, we decided to include studies on patients with solid tumours and excluded studies on patients with haematological malignancies, a group of patients less well studied in relation to CAC and known to have a prevalence of CAC below 40% [57, 58]. This decision reduced the number of studies included and is therefore a limitation to our study. Additionally, the 17 studies were conducted across different institutions over a span of 25 years, with inconsistencies in insulin assays and sample handling that may have influenced HOMA-IR measurements. Furthermore, individual fasting glucose and insulin levels were unavailable, requiring reliance on mean values to calculate HOMA-IR at the group level for each study. Our meta-analysis on a subgroup of studies was underpowered and could not show a statistically significant difference in HOMA-IR between CAC and nonCAC patients. A large systematic review clearly stating the CAC status of all patients and reporting individual levels of insulin sensitivity obtained from a hyperinsulinaemic euglycaemic clamp, measuring whole-body insulin sensitivity, would be the optimal design; unfortunately, the details are not available [10]. Given these limitations and the high heterogeneity of the included studies, it is difficult to draw firm conclusions, underscoring the need for further research. We encourage future studies to reduce heterogeneity in the studied populations and to include CAC status when performing hyperinsulinaemic euglycaemic clamps on patients with cancer.

Despite these limitations, this is the first comprehensive analysis of studies measuring HOMA-IR in patients with cancer and CAC, indicating that insulin resistance is less pronounced in CAC compared to nonCAC cancer populations.

Further research, particularly longitudinal human studies directly assessing insulin sensitivity, is essential to uncover the biological mechanisms. As our understanding of CAC-associated metabolic alterations advances, exciting discoveries may change our approach to cancer metabolism and its complications.

## Conclusion

The role of insulin resistance as a driver of cachexia in humans has remained unclear. Our findings suggest no clear association between insulin resistance and CAC. The limited sample sizes and study heterogeneity highlight the need for larger, longitudinal investigations, which are needed to uncover molecular disturbances during treatment and identify potential therapeutic targets. Such investigations may reshape our understanding of the complex relationship between insulin resistance and CAC.

## Supplementary Material



## Data Availability

The data that support the findings of this study are available from the corresponding author, J.S., upon reasonable request.
