# Supplementary material for: Revisiting insulin resistance in human cancer cachexia – a systematic review and meta-analysis
Source: Acta Oncol. 2025 Dec 9;64:44280. doi: 10.2340/1651-226X.2025.44280 (PMC12701502; doi:10.2340/1651-226X.2025.44280)
Supplement: Supplementary file 1 [file AO-64-44280-s1.pdf]

Supplementary material has been published as submitted. It has not been copyedited, or typeset by Acta Oncologica

Table S1 PRISMA checklist

Section and Topic	Item #	Checklist item	Location where item is reported
**TITLE**			
Title	1	Identify the report as a systematic review.	Title page
**ABSTRACT**			
Abstract	2	See the PRISMA 2020 for Abstracts checklist.	Abstract section
**INTRODUCTION**			
Rationale	3	Describe the rationale for the review in the context of existing knowledge.	Introduction section
Objectives	4	Provide an explicit statement of the objective(s) or question(s) the review addresses.	Introduction section
**METHODS**			
Eligibility criteria	5	Specify the inclusion and exclusion criteria for the review and how studies were grouped for the syntheses.	Methods section
Information sources	6	Specify all databases, registers, websites, organisations, reference lists and other sources searched or consulted to identify studies. Specify the date when each source was last searched or consulted.	Methods section
Search strategy	7	Present the full search strategies for all databases, registers and websites, including any filters and limits used.	Methods section
Selection process	8	Specify the methods used to decide whether a study met the inclusion criteria of the review, including how many reviewers screened each record and each report retrieved, whether they worked independently, and if applicable, details of automation tools used in the process.	'Study selection' in Methods section
Data collection process	9	Specify the methods used to collect data from reports, including how many reviewers collected data from each report, whether they worked independently, any processes for obtaining or confirming data from study investigators, and if applicable, details of automation tools used in the process.	'Data extraction' in Methods section
Data items	10a	List and define all outcomes for which data were sought. Specify whether all results that were compatible with each outcome domain in each study were sought (e.g. for all measures, time points, analyses), and if not, the methods used to decide which results to collect.	'Data analysis' in Methods section
	10b	List and define all other variables for which data were sought (e.g. participant and intervention characteristics, funding sources). Describe any assumptions made about any missing or unclear information.	Methods section
Study risk of bias assessment	11	Specify the methods used to assess risk of bias in the included studies, including details of the tool(s) used, how many reviewers assessed each study and whether they worked independently, and if applicable, details of automation tools used in the process.	'Quality assessment' in Methods section
Effect measures	12	Specify for each outcome the effect measure(s) (e.g. risk ratio, mean difference) used in the synthesis or presentation of results.	'Data analysis' in Methods section
Synthesis methods	13a	Describe the processes used to decide which studies were eligible for each synthesis (e.g. tabulating the study intervention characteristics and comparing against the planned groups for each synthesis (item #5)).	'Study selection' in Methods section
	13b	Describe any methods required to prepare the data for presentation or synthesis, such as handling of missing summary statistics, or data conversions.	'Data analysis' in Methods section + 'Eligible studies'

Supplementary material has been published as submitted. It has not been copyedited, or typeset by Acta Oncologica

Table S1 PRISMA checklist

Section and Topic	Item #	Checklist item	Location where item is reported
			in Results section
	13c	Describe any methods used to tabulate or visually display results of individual studies and syntheses.	'Data analysis' in Methods section
	13d	Describe any methods used to synthesize results and provide a rationale for the choice(s). If meta-analysis was performed, describe the model(s), method(s) to identify the presence and extent of statistical heterogeneity, and software package(s) used.	'Data analysis' in Methods section
	13e	Describe any methods used to explore possible causes of heterogeneity among study results (e.g. subgroup analysis, meta-regression).	'Data analysis' in Methods section
	13f	Describe any sensitivity analyses conducted to assess robustness of the synthesized results.	'Data analysis' in Methods section
Reporting bias assessment	14	Describe any methods used to assess risk of bias due to missing results in a synthesis (arising from reporting biases).	'Quality assessment' in Methods section
Certainty assessment	15	Describe any methods used to assess certainty (or confidence) in the body of evidence for an outcome.	'Data analysis' in Methods section
**RESULTS**			
Study selection	16a	Describe the results of the search and selection process, from the number of records identified in the search to the number of studies included in the review, ideally using a flow diagram.	'Eligible studies' in Results section
	16b	Cite studies that might appear to meet the inclusion criteria, but which were excluded, and explain why they were excluded.	Supporting Information S20
Study characteristics	17	Cite each included study and present its characteristics.	Table 1A, 'Study characteristics' in Results section
Risk of bias in studies	18	Present assessments of risk of bias for each included study.	Supporting information S3-19
Results of individual studies	19	For all outcomes, present, for each study: (a) summary statistics for each group (where appropriate) and (b) an effect estimate and its precision (e.g. confidence/credible interval), ideally using structured tables or plots.	Figure 2 and Figure 3 in Results section
Results of syntheses	20a	For each synthesis, briefly summarise the characteristics and risk of bias among contributing studies.	'Study characteristics' in Results section
	20b	Present results of all statistical syntheses conducted. If meta-analysis was done, present for each the summary estimate and its precision (e.g. confidence/credible interval) and measures of statistical heterogeneity. If comparing groups, describe the direction of the effect.	'Meta analyses and Figure 3 in Results section
	20c	Present results of all investigations of possible causes of heterogeneity among study results.	'Meta analyses' and Figure 3 in Results section

Table S1 PRISMA checklist

Section and Topic	Item #	Checklist item	Location where item is reported
	20d	Present results of all sensitivity analyses conducted to assess the robustness of the synthesized results.	Figure 3 in Results section
Reporting biases	21	Present assessments of risk of bias due to missing results (arising from reporting biases) for each synthesis assessed.	Supporting information S3-19
Certainty of evidence	22	Present assessments of certainty (or confidence) in the body of evidence for each outcome assessed.	Supporting information S3-19
**DISCUSSION**			
Discussion	23a	Provide a general interpretation of the results in the context of other evidence.	Discussion section
	23b	Discuss any limitations of the evidence included in the review.	Discussion section
	23c	Discuss any limitations of the review processes used.	Discussion section
	23d	Discuss implications of the results for practice, policy, and future research.	Conclusion section
**OTHER INFORMATION**			
Registration and protocol	24a	Provide registration information for the review, including register name and registration number, or state that the review was not registered.	Methods section
	24b	Indicate where the review protocol can be accessed, or state that a protocol was not prepared.	Methods section
	24c	Describe and explain any amendments to information provided at registration or in the protocol.	Methods section
Support	25	Describe sources of financial or non-financial support for the review, and the role of the funders or sponsors in the review.	Acknowledgments section
Competing interests	26	Declare any competing interests of review authors.	Disclosure statement
Availability of data, code and other materials	27	Report which of the following are publicly available and where they can be found: template data collection forms; data extracted from included studies; data used for all analyses; analytic code; any other materials used in the review.	Data extracted from included studies; data used for all analyses; and data that supports the findings of this study. See also Data availability statement

From: Page MJ, McKenzie JE, Bossuyt PM, Boutron I, Hoffmann TC, Mulrow CD, et al. The PRISMA 2020 statement: an updated guideline for reporting systematic reviews. BMJ 2021;372:n71. doi: 10.1136/bmj.n7

Supplementary material has been published as submitted. It has not been copyedited, or typeset by Acta Oncologica

Table S2 A-C

Table S2 A: Search in MEDLINE (Ovid)
Date of search: 4 June 2024

#	Searches	Results
1	exp Neoplasms/	3979014
2	(adenocarcinoma$ or cancer$ or carcinoma$ or choricarcinoma$ or malignan$ or metasta$ or neoplasm$ or sarcoma$ or teratoma$ or tumo?r$).ti,ab,kf,kw.	4281894
3	or/1-2	5317591
4	exp Malnutrition/	138265
5	exp Muscular Atrophy/	23541
6	exp Weight Loss/	51417
7	(cachex$ or malnourish$ or malnutri$ or (musc$ adj (atroph$ or loss)) or sarcopeni$ or underweight or wasting or (weight adj3 (loss or redu$))).ti,ab,kf,kw.	283421
8	or/4-7	413149
9	Blood Glucose/ and (fasting or toleran$).ti,ab,kf,kw.	52380
10	Glucose Clamp Technique/	5757
11	exp Insulin Resistance/	102705

Supplementary material has been published as submitted. It has not been copyedited, or typeset by Acta Oncologica

12	(((bloodglucose or glucose) adj3 (fasting or toleran$)) or (euglyc?emic adj3 clamp$) or homa-ir or (insulin adj3 (resistan$ or sensitiv$)) or ogtt or ((plasma$ or serum) adj insulin)).ti,ab,kf,kw.	216902
13	or/9-12	257298
14	and/3,8,13	2200
15	exp Animals/ not Humans/	5227826
16	14 not 15	1913

Supplementary material has been published as submitted. It has not been copyedited, or typeset by Acta Oncologica

Table S2 B: Search in Embase (Ovid)
Date of search: 4 June 2024

#	Searches	Results
1	exp neoplasms/	5768417
2	(adenocarcinoma$ or cancer$ or carcinoma$ or choricarcinoma$ or malignan$ or metasta$ or neoplasm$ or sarcoma$ or teratoma$ or tumo?r$).ti,ab,kf,kw.	5697222
3	or/1-2	7053235
4	exp malnutrition/	200458
5	exp muscular atrophy/	63682
6	exp body weight loss/	102050
7	(cachex$ or malnourish$ or malnutri$ or (musc$ adj (atroph$ or loss)) or sarcopeni$ or underweight or wasting or (weight adj3 (loss or redu$))).ti,ab,kf,kw.	424587
8	or/4-7	603132
9	exp blood glucose/ and (fasting or toleran$).ti,ab,kf,kw.	106230
10	exp glucose clamp technique/	5137
11	insulin resistance/	154091
12	(((bloodglucose or glucose) adj3 (fasting or toleran$)) or (euglyc?emic adj3 clamp$) or homa-ir or (insulin adj3 (resistan$ or sensitiv$)) or ogtt or ((plasma$ or serum) adj insulin)).ti,ab,kf,kw.	317319
13	or/9-12	366596
14	and/3,8,13	3659
15	(rat or rats or mouse or mice or swine or porcine or murine or sheep or lambs or pigs or piglets or rabbit or rabbits or cat or cats or dog or dogs or cattle or bovine or monkey or monkeys or trout or marmoset$1).ti. and animal experiment/	1254241
16	animal experiment/ not (human experiment/ or human/)	2637299
17	15 or 16	2711386
18	14 not 17	3224
19	limit 18 to exclude medline journals	568

Supplementary material has been published as submitted. It has not been copyedited, or typeset by Acta Oncologica

Supplementary material has been published as submitted. It has not been copyedited, or typeset by Acta Oncologica

Table S2 C: Search in CENTRAL (Cochrane Library)
Date of search: 4 June 2024

#	Searches (in Trials)	Results
#1	[mh Neoplasms]	124145
#2	(adenocarcinoma* OR cancer* OR carcinoma* OR choricarcinoma* OR malignan* OR metasta* OR neoplasm* OR sarcoma* OR teratoma* OR tumo?r*):ti,ab,kw	278528
#3	{OR #1-#2}	289925
#4	[mh Malnutrition]	6094
#5	[mh "Muscular Atrophy"]	1424
#6	[mh "Weight Loss"]	9011
#7	(cachex* OR malnourish* OR malnutri* OR (musc* NEXT (atroph* or loss)) OR sarcopeni* OR underweight OR wasting OR (weight NEAR/2 (loss OR redu*))):ti,ab,kw	43678
#8	{OR #4-#7}	47516
#9	[mh ^"Blood Glucose"] AND (fasting OR toleran*):ti,ab,kw	9478
#10	[mh "Glucose Clamp Technique"]	1161
#11	[mh "Insulin Resistance"]	9615
#12	(((bloodglucose OR glucose) NEAR/2 (fasting OR toleran*)) OR (euglyc?emic NEAR/2 clamp*) OR homa-ir OR (insulin NEAR/2 (resistan* OR sensitiv*)) OR ogtt OR ((plasma* OR serum) NEXT insulin)):ti,ab,kw	43105
#13	{OR #9-#12}	47102
#14	#3 AND #8 AND #13	656

Supplementary material has been published as submitted. It has not been copyedited, or typeset by Acta Oncologica

S3 JBI checklist

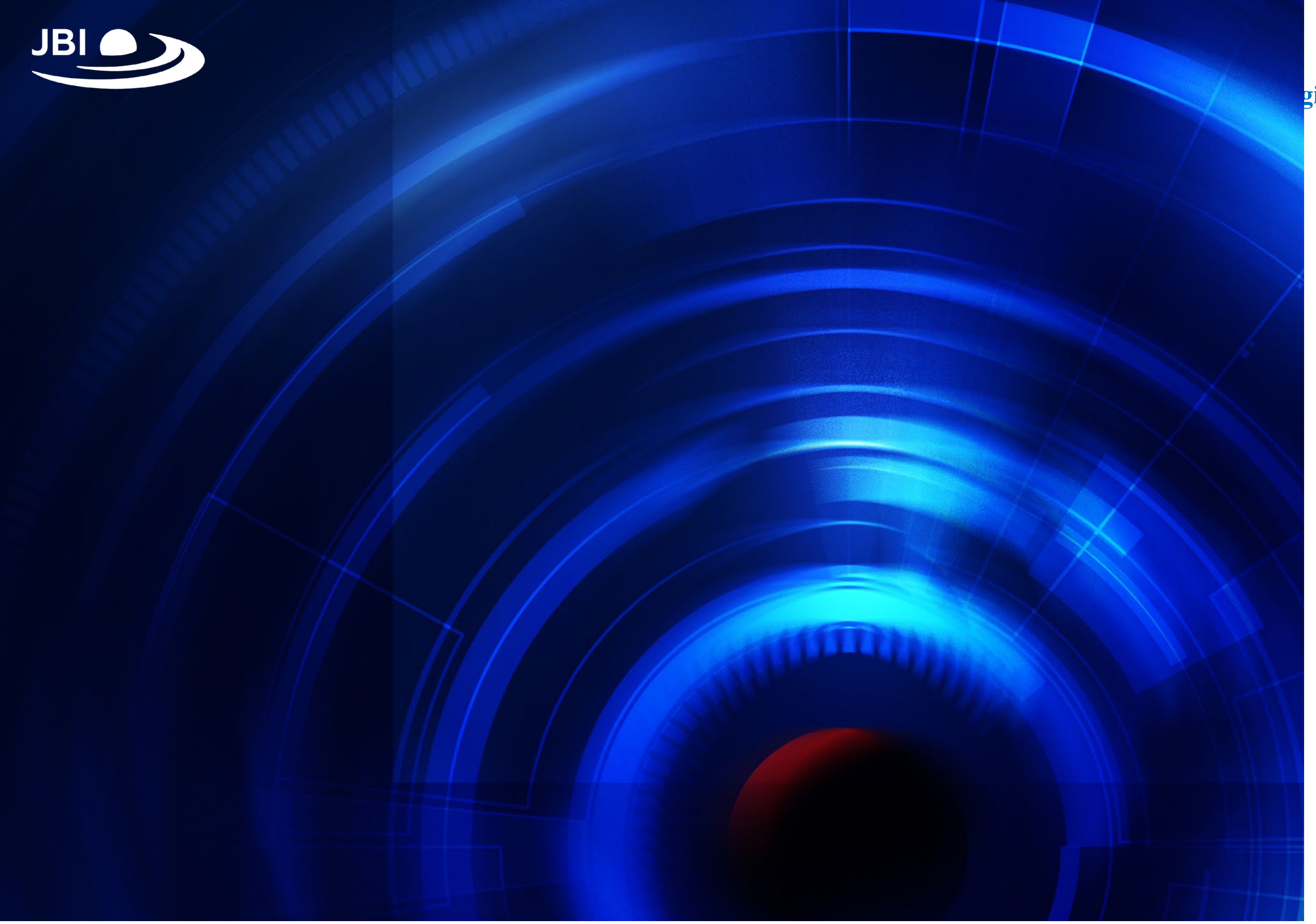

gica

JBI CRITICAL APPRAISAL TOOL

Supplementary material has been published as submitted. It has not been copyedited, or typeset by Acta Oncologica

JBI CHECKLIST FOR QUASI-EXPERIMENTAL STUDIES

2023

jbi.global

CRICOS Provider Number 00123M

Supplementary material has been published as submitted. It has not been copyedited, or typeset by Acta Oncologica

INTRODUCTION

JBI is a global organization promoting and supporting evidence-based decisions that improve health and health service delivery. JBI offers a unique range of solutions to access, appraise and apply the best available evidence, servicing over 90 countries. Working with 80+ universities, hospitals and NGOs from across the globe through the JBI Collaboration, JBI is a recognized global leader in evidence-based health care.

JBI Systematic Reviews

The core of evidence synthesis is the systematic review of literature of a particular intervention, condition or issue. The systematic review is essentially an analysis of the available evidence and a judgment of the effectiveness or otherwise of a practice, involving a series of complex steps. JBI takes a particular view on what counts as evidence and the methods utilized to synthesize those different types of evidence. In line with this broader view of evidence, JBI has developed theories, methodologies and rigorous processes for the critical appraisal and synthesis of these diverse forms of evidence in order to aid in clinical decision-making in health care. Guidance now exists for conducting reviews of effectiveness research, qualitative research, prevalence/incidence, etiology/risk, economic evaluations, text/opinion, diagnostic test accuracy, mixed-methods, umbrella reviews and scoping reviews. Further information regarding JBI systematic reviews can be found in the JBI Manual for Evidence Synthesis.

JBI Critical Appraisal Tools

All systematic reviews incorporate a process of critique or appraisal of the research evidence. The purpose of this appraisal for quantitative evidence is to determine the extent to which a study has addressed the possibility of bias in its design, conduct and analysis. All papers selected for inclusion in the systematic review (that is – those that meet the inclusion criteria described in the protocol) need to be subjected to rigorous appraisal by two critical appraisers. The results of this appraisal can then be used to inform synthesis and interpretation of the results of the study. Although designed for use in systematic reviews, JBI critical appraisal tools can also be used when creating Critically Appraised Topics, in journal clubs and as an educational tool.

How were these tools developed?

JBI critical appraisal tools have been developed by JBI and collaborators. The particular iteration of this tool was developed by the JBI Effectiveness Methodology Group following oversight by the JBI Scientific Committee.

Like the previous versions of these tools, this version presents signaling questions to prompt reviewers to identify whether certain safeguards of bias have been met, in the primary literature under review. However, unlike previous iterations of this tool, this version has separated questions into whether they provide an answer relating to internal, external or statistical conclusion validity. For questions related to internal validity, these have been further separated to identify what domain of bias they are referring. Finally, this tool has also been structured to facilitate judgments related to bias at different levels (e.g. bias at the outcome level or bias at the result level) where appropriate.

These tools have been approved following extensive peer review by the JBI Scientific Committee.

How to cite

Please use the following when citing this tool: Barker TH, Habibi N, Aromataris E, Stone JC, Leonardi-Bee J, Sears K, et al. The revised JBI critical appraisal tool for the assessment of risk of bias quasi-experimental studies. JBI Evid Synth. 2024;22(3):378-88.

**RoB Assessor: A.H. and J.S.**	**Date of Appraisal: 13.03.25**	**Record Number: doi: 10.1042/CS19990273**
**Study Author: Barber MD et al.**	**Study Title: Metabolic response to feeding in weight-losing pancreatic cancer patients and its modulation by a fish-oil-enriched nutritional supplement**	**Study Year: 2000**

Internal Validity		Choice - Comments/Justification	Yes	No	Unclear	N/A
**Bias related to temporal precedence**						
1	**Is it clear in the study what is the "cause" and what is the "effect" (i.e. there is no confusion about which variable comes first)?**		☒	☐	☐	☐
**Bias related to selection and allocation**						
2	**Was there a control group?**	Yes	☒	☐	☐	☐
**Bias related to confounding factors**						
3	**Were participants included in any comparisons similar?**	Yes, 16 cancer patients with unequivocal diagnosis of pancreatic cancer who were losing weight.	☒	☐	☐	☐
**Bias related to administration of intervention/exposure**						
4	**Were the participants included in any comparisons receiving similar treatment/care, other than the exposure or intervention of interest?**	The study is conducted before patients start cancer treatment	☐	☒	☐	☐

**Bias related to assessment, detection and measurement of the outcome**						
5	**Were there multiple measurements of the outcome, both pre and post the intervention/exposure?**		**Yes**	**No**	**Unclear**	**N/A**

	**Outcome 1**	Fasting levels of insulin and glucose at baseline measured by a blood test at day 1 of the study for all study participants	☐	☒	☐	☐
	**Outcome 2**		☐	☐	☐	☐
	**Outcome 3**		☐	☐	☐	☐
	**Outcome 4**		☐	☐	☐	☐
	**Outcome 5**		☐	☐	☐	☐
	**Outcome 6**		☐	☐	☐	☐
	**Outcome 7**		☐	☐	☐	☐

6	**Were the outcomes of participants included in any comparisons measured in the same way?**		**Yes**	**No**	**Unclear**	**N/A**
	**Outcome 1**	Fasting levels of insulin and glucose at baseline measured by a blood test at day 1 of the study for all study participants	☒	☐	☐	☐
	**Outcome 2**		☐	☐	☐	☐
	**Outcome 3**		☐	☐	☐	☐
	**Outcome 4**		☐	☐	☐	☐
	**Outcome 5**		☐	☐	☐	☐
	**Outcome 6**		☐	☐	☐	☐
	**Outcome 7**		☐	☐	☐	☐

7	Were outcomes measured in a reliable way?		Yes	No	Unclear	N/A
	**Outcome 1**	Fasting levels of insulin and glucose at baseline measured by a blood test at day 1 of the study for all study participants	☒	☐	☐	☐
	**Outcome 2**		☐	☐	☐	☐
	**Outcome 3**		☐	☐	☐	☐
	**Outcome 4**		☐	☐	☐	☐
	**Outcome 5**		☐	☐	☐	☐
	**Outcome 6**		☐	☐	☐	☐
	**Outcome 7**		☐	☐	☐	☐

Bias related to participant retention

8	Was follow-up complete and if not, were differences between groups in terms of their follow-up adequately described and analyzed?					
	**Outcome 1**	Follow up not relevant for outcome 1	**Yes**	**No**	**Unclear**	**N/A**
	Result 1		☐	☐	☐	☐
	Result 2		☐	☐	☐	☐
	Result 3		☐	☐	☐	☐
	**Outcome 2**		**Yes**	**No**	**Unclear**	**N/A**
	Result 1		☐	☐	☐	☐
	Result 2		☐	☐	☐	☐

Result 3		☐	☐	☐	☐
**Outcome 3**		**Yes**	**No**	**Unclear**	**N/A**
Result 1		☐	☐	☐	☐
Result 2		☐	☐	☐	☐
Result 3		☐	☐	☐	☐
**Outcome 4**		**Yes**	**No**	**Unclear**	**N/A**
Result 1		☐	☐	☐	☐
Result 2		☐	☐	☐	☐
Result 3		☐	☐	☐	☐
**Outcome 5**		**Yes**	**No**	**Unclear**	**N/A**
Result 1		☐	☐	☐	☐
Result 2		☐	☐	☐	☐
Result 3		☐	☐	☐	☐
**Outcome 6**		**Yes**	**No**	**Unclear**	**N/A**
Result 1		☐	☐	☐	☐
Result 2		☐	☐	☐	☐
Result 3		☐	☐	☐	☐
**Outcome 7**		**Yes**	**No**	**Unclear**	**N/A**
Result 1		☐	☐	☐	☐
Result 2		☐	☐	☐	☐

Result 3		☐	☐	☐	☐

Statistical Conclusion Validity

9	**Was appropriate statistical analysis used?**					
	**Outcome 1**	Statical analysis not relevant for outcome 1	**Yes**	**No**	**Unclear**	**N/A**
	Result 1		☐	☐	☐	☐
	Result 2		☐	☐	☐	☐
	Result 3		☐	☐	☐	☐
	**Outcome 2**		**Yes**	**No**	**Unclear**	**N/A**
	Result 1		☐	☐	☐	☐
	Result 2		☐	☐	☐	☐
	Result 3		☐	☐	☐	☐
	**Outcome 3**		**Yes**	**No**	**Unclear**	**N/A**
	Result 1		☐	☐	☐	☐
	Result 2		☐	☐	☐	☐
	Result 3		☐	☐	☐	☐
	**Outcome 4**		**Yes**	**No**	**Unclear**	**N/A**
	Result 1		☐	☐	☐	☐
	Result 2		☐	☐	☐	☐
	Result 3		☐	☐	☐	☐

Outcome 5		Yes	No	Unclear	N/A
Result 1		☐	☐	☐	☐
Result 2		☐	☐	☐	☐
Result 3		☐	☐	☐	☐
**Outcome 6**		**Yes**	**No**	**Unclear**	**N/A**
Result 1		☐	☐	☐	☐
Result 2		☐	☐	☐	☐
Result 3		☐	☐	☐	☐
**Outcome 7**		**Yes**	**No**	**Unclear**	**N/A**
Result 1		☐	☐	☐	☐
Result 2		☐	☐	☐	☐
Result 3		☐	☐	☐	☐

Overall appraisal: **Include:** ☒ **Exclude:** ☐ **Seek Further Info:** ☐

Comments:

For our review, we only extract data about fasting insulin and glucose levels at baseline for patients, and this study is appraised according to measurement of these parameters.

S4 JBI checklist

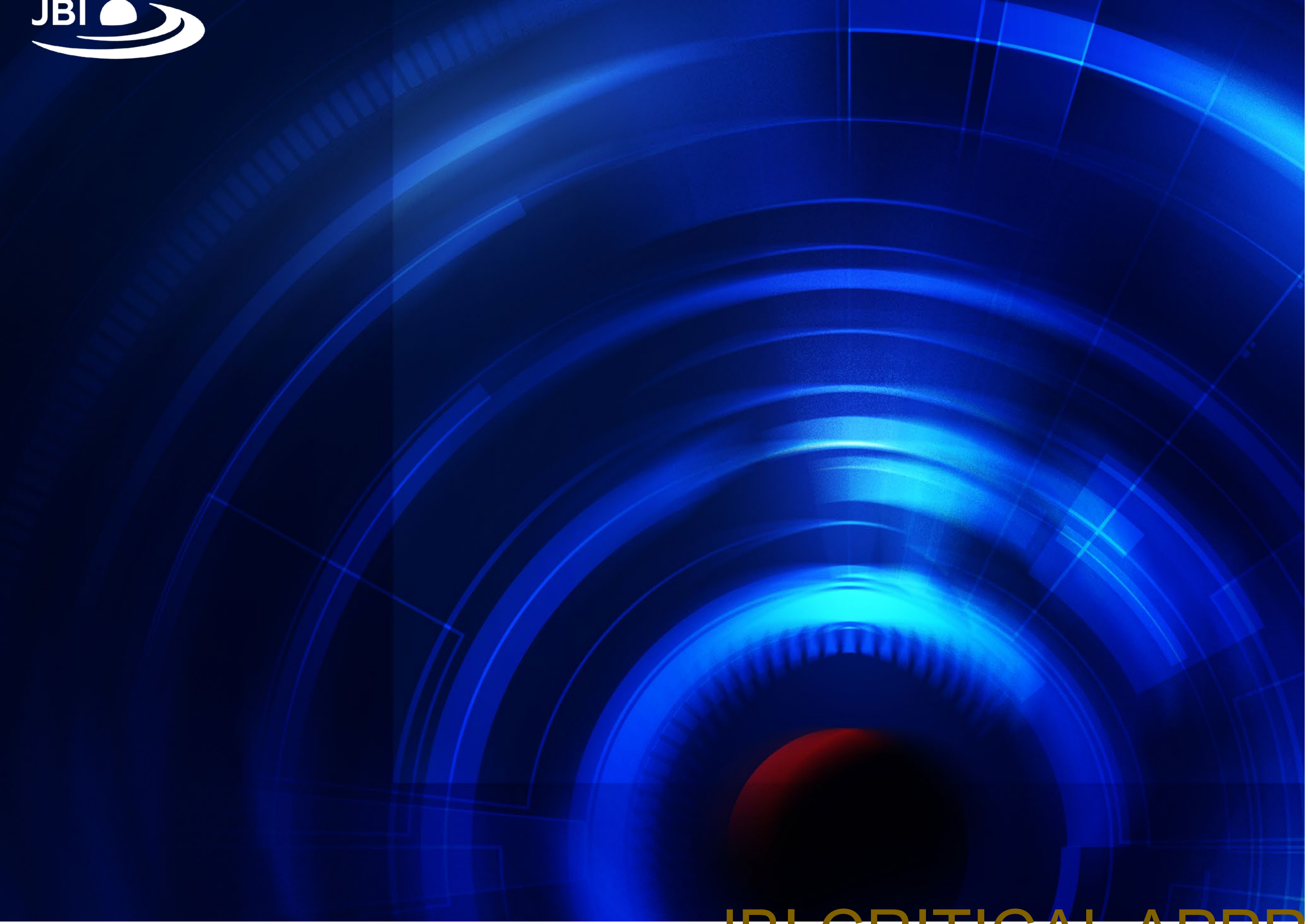

JBI CRITICAL APPRAISAL TOOL

JBI CHECKLIST FOR QUASI-EXPERIMENTAL STUDIES

2023

jbi.global

CRICOS Provider Number 00123M

INTRODUCTION

JBI is a global organization promoting and supporting evidence-based decisions that improve health and health service delivery. JBI offers a unique range of solutions to access, appraise and apply the best available evidence, servicing over 90 countries. Working with 80+ universities, hospitals and NGOs from across the globe through the JBI Collaboration, JBI is a recognized global leader in evidence-based health care.

JBI Systematic Reviews

The core of evidence synthesis is the systematic review of literature of a particular intervention, condition or issue. The systematic review is essentially an analysis of the available evidence and a judgment of the effectiveness or otherwise of a practice, involving a series of complex steps. JBI takes a particular view on what counts as evidence and the methods utilized to synthesize those different types of evidence. In line with this broader view of evidence, JBI has developed theories, methodologies and rigorous processes for the critical appraisal and synthesis of these diverse forms of evidence in order to aid in clinical decision-making in health care. Guidance now exists for conducting reviews of effectiveness research, qualitative research, prevalence/incidence, etiology/risk, economic evaluations, text/opinion, diagnostic test accuracy, mixed-methods, umbrella reviews and scoping reviews. Further information regarding JBI systematic reviews can be found in the JBI Manual for Evidence Synthesis.

JBI Critical Appraisal Tools

All systematic reviews incorporate a process of critique or appraisal of the research evidence. The purpose of this appraisal for quantitative evidence is to determine the extent to which a study has addressed the possibility of bias in its design, conduct and analysis. All papers selected for inclusion in the systematic review (that is – those that meet the inclusion criteria described in the protocol) need to be subjected to rigorous appraisal by two critical appraisers. The results of this appraisal can then be used to inform synthesis and interpretation of the results of the study. Although designed for use in systematic reviews, JBI critical appraisal tools can also be used when creating Critically Appraised Topics, in journal clubs and as an educational tool.

How were these tools developed?

JBI critical appraisal tools have been developed by JBI and collaborators. The particular iteration of this tool was developed by the JBI Effectiveness Methodology Group following oversight by the JBI Scientific Committee.

Like the previous versions of these tools, this version presents signaling questions to prompt reviewers to identify whether certain safeguards of bias have been met, in the primary literature under review. However, unlike previous iterations of this tool, this version has separated questions into whether they provide an answer relating to internal, external or statistical conclusion validity. For questions related to internal validity, these have been further separated to identify what domain of bias they are referring. Finally, this tool has also been structured to facilitate judgments related to bias at different levels (e.g. bias at the outcome level or bias at the result level) where appropriate.

These tools have been approved following extensive peer review by the JBI Scientific Committee.

How to cite

Please use the following when citing this tool: Barker TH, Habibi N, Aromataris E, Stone JC, Leonardi-Bee J, Sears K, et al. The revised JBI critical appraisal tool for the assessment of risk of bias quasi-experimental studies. JBI Evid Synth. 2024;22(3):378-88.

**RoB Assessor: A.H. and J.S.**	**Date of Appraisal: 13.03.25**	**Record Number: doi:10.1016/S0016-5085(83)80234-0**
**Study Author: Bennegård et al.**	**Study Title: Metabolic Balance across the Leg in Weight-losing Cancer Patients Compared to Depleted Patients without Cancer**	**Study Year: 1982**

Internal Validity		Choice - Comments/Justification	Yes	No	Unclear	N/A
**Bias related to temporal precedence**						
1	**Is it clear in the study what is the "cause" and what is the "effect" (i.e. there is no confusion about which variable comes first)?**		☒	☐	☐	☐
**Bias related to selection and allocation**						
2	**Was there a control group?**	Yes three control groups	☒	☐	☐	☐
**Bias related to confounding factors**						
3	**Were participants included in any comparisons similar?**	Yes, 19 weight-losing cancer patients	☒	☐	☐	☐
**Bias related to administration of intervention/exposure**						
4	**Were the participants included in any comparisons receiving similar treatment/care, other than the exposure or intervention of interest?**	None of the cancer patients received any cancer treatment before the study.	☐	☒	☐	☐

Bias related to assessment, detection and measurement of the outcome			Yes	No	Unclear	N/A
5	**Were there multiple measurements of the outcome, both pre and post the intervention/exposure?**					

	**Outcome 1**	Fasting levels of insulin and glucose at baseline measured by a blood test at day 1 of the study for all study participants	☐	☒	☐	☐
	**Outcome 2**		☐	☐	☐	☐
	**Outcome 3**		☐	☐	☐	☐
	**Outcome 4**		☐	☐	☐	☐
	**Outcome 5**		☐	☐	☐	☐
	**Outcome 6**		☐	☐	☐	☐
	**Outcome 7**		☐	☐	☐	☐

6	**Were the outcomes of participants included in any comparisons measured in the same way?**		**Yes**	**No**	**Unclear**	**N/A**
	**Outcome 1**	Fasting levels of insulin and glucose at baseline measured by a blood test at day 1 of the study for all study participants	☒	☐	☐	☐
	**Outcome 2**		☐	☐	☐	☐
	**Outcome 3**		☐	☐	☐	☐
	**Outcome 4**		☐	☐	☐	☐
	**Outcome 5**		☐	☐	☐	☐
	**Outcome 6**		☐	☐	☐	☐
	**Outcome 7**		☐	☐	☐	☐

7	Were outcomes measured in a reliable way?		Yes	No	Unclear	N/A
	**Outcome 1**	Fasting levels of insulin and glucose at baseline measured by a blood test at day 1 of the study for all study participants	☒	☐	☐	☐
	**Outcome 2**		☐	☐	☐	☐
	**Outcome 3**		☐	☐	☐	☐
	**Outcome 4**		☐	☐	☐	☐
	**Outcome 5**		☐	☐	☐	☐
	**Outcome 6**		☐	☐	☐	☐
	**Outcome 7**		☐	☐	☐	☐

Bias related to participant retention

8	Was follow-up complete and if not, were differences between groups in terms of their follow-up adequately described and analyzed?	Follow up not relevant for outcome 1				
	**Outcome 1**		**Yes**	**No**	**Unclear**	**N/A**
	Result 1		☐	☐	☐	☐
	Result 2		☐	☐	☐	☐
	Result 3		☐	☐	☐	☐
	**Outcome 2**		**Yes**	**No**	**Unclear**	**N/A**
	Result 1		☐	☐	☐	☐
	Result 2		☐	☐	☐	☐

Result 3		☐	☐	☐	☐
**Outcome 3**		**Yes**	**No**	**Unclear**	**N/A**
Result 1		☐	☐	☐	☐
Result 2		☐	☐	☐	☐
Result 3		☐	☐	☐	☐
**Outcome 4**		**Yes**	**No**	**Unclear**	**N/A**
Result 1		☐	☐	☐	☐
Result 2		☐	☐	☐	☐
Result 3		☐	☐	☐	☐
**Outcome 5**		**Yes**	**No**	**Unclear**	**N/A**
Result 1		☐	☐	☐	☐
Result 2		☐	☐	☐	☐
Result 3		☐	☐	☐	☐
**Outcome 6**		**Yes**	**No**	**Unclear**	**N/A**
Result 1		☐	☐	☐	☐
Result 2		☐	☐	☐	☐
Result 3		☐	☐	☐	☐
**Outcome 7**		**Yes**	**No**	**Unclear**	**N/A**
Result 1		☐	☐	☐	☐
Result 2		☐	☐	☐	☐

Result 3		☐	☐	☐	☐

Statistical Conclusion Validity

9	Was appropriate statistical analysis used?	Statical analysis not relevant for outcome 1				
	**Outcome 1**		**Yes**	**No**	**Unclear**	**N/A**
	Result 1		☐	☐	☐	☐
	Result 2		☐	☐	☐	☐
	Result 3		☐	☐	☐	☐
	**Outcome 2**		**Yes**	**No**	**Unclear**	**N/A**
	Result 1		☐	☐	☐	☐
	Result 2		☐	☐	☐	☐
	Result 3		☐	☐	☐	☐
	**Outcome 3**		**Yes**	**No**	**Unclear**	**N/A**
	Result 1		☐	☐	☐	☐
	Result 2		☐	☐	☐	☐
	Result 3		☐	☐	☐	☐
	**Outcome 4**		**Yes**	**No**	**Unclear**	**N/A**
	Result 1		☐	☐	☐	☐
	Result 2		☐	☐	☐	☐
	Result 3		☐	☐	☐	☐

**Outcome 5**		**Yes**	**No**	**Unclear**	**N/A**
Result 1		☐	☐	☐	☐
Result 2		☐	☐	☐	☐
Result 3		☐	☐	☐	☐
**Outcome 6**		**Yes**	**No**	**Unclear**	**N/A**
Result 1		☐	☐	☐	☐
Result 2		☐	☐	☐	☐
Result 3		☐	☐	☐	☐
**Outcome 7**		**Yes**	**No**	**Unclear**	**N/A**
Result 1		☐	☐	☐	☐
Result 2		☐	☐	☐	☐
Result 3		☐	☐	☐	☐

Overall appraisal: **Include:** ☐ **Exclude:** ☐ **Seek Further Info:** ☐

Comments:

For our review, we only extract data about fasting insulin and glucose levels at baseline for patients, and this study is appraised according to measurement of these parameters.

S5 JBI checklist

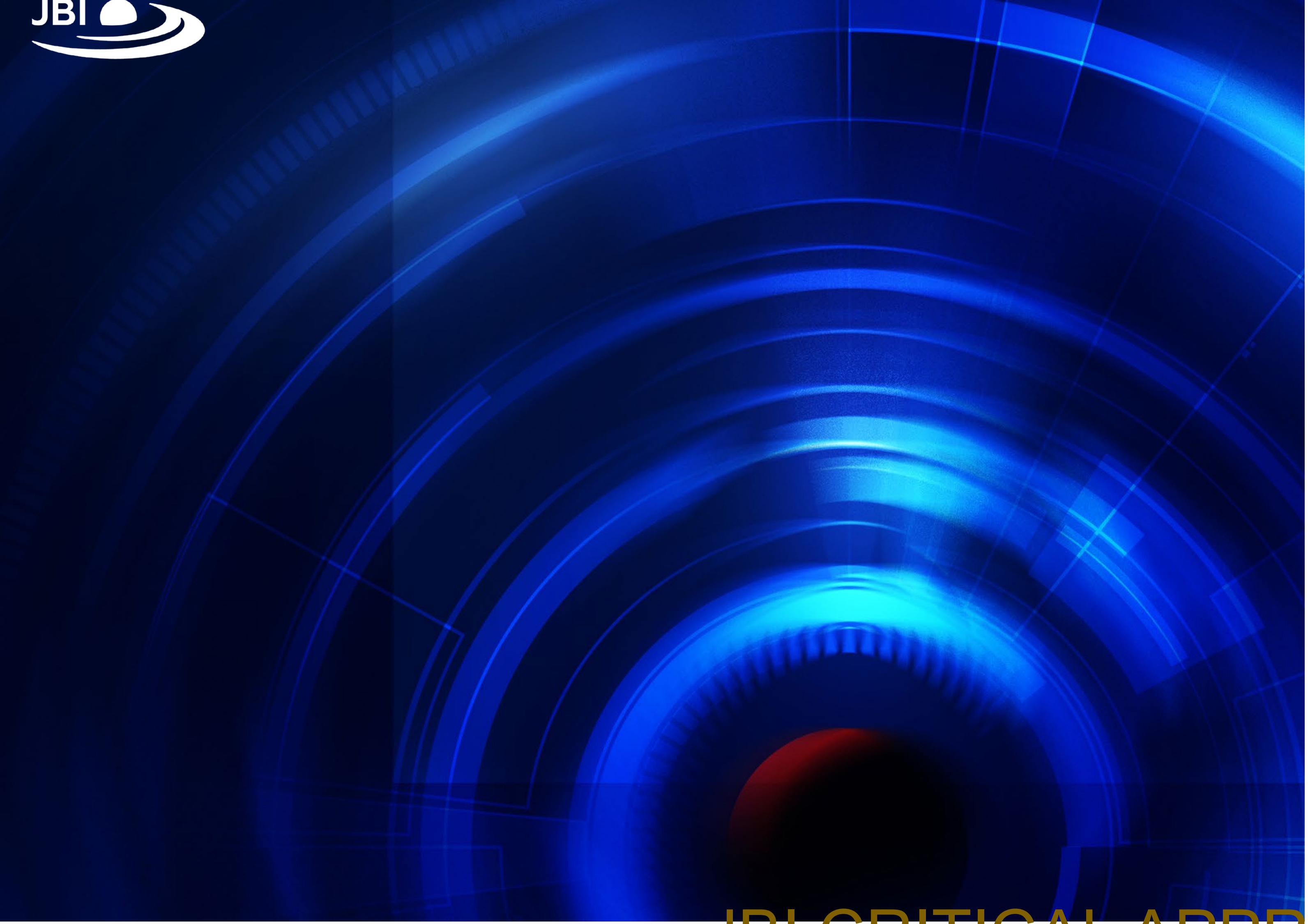

JBI CRITICAL APPRAISAL TOOL

JBI CHECKLIST FOR QUASI-EXPERIMENTAL STUDIES

2023

jbi.global

CRICOS Provider Number 00123M

INTRODUCTION

JBI is a global organization promoting and supporting evidence-based decisions that improve health and health service delivery. JBI offers a unique range of solutions to access, appraise and apply the best available evidence, servicing over 90 countries. Working with 80+ universities, hospitals and NGOs from across the globe through the JBI Collaboration, JBI is a recognized global leader in evidence-based health care.

JBI Systematic Reviews

The core of evidence synthesis is the systematic review of literature of a particular intervention, condition or issue. The systematic review is essentially an analysis of the available evidence and a judgment of the effectiveness or otherwise of a practice, involving a series of complex steps. JBI takes a particular view on what counts as evidence and the methods utilized to synthesize those different types of evidence. In line with this broader view of evidence, JBI has developed theories, methodologies and rigorous processes for the critical appraisal and synthesis of these diverse forms of evidence in order to aid in clinical decision-making in health care. Guidance now exists for conducting reviews of effectiveness research, qualitative research, prevalence/incidence, etiology/risk, economic evaluations, text/opinion, diagnostic test accuracy, mixed-methods, umbrella reviews and scoping reviews. Further information regarding JBI systematic reviews can be found in the JBI Manual for Evidence Synthesis.

JBI Critical Appraisal Tools

All systematic reviews incorporate a process of critique or appraisal of the research evidence. The purpose of this appraisal for quantitative evidence is to determine the extent to which a study has addressed the possibility of bias in its design, conduct and analysis. All papers selected for inclusion in the systematic review (that is – those that meet the inclusion criteria described in the protocol) need to be subjected to rigorous appraisal by two critical appraisers. The results of this appraisal can then be used to inform synthesis and interpretation of the results of the study. Although designed for use in systematic reviews, JBI critical appraisal tools can also be used when creating Critically Appraised Topics, in journal clubs and as an educational tool.

How were these tools developed?

JBI critical appraisal tools have been developed by JBI and collaborators. The particular iteration of this tool was developed by the JBI Effectiveness Methodology Group following oversight by the JBI Scientific Committee.

Like the previous versions of these tools, this version presents signaling questions to prompt reviewers to identify whether certain safeguards of bias have been met, in the primary literature under review. However, unlike previous iterations of this tool, this version has separated questions into whether they provide an answer relating to internal, external or statistical conclusion validity. For questions related to internal validity, these have been further separated to identify what domain of bias they are referring. Finally, this tool has also been structured to facilitate judgments related to bias at different levels (e.g. bias at the outcome level or bias at the result level) where appropriate.

These tools have been approved following extensive peer review by the JBI Scientific Committee.

How to cite

Please use the following when citing this tool: Barker TH, Habibi N, Aromataris E, Stone JC, Leonardi-Bee J, Sears K, et al. The revised JBI critical appraisal tool for the assessment of risk of bias quasi-experimental studies. JBI Evid Synth. 2024;22(3):378-88.

RoB Assessor: A.H. and J.S.	Date of Appraisal: 13.03.25	Record Number: doi:10.1016/S0016-5085(83)80234-0
Study Author: Bennegård et. al	Study Title: Metabolic Response of Whole Body and Peripheral Tissues to Enteral Nutrition in Weight-Losing Cancer and Noncancer Patients	Study Year: 1983

Internal Validity		Choice - Comments/Justification	Yes	No	Unclear	N/A
**Bias related to temporal precedence**						
1	Is it clear in the study what is the "cause" and what is the "effect" (i.e. there is no confusion about which variable comes first)?		☒	☐	☐	☐
**Bias related to selection and allocation**						
2	Was there a control group?		☒	☐	☐	☐
**Bias related to confounding factors**						
3	Were participants included in any comparisons similar?	8 weight-losing cancer patients	☒	☐	☐	☐
**Bias related to administration of intervention/exposure**						
4	Were the participants included in any comparisons receiving similar treatment/care, other than the exposure or intervention of interest?	The study is conducted before patients start cancer treatment	☐	☒	☐	☐

**Bias related to assessment, detection and measurement of the outcome**						
5	Were there multiple measurements of the outcome, both pre and post the intervention/exposure?		Yes	No	Unclear	N/A

	**Outcome 1**	Fasting levels of insulin and glucose at baseline measured by a blood test at day 1 of the study for all study participants	☐	☒	☐	☐
	**Outcome 2**		☐	☐	☐	☐
	**Outcome 3**		☐	☐	☐	☐
	**Outcome 4**		☐	☐	☐	☐
	**Outcome 5**		☐	☐	☐	☐
	**Outcome 6**		☐	☐	☐	☐
	**Outcome 7**		☐	☐	☐	☐

6	**Were the outcomes of participants included in any comparisons measured in the same way?**		**Yes**	**No**	**Unclear**	**N/A**
	**Outcome 1**	Fasting levels of insulin and glucose at baseline measured by a blood test at day 1 of the study for all study participants	☒	☐	☐	☐
	**Outcome 2**		☐	☐	☐	☐
	**Outcome 3**		☐	☐	☐	☐
	**Outcome 4**		☐	☐	☐	☐
	**Outcome 5**		☐	☐	☐	☐
	**Outcome 6**		☐	☐	☐	☐
	**Outcome 7**		☐	☐	☐	☐

7	Were outcomes measured in a reliable way?		Yes	No	Unclear	N/A
	**Outcome 1**	Fasting levels of insulin and glucose at baseline measured by a blood test at day 1 of the study for all study participants	☒	☐	☐	☐
	**Outcome 2**		☐	☐	☐	☐
	**Outcome 3**		☐	☐	☐	☐
	**Outcome 4**		☐	☐	☐	☐
	**Outcome 5**		☐	☐	☐	☐
	**Outcome 6**		☐	☐	☐	☐
	**Outcome 7**		☐	☐	☐	☐

Bias related to participant retention

8	Was follow-up complete and if not, were differences between groups in terms of their follow-up adequately described and analyzed?					
	**Outcome 1**	Follow up not relevant for outcome 1	**Yes**	**No**	**Unclear**	**N/A**
	Result 1		☐	☐	☐	☐
	Result 2		☐	☐	☐	☐
	Result 3		☐	☐	☐	☐
	**Outcome 2**		**Yes**	**No**	**Unclear**	**N/A**
	Result 1		☐	☐	☐	☐
	Result 2		☐	☐	☐	☐

		Yes	No	Unclear	N/A
Result 3		☐	☐	☐	☐
**Outcome 3**		**Yes**	**No**	**Unclear**	**N/A**
Result 1		☐	☐	☐	☐
Result 2		☐	☐	☐	☐
Result 3		☐	☐	☐	☐
**Outcome 4**		**Yes**	**No**	**Unclear**	**N/A**
Result 1		☐	☐	☐	☐
Result 2		☐	☐	☐	☐
Result 3		☐	☐	☐	☐
**Outcome 5**		**Yes**	**No**	**Unclear**	**N/A**
Result 1		☐	☐	☐	☐
Result 2		☐	☐	☐	☐
Result 3		☐	☐	☐	☐
**Outcome 6**		**Yes**	**No**	**Unclear**	**N/A**
Result 1		☐	☐	☐	☐
Result 2		☐	☐	☐	☐
Result 3		☐	☐	☐	☐
**Outcome 7**		**Yes**	**No**	**Unclear**	**N/A**
Result 1		☐	☐	☐	☐
Result 2		☐	☐	☐	☐

Result 3		☐	☐	☐	☐

Statistical Conclusion Validity

9	**Was appropriate statistical analysis used?**					
	**Outcome 1**	Statical analysis not relevant for outcome 1	**Yes**	**No**	**Unclear**	**N/A**
	Result 1		☐	☐	☐	☐
	Result 2		☐	☐	☐	☐
	Result 3		☐	☐	☐	☐
	**Outcome 2**		**Yes**	**No**	**Unclear**	**N/A**
	Result 1		☐	☐	☐	☐
	Result 2		☐	☐	☐	☐
	Result 3		☐	☐	☐	☐
	**Outcome 3**		**Yes**	**No**	**Unclear**	**N/A**
	Result 1		☐	☐	☐	☐
	Result 2		☐	☐	☐	☐
	Result 3		☐	☐	☐	☐
	**Outcome 4**		**Yes**	**No**	**Unclear**	**N/A**
	Result 1		☐	☐	☐	☐
	Result 2		☐	☐	☐	☐
	Result 3		☐	☐	☐	☐

**Outcome 5**		**Yes**	**No**	**Unclear**	**N/A**
Result 1		☐	☐	☐	☐
Result 2		☐	☐	☐	☐
Result 3		☐	☐	☐	☐
**Outcome 6**		**Yes**	**No**	**Unclear**	**N/A**
Result 1		☐	☐	☐	☐
Result 2		☐	☐	☐	☐
Result 3		☐	☐	☐	☐
**Outcome 7**		**Yes**	**No**	**Unclear**	**N/A**
Result 1		☐	☐	☐	☐
Result 2		☐	☐	☐	☐
Result 3		☐	☐	☐	☐

Overall appraisal: **Include:** ☒ **Exclude:** ☐ **Seek Further Info:** ☐

Comments:

For our review, we only extract data about fasting insulin and glucose levels at baseline for patients, and this study is appraised according to measurement of these parameters.

S6 JBI checklist

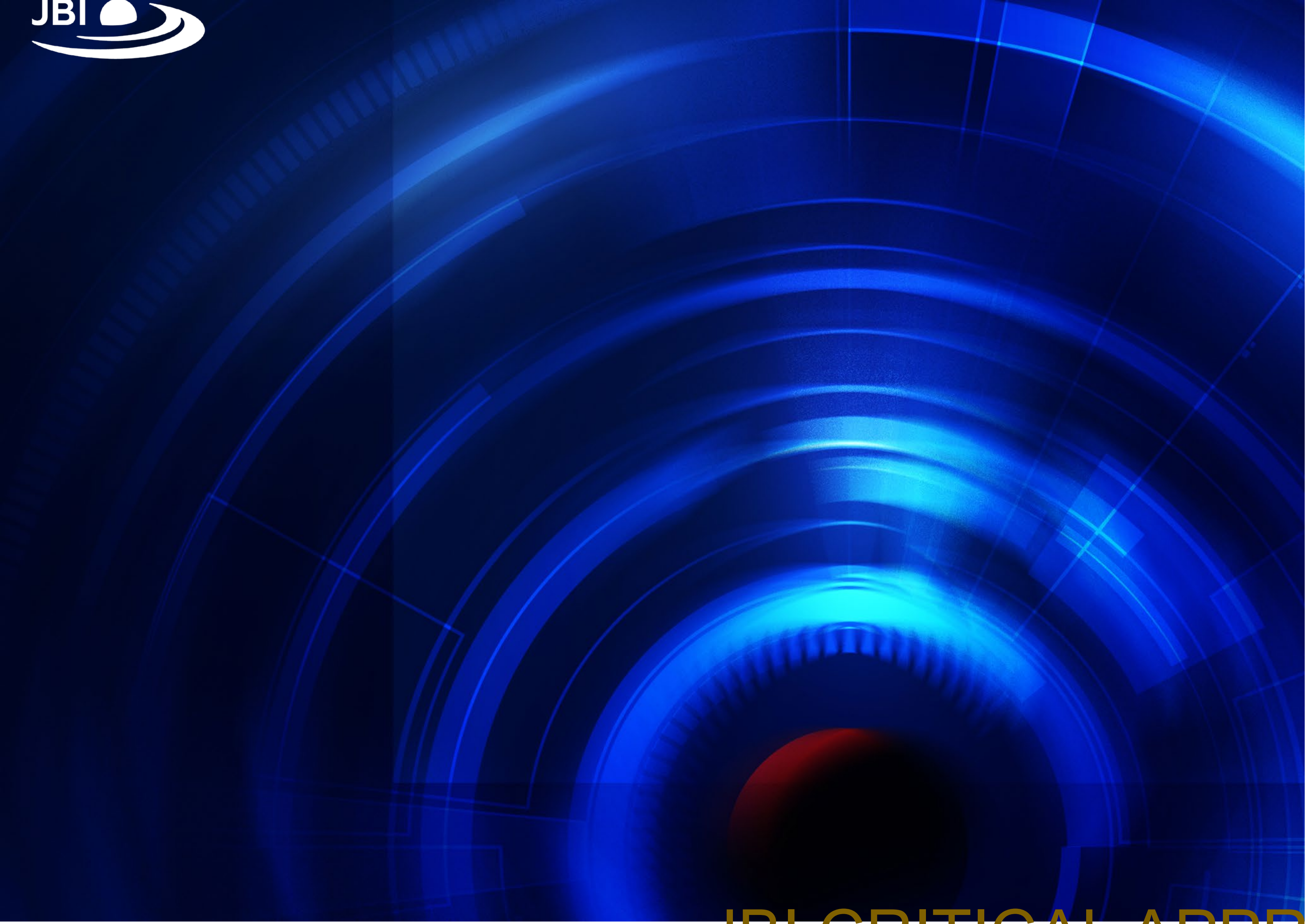

JBI CRITICAL APPRAISAL TOOL

JBI CHECKLIST FOR QUASI-EXPERIMENTAL STUDIES

2023

jbi.global

CRICOS Provider Number 00123M

INTRODUCTION

JBI is a global organization promoting and supporting evidence-based decisions that improve health and health service delivery. JBI offers a unique range of solutions to access, appraise and apply the best available evidence, servicing over 90 countries. Working with 80+ universities, hospitals and NGOs from across the globe through the JBI Collaboration, JBI is a recognized global leader in evidence-based health care.

JBI Systematic Reviews

The core of evidence synthesis is the systematic review of literature of a particular intervention, condition or issue. The systematic review is essentially an analysis of the available evidence and a judgment of the effectiveness or otherwise of a practice, involving a series of complex steps. JBI takes a particular view on what counts as evidence and the methods utilized to synthesize those different types of evidence. In line with this broader view of evidence, JBI has developed theories, methodologies and rigorous processes for the critical appraisal and synthesis of these diverse forms of evidence in order to aid in clinical decision-making in health care. Guidance now exists for conducting reviews of effectiveness research, qualitative research, prevalence/incidence, etiology/risk, economic evaluations, text/opinion, diagnostic test accuracy, mixed-methods, umbrella reviews and scoping reviews. Further information regarding JBI systematic reviews can be found in the JBI Manual for Evidence Synthesis.

JBI Critical Appraisal Tools

All systematic reviews incorporate a process of critique or appraisal of the research evidence. The purpose of this appraisal for quantitative evidence is to determine the extent to which a study has addressed the possibility of bias in its design, conduct and analysis. All papers selected for inclusion in the systematic review (that is – those that meet the inclusion criteria described in the protocol) need to be subjected to rigorous appraisal by two critical appraisers. The results of this appraisal can then be used to inform synthesis and interpretation of the results of the study. Although designed for use in systematic reviews, JBI critical appraisal tools can also be used when creating Critically Appraised Topics, in journal clubs and as an educational tool.

How were these tools developed?

JBI critical appraisal tools have been developed by JBI and collaborators. The particular iteration of this tool was developed by the JBI Effectiveness Methodology Group following oversight by the JBI Scientific Committee.

Like the previous versions of these tools, this version presents signaling questions to prompt reviewers to identify whether certain safeguards of bias have been met, in the primary literature under review. However, unlike previous iterations of this tool, this version has separated questions into whether they provide an answer relating to internal, external or statistical conclusion validity. For questions related to internal validity, these have been further separated to identify what domain of bias they are referring. Finally, this tool has also been structured to facilitate judgments related to bias at different levels (e.g. bias at the outcome level or bias at the result level) where appropriate.

These tools have been approved following extensive peer review by the JBI Scientific Committee.

How to cite

Please use the following when citing this tool: Barker TH, Habibi N, Aromataris E, Stone JC, Leonardi-Bee J, Sears K, et al. The revised JBI critical appraisal tool for the assessment of risk of bias quasi-experimental studies. JBI Evid Synth. 2024;22(3):378-88.

RoB Assessor: A.H. and J.S.	Date of Appraisal: 13.03.25	Record Number: doi.org/10.1111/j.1475-097x.1986.tb00787.x
Study Author: Bennegård et al.	Study Title: Mechanisms of insulin resistance in cancer associated malnutrition.	Study Year: 1986

	Internal Validity	Choice - Comments/Justification	Yes	No	Unclear	N/A
**Bias related to temporal precedence**						
1	Is it clear in the study what is the "cause" and what is the "effect" (i.e. there is no confusion about which variable comes first)?		☒	☐	☐	☐
**Bias related to selection and allocation**						
2	Was there a control group?		☒	☐	☐	☐
**Bias related to confounding factors**						
3	Were participants included in any comparisons similar?	Yes, Five weight-losing male cancer patients	☒	☐	☐	☐
**Bias related to administration of intervention/exposure**						
4	Were the participants included in any comparisons receiving similar treatment/care, other than the exposure or intervention of interest?	None of the cancer patients received any cancer treatment before the study.	☐	☒	☐	☐

	Bias related to assessment, detection and measurement of the outcome		Yes	No	Unclear	N/A
5	Were there multiple measurements of the outcome, both pre and post the intervention/exposure?					
	Outcome 1	Fasting levels of insulin and glucose at baseline measured by a blood test at day 1 of the study for all study participants	☐	☒	☐	☐

	**Outcome 2**		☐	☐	☐	☐
	**Outcome 3**		☐	☐	☐	☐
	**Outcome 4**		☐	☐	☐	☐
	**Outcome 5**		☐	☐	☐	☐
	**Outcome 6**		☐	☐	☐	☐
	**Outcome 7**		☐	☐	☐	☐

6	**Were the outcomes of participants included in any comparisons measured in the same way?**		**Yes**	**No**	**Unclear**	**N/A**
	**Outcome 1**	Fasting levels of insulin and glucose at baseline measured by a blood test at day 1 of the study for all study participants	☒	☐	☐	☐
	**Outcome 2**		☐	☐	☐	☐
	**Outcome 3**		☐	☐	☐	☐
	**Outcome 4**		☐	☐	☐	☐
	**Outcome 5**		☐	☐	☐	☐
	**Outcome 6**		☐	☐	☐	☐
	**Outcome 7**		☐	☐	☐	☐

7	**Were outcomes measured in a reliable way?**		**Yes**	**No**	**Unclear**	**N/A**

**Outcome 1**	Fasting levels of insulin and glucose at baseline measured by a blood test at day 1 of the study for all study participants	☒	☐	☐	☐
**Outcome 2**		☐	☐	☐	☐
**Outcome 3**		☐	☐	☐	☐
**Outcome 4**		☐	☐	☐	☐
**Outcome 5**		☐	☐	☐	☐
**Outcome 6**		☐	☐	☐	☐
**Outcome 7**		☐	☐	☐	☐

Bias related to participant retention

8	**Was follow-up complete and if not, were differences between groups in terms of their follow-up adequately described and analyzed?**	Follow up not relevant for outcome 1				
	**Outcome 1**		**Yes**	**No**	**Unclear**	**N/A**
	Result 1		☐	☐	☐	☐
	Result 2		☐	☐	☐	☐
	Result 3		☐	☐	☐	☐
	**Outcome 2**		**Yes**	**No**	**Unclear**	**N/A**
	Result 1		☐	☐	☐	☐
	Result 2		☐	☐	☐	☐
	Result 3		☐	☐	☐	☐

Outcome 3		Yes	No	Unclear	N/A
Result 1		☐	☐	☐	☐
Result 2		☐	☐	☐	☐
Result 3		☐	☐	☐	☐
**Outcome 4**		**Yes**	**No**	**Unclear**	**N/A**
Result 1		☐	☐	☐	☐
Result 2		☐	☐	☐	☐
Result 3		☐	☐	☐	☐
**Outcome 5**		**Yes**	**No**	**Unclear**	**N/A**
Result 1		☐	☐	☐	☐
Result 2		☐	☐	☐	☐
Result 3		☐	☐	☐	☐
**Outcome 6**		**Yes**	**No**	**Unclear**	**N/A**
Result 1		☐	☐	☐	☐
Result 2		☐	☐	☐	☐
Result 3		☐	☐	☐	☐
**Outcome 7**		**Yes**	**No**	**Unclear**	**N/A**
Result 1		☐	☐	☐	☐
Result 2		☐	☐	☐	☐
Result 3		☐	☐	☐	☐

Statistical Conclusion Validity

9	Was appropriate statistical analysis used?	Statical analysis not relevant for outcome 1				
	**Outcome 1**		**Yes**	**No**	**Unclear**	**N/A**
	Result 1		☐	☐	☐	☐
	Result 2		☐	☐	☐	☐
	Result 3		☐	☐	☐	☐
	**Outcome 2**		**Yes**	**No**	**Unclear**	**N/A**
	Result 1		☐	☐	☐	☐
	Result 2		☐	☐	☐	☐
	Result 3		☐	☐	☐	☐
	**Outcome 3**		**Yes**	**No**	**Unclear**	**N/A**
	Result 1		☐	☐	☐	☐
	Result 2		☐	☐	☐	☐
	Result 3		☐	☐	☐	☐
	**Outcome 4**		**Yes**	**No**	**Unclear**	**N/A**
	Result 1		☐	☐	☐	☐
	Result 2		☐	☐	☐	☐
	Result 3		☐	☐	☐	☐
	**Outcome 5**		**Yes**	**No**	**Unclear**	**N/A**

Result 1		☐	☐	☐	☐
Result 2		☐	☐	☐	☐
Result 3		☐	☐	☐	☐
**Outcome 6**		**Yes**	**No**	**Unclear**	**N/A**
Result 1		☐	☐	☐	☐
Result 2		☐	☐	☐	☐
Result 3		☐	☐	☐	☐
**Outcome 7**		**Yes**	**No**	**Unclear**	**N/A**
Result 1		☐	☐	☐	☐
Result 2		☐	☐	☐	☐
Result 3		☐	☐	☐	☐

Overall appraisal: **Include:** ☐ **Exclude:** ☐ **Seek Further Info:** ☐

Comments:

For our review, we only extract data about fasting insulin and glucose levels at baseline for patients, and this study is appraised according to measurement of these parameters.

S7 JBI checklist

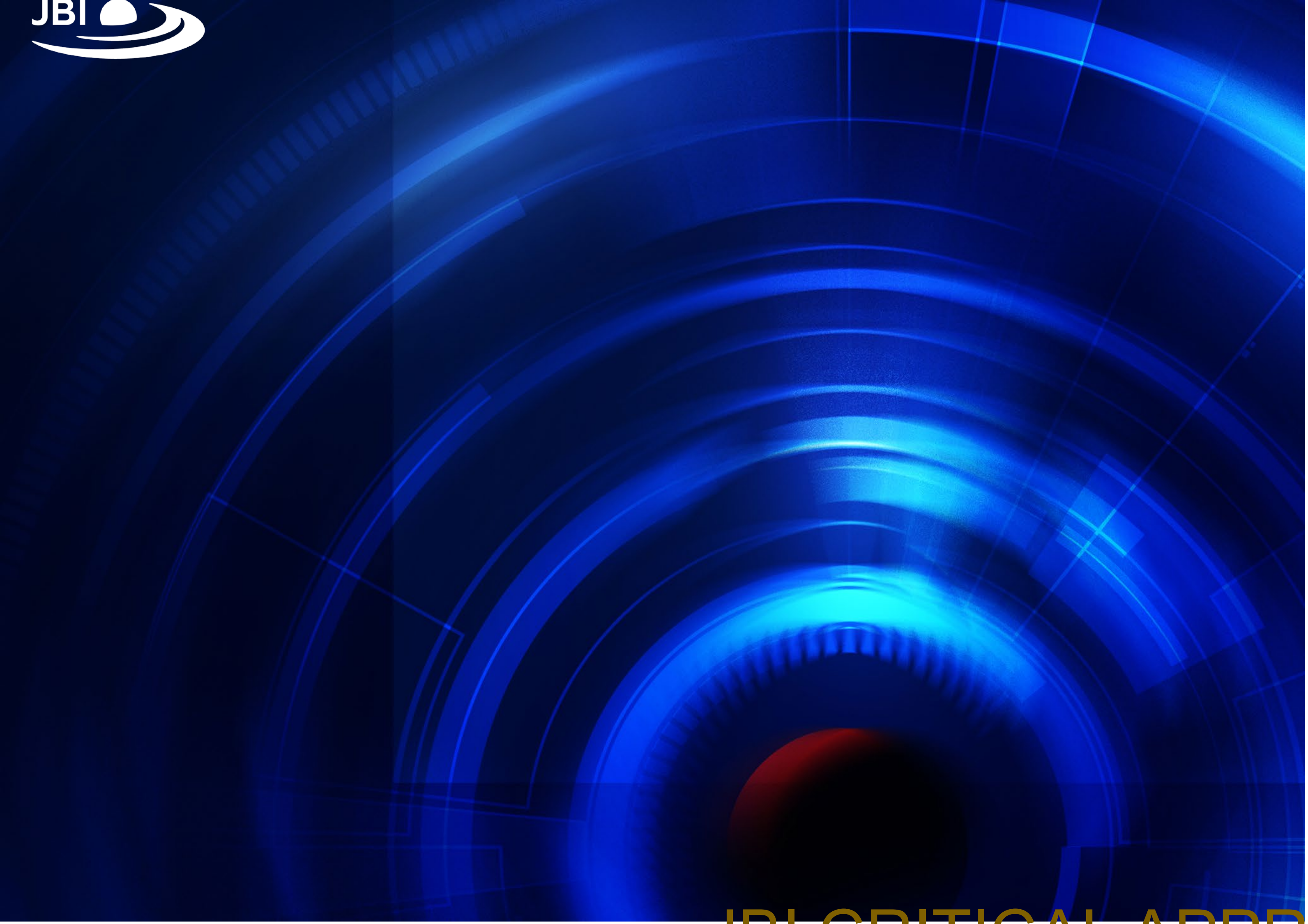

JBI CRITICAL APPRAISAL TOOL

JBI CHECKLIST FOR QUASI-EXPERIMENTAL STUDIES

2023

jbi.global

CRICOS Provider Number 00123M

INTRODUCTION

JBI is a global organization promoting and supporting evidence-based decisions that improve health and health service delivery. JBI offers a unique range of solutions to access, appraise and apply the best available evidence, servicing over 90 countries. Working with 80+ universities, hospitals and NGOs from across the globe through the JBI Collaboration, JBI is a recognized global leader in evidence-based health care.

JBI Systematic Reviews

The core of evidence synthesis is the systematic review of literature of a particular intervention, condition or issue. The systematic review is essentially an analysis of the available evidence and a judgment of the effectiveness or otherwise of a practice, involving a series of complex steps. JBI takes a particular view on what counts as evidence and the methods utilized to synthesize those different types of evidence. In line with this broader view of evidence, JBI has developed theories, methodologies and rigorous processes for the critical appraisal and synthesis of these diverse forms of evidence in order to aid in clinical decision-making in health care. Guidance now exists for conducting reviews of effectiveness research, qualitative research, prevalence/incidence, etiology/risk, economic evaluations, text/opinion, diagnostic test accuracy, mixed-methods, umbrella reviews and scoping reviews. Further information regarding JBI systematic reviews can be found in the JBI Manual for Evidence Synthesis.

JBI Critical Appraisal Tools

All systematic reviews incorporate a process of critique or appraisal of the research evidence. The purpose of this appraisal for quantitative evidence is to determine the extent to which a study has addressed the possibility of bias in its design, conduct and analysis. All papers selected for inclusion in the systematic review (that is – those that meet the inclusion criteria described in the protocol) need to be subjected to rigorous appraisal by two critical appraisers. The results of this appraisal can then be used to inform synthesis and interpretation of the results of the study. Although designed for use in systematic reviews, JBI critical appraisal tools can also be used when creating Critically Appraised Topics, in journal clubs and as an educational tool.

How were these tools developed?

JBI critical appraisal tools have been developed by JBI and collaborators. The particular iteration of this tool was developed by the JBI Effectiveness Methodology Group following oversight by the JBI Scientific Committee.

Like the previous versions of these tools, this version presents signaling questions to prompt reviewers to identify whether certain safeguards of bias have been met, in the primary literature under review. However, unlike previous iterations of this tool, this version has separated questions into whether they provide an answer relating to internal, external or statistical conclusion validity. For questions related to internal validity, these have been further separated to identify what domain of bias they are referring. Finally, this tool has also been structured to facilitate judgments related to bias at different levels (e.g. bias at the outcome level or bias at the result level) where appropriate.

These tools have been approved following extensive peer review by the JBI Scientific Committee.

How to cite

Please use the following when citing this tool: Barker TH, Habibi N, Aromataris E, Stone JC, Leonardi-Bee J, Sears K, et al. The revised JBI critical appraisal tool for the assessment of risk of bias quasi-experimental studies. JBI Evid Synth. 2024;22(3):378-88.

RoB Assessor: A.H. and J.S.	Date of Appraisal: 13.03.25	Record Number: PMID: 6357116
Study Author: Burt ME et al.	Study Title: Peripheral Tissue Metabolism in Cancer-bearing Man	Study Year: 1983

	Internal Validity	Choice - Comments/Justification	Yes	No	Unclear	N/A
**Bias related to temporal precedence**						
1	**Is it clear in the study what is the "cause" and what is the "effect" (i.e. there is no confusion about which variable comes first)?**		☒	☐	☐	☐
**Bias related to selection and allocation**						
2	**Was there a control group?**		☒	☐	☐	☐
**Bias related to confounding factors**						
3	**Were participants included in any comparisons similar?**	Yes, six weight-losing cancer patients	☒	☐	☐	☐
**Bias related to administration of intervention/exposure**						
4	**Were the participants included in any comparisons receiving similar treatment/care, other than the exposure or intervention of interest?**	There is no information on this subject.	☐	☐	☒	☐

	Bias related to assessment, detection and measurement of the outcome		Yes	No	Unclear	N/A
5	**Were there multiple measurements of the outcome, both pre and post the intervention/exposure?**					
	**Outcome 1**	Fasting levels of insulin and glucose at baseline measured by a blood test at day 1 of the study for all study participants	☐	☒	☐	☐

	**Outcome 2**		☐	☐	☐	☐
	**Outcome 3**		☐	☐	☐	☐
	**Outcome 4**		☐	☐	☐	☐
	**Outcome 5**		☐	☐	☐	☐
	**Outcome 6**		☐	☐	☐	☐
	**Outcome 7**		☐	☐	☐	☐

6	**Were the outcomes of participants included in any comparisons measured in the same way?**		**Yes**	**No**	**Unclear**	**N/A**
	**Outcome 1**	Fasting levels of insulin and glucose at baseline measured by a blood test at day 1 of the study for all study participants	☒	☐	☐	☐
	**Outcome 2**		☐	☐	☐	☐
	**Outcome 3**		☐	☐	☐	☐
	**Outcome 4**		☐	☐	☐	☐
	**Outcome 5**		☐	☐	☐	☐
	**Outcome 6**		☐	☐	☐	☐
	**Outcome 7**		☐	☐	☐	☐

7	**Were outcomes measured in a reliable way?**		**Yes**	**No**	**Unclear**	**N/A**

**Outcome 1**	Fasting levels of insulin and glucose at baseline measured by a blood test at day 1 of the study for all study participants	☒	☐	☐	☐
**Outcome 2**		☐	☐	☐	☐
**Outcome 3**		☐	☐	☐	☐
**Outcome 4**		☐	☐	☐	☐
**Outcome 5**		☐	☐	☐	☐
**Outcome 6**		☐	☐	☐	☐
**Outcome 7**		☐	☐	☐	☐

Bias related to participant retention

8	**Was follow-up complete and if not, were differences between groups in terms of their follow-up adequately described and analyzed?**	Follow up not relevant for outcome 1				
	**Outcome 1**		**Yes**	**No**	**Unclear**	**N/A**
	Result 1		☐	☐	☐	☐
	Result 2		☐	☐	☐	☐
	Result 3		☐	☐	☐	☐
	**Outcome 2**		**Yes**	**No**	**Unclear**	**N/A**
	Result 1		☐	☐	☐	☐
	Result 2		☐	☐	☐	☐
	Result 3		☐	☐	☐	☐

Outcome 3		Yes	No	Unclear	N/A
Result 1		☐	☐	☐	☐
Result 2		☐	☐	☐	☐
Result 3		☐	☐	☐	☐
**Outcome 4**		**Yes**	**No**	**Unclear**	**N/A**
Result 1		☐	☐	☐	☐
Result 2		☐	☐	☐	☐
Result 3		☐	☐	☐	☐
**Outcome 5**		**Yes**	**No**	**Unclear**	**N/A**
Result 1		☐	☐	☐	☐
Result 2		☐	☐	☐	☐
Result 3		☐	☐	☐	☐
**Outcome 6**		**Yes**	**No**	**Unclear**	**N/A**
Result 1		☐	☐	☐	☐
Result 2		☐	☐	☐	☐
Result 3		☐	☐	☐	☐
**Outcome 7**		**Yes**	**No**	**Unclear**	**N/A**
Result 1		☐	☐	☐	☐
Result 2		☐	☐	☐	☐
Result 3		☐	☐	☐	☐

Statistical Conclusion Validity

9	**Was appropriate statistical analysis used?**	Statical analysis not relevant for outcome 1				
	**Outcome 1**		**Yes**	**No**	**Unclear**	**N/A**
	Result 1		☐	☐	☐	☐
	Result 2		☐	☐	☐	☐
	Result 3		☐	☐	☐	☐
	**Outcome 2**		**Yes**	**No**	**Unclear**	**N/A**
	Result 1		☐	☐	☐	☐
	Result 2		☐	☐	☐	☐
	Result 3		☐	☐	☐	☐
	**Outcome 3**		**Yes**	**No**	**Unclear**	**N/A**
	Result 1		☐	☐	☐	☐
	Result 2		☐	☐	☐	☐
	Result 3		☐	☐	☐	☐
	**Outcome 4**		**Yes**	**No**	**Unclear**	**N/A**
	Result 1		☐	☐	☐	☐
	Result 2		☐	☐	☐	☐
	Result 3		☐	☐	☐	☐
	**Outcome 5**		**Yes**	**No**	**Unclear**	**N/A**

Result 1		☐	☐	☐	☐
Result 2		☐	☐	☐	☐
Result 3		☐	☐	☐	☐
**Outcome 6**		**Yes**	**No**	**Unclear**	**N/A**
Result 1		☐	☐	☐	☐
Result 2		☐	☐	☐	☐
Result 3		☐	☐	☐	☐
**Outcome 7**		**Yes**	**No**	**Unclear**	**N/A**
Result 1		☐	☐	☐	☐
Result 2		☐	☐	☐	☐
Result 3		☐	☐	☐	☐

Overall appraisal: **Include:** ☒ **Exclude:** ☐ **Seek Further Info:** ☐

Comments:

For our review, we only extract data about fasting insulin and glucose levels at baseline for patients, and this study is appraised according to measurement of these parameters.

S8 JBI checklist

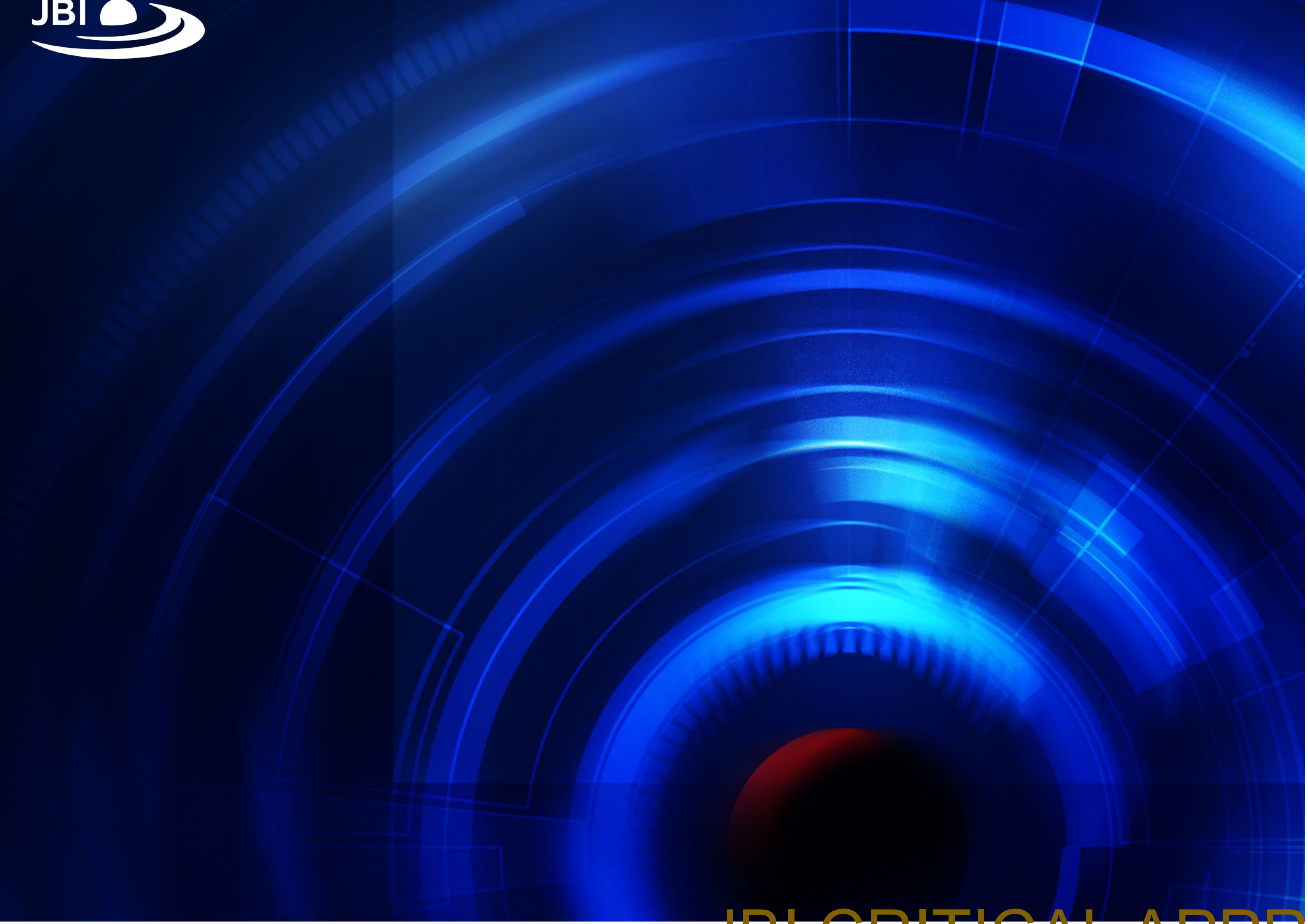

JBI CRITICAL APPRAISAL TOOL

JBI CHECKLIST FOR QUASI-EXPERIMENTAL STUDIES

2023

jbi.global

CRICOS Provider Number 00123M

INTRODUCTION

JBI is a global organization promoting and supporting evidence-based decisions that improve health and health service delivery. JBI offers a unique range of solutions to access, appraise and apply the best available evidence, servicing over 90 countries. Working with 80+ universities, hospitals and NGOs from across the globe through the JBI Collaboration, JBI is a recognized global leader in evidence-based health care.

JBI Systematic Reviews

The core of evidence synthesis is the systematic review of literature of a particular intervention, condition or issue. The systematic review is essentially an analysis of the available evidence and a judgment of the effectiveness or otherwise of a practice, involving a series of complex steps. JBI takes a particular view on what counts as evidence and the methods utilized to synthesize those different types of evidence. In line with this broader view of evidence, JBI has developed theories, methodologies and rigorous processes for the critical appraisal and synthesis of these diverse forms of evidence in order to aid in clinical decision-making in health care. Guidance now exists for conducting reviews of effectiveness research, qualitative research, prevalence/incidence, etiology/risk, economic evaluations, text/opinion, diagnostic test accuracy, mixed-methods, umbrella reviews and scoping reviews. Further information regarding JBI systematic reviews can be found in the JBI Manual for Evidence Synthesis.

JBI Critical Appraisal Tools

All systematic reviews incorporate a process of critique or appraisal of the research evidence. The purpose of this appraisal for quantitative evidence is to determine the extent to which a study has addressed the possibility of bias in its design, conduct and analysis. All papers selected for inclusion in the systematic review (that is – those that meet the inclusion criteria described in the protocol) need to be subjected to rigorous appraisal by two critical appraisers. The results of this appraisal can then be used to inform synthesis and interpretation of the results of the study. Although designed for use in systematic reviews, JBI critical appraisal tools can also be used when creating Critically Appraised Topics, in journal clubs and as an educational tool.

How were these tools developed?

JBI critical appraisal tools have been developed by JBI and collaborators. The particular iteration of this tool was developed by the JBI Effectiveness Methodology Group following oversight by the JBI Scientific Committee.

Like the previous versions of these tools, this version presents signaling questions to prompt reviewers to identify whether certain safeguards of bias have been met, in the primary literature under review. However, unlike previous iterations of this tool, this version has separated questions into whether they provide an answer relating to internal, external or statistical conclusion validity. For questions related to internal validity, these have been further separated to identify what domain of bias they are referring. Finally, this tool has also been structured to facilitate judgments related to bias at different levels (e.g. bias at the outcome level or bias at the result level) where appropriate.

These tools have been approved following extensive peer review by the JBI Scientific Committee.

How to cite

Please use the following when citing this tool: Barker TH, Habibi N, Aromataris E, Stone JC, Leonardi-Bee J, Sears K, et al. The revised JBI critical appraisal tool for the assessment of risk of bias quasi-experimental studies. JBI Evid Synth. 2024;22(3):378-88.

RoB Assessor: A.H. and J.S.	Date of Appraisal: 13.03.25	Record Number: PMID: 2008651
Study Author: Cersosimo et al.	Study Title: The effect of graded doses of insulin peripheral glucose uptake and lactate release in cancer cachexia.	Study Year: 1991

Internal Validity		Choice - Comments/Justification	Yes	No	Unclear	N/A
**Bias related to temporal precedence**						
1	Is it clear in the study what is the "cause" and what is the "effect" (i.e. there is no confusion about which variable comes first)?		☒	☐	☐	☐
**Bias related to selection and allocation**						
2	Was there a control group?		☒	☐	☐	☐
**Bias related to confounding factors**						
3	Were participants included in any comparisons similar?	Yes, five weight-losing cancer patients	☒	☐	☐	☐
**Bias related to administration of intervention/exposure**						
4	Were the participants included in any comparisons receiving similar treatment/care, other than the exposure or intervention of interest?	The study was conducted before the patients underwent surgery	☐	☒	☐	☐

Bias related to assessment, detection and measurement of the outcome						
5	Were there multiple measurements of the outcome, both pre and post the intervention/exposure?		Yes	No	Unclear	N/A

	**Outcome 1**	Fasting levels of insulin and glucose at baseline measured by a blood test at day 1 of the study for all study participants	☐	☒	☐	☐
	**Outcome 2**		☐	☐	☐	☐
	**Outcome 3**		☐	☐	☐	☐
	**Outcome 4**		☐	☐	☐	☐
	**Outcome 5**		☐	☐	☐	☐
	**Outcome 6**		☐	☐	☐	☐
	**Outcome 7**		☐	☐	☐	☐

6	**Were the outcomes of participants included in any comparisons measured in the same way?**		**Yes**	**No**	**Unclear**	**N/A**
	**Outcome 1**	Fasting levels of insulin and glucose at baseline measured by a blood test at day 1 of the study for all study participants	☒	☐	☐	☐
	**Outcome 2**		☐	☐	☐	☐
	**Outcome 3**		☐	☐	☐	☐
	**Outcome 4**		☐	☐	☐	☐
	**Outcome 5**		☐	☐	☐	☐
	**Outcome 6**		☐	☐	☐	☐
	**Outcome 7**		☐	☐	☐	☐

7	Were outcomes measured in a reliable way?		Yes	No	Unclear	N/A
	**Outcome 1**	Fasting levels of insulin and glucose at baseline measured by a blood test at day 1 of the study for all study participants	☒	☐	☐	☐
	**Outcome 2**		☐	☐	☐	☐
	**Outcome 3**		☐	☐	☐	☐
	**Outcome 4**		☐	☐	☐	☐
	**Outcome 5**		☐	☐	☐	☐
	**Outcome 6**		☐	☐	☐	☐
	**Outcome 7**		☐	☐	☐	☐

Bias related to participant retention

8	**Was follow-up complete and if not, were differences between groups in terms of their follow-up adequately described and analyzed?**	Follow up not relevant for outcome 1				
	**Outcome 1**		**Yes**	**No**	**Unclear**	**N/A**
	Result 1		☐	☐	☐	☐
	Result 2		☐	☐	☐	☐
	Result 3		☐	☐	☐	☐
	**Outcome 2**		**Yes**	**No**	**Unclear**	**N/A**
	Result 1		☐	☐	☐	☐
	Result 2		☐	☐	☐	☐

Result 3		☐	☐	☐	☐
**Outcome 3**		**Yes**	**No**	**Unclear**	**N/A**
Result 1		☐	☐	☐	☐
Result 2		☐	☐	☐	☐
Result 3		☐	☐	☐	☐
**Outcome 4**		**Yes**	**No**	**Unclear**	**N/A**
Result 1		☐	☐	☐	☐
Result 2		☐	☐	☐	☐
Result 3		☐	☐	☐	☐
**Outcome 5**		**Yes**	**No**	**Unclear**	**N/A**
Result 1		☐	☐	☐	☐
Result 2		☐	☐	☐	☐
Result 3		☐	☐	☐	☐
**Outcome 6**		**Yes**	**No**	**Unclear**	**N/A**
Result 1		☐	☐	☐	☐
Result 2		☐	☐	☐	☐
Result 3		☐	☐	☐	☐
**Outcome 7**		**Yes**	**No**	**Unclear**	**N/A**
Result 1		☐	☐	☐	☐

Result 2		☐	☐	☐	☐
Result 3		☐	☐	☐	☐

Statistical Conclusion Validity

9	**Was appropriate statistical analysis used?**	Statical analysis not relevant for outcome 1				
	**Outcome 1**		**Yes**	**No**	**Unclear**	**N/A**
	Result 1		☐	☐	☐	☐
	Result 2		☐	☐	☐	☐
	Result 3		☐	☐	☐	☐
	**Outcome 2**		**Yes**	**No**	**Unclear**	**N/A**
	Result 1		☐	☐	☐	☐
	Result 2		☐	☐	☐	☐
	Result 3		☐	☐	☐	☐
	**Outcome 3**		**Yes**	**No**	**Unclear**	**N/A**
	Result 1		☐	☐	☐	☐
	Result 2		☐	☐	☐	☐
	Result 3		☐	☐	☐	☐
	**Outcome 4**		**Yes**	**No**	**Unclear**	**N/A**
	Result 1		☐	☐	☐	☐

Result 2		☐	☐	☐	☐
Result 3		☐	☐	☐	☐
**Outcome 5**		**Yes**	**No**	**Unclear**	**N/A**
Result 1		☐	☐	☐	☐
Result 2		☐	☐	☐	☐
Result 3		☐	☐	☐	☐
**Outcome 6**		**Yes**	**No**	**Unclear**	**N/A**
Result 1		☐	☐	☐	☐
Result 2		☐	☐	☐	☐
Result 3		☐	☐	☐	☐
**Outcome 7**		**Yes**	**No**	**Unclear**	**N/A**
Result 1		☐	☐	☐	☐
Result 2		☐	☐	☐	☐
Result 3		☐	☐	☐	☐

Overall appraisal: **Include:** ☐ **Exclude:** ☐ **Seek Further Info:** ☐

Comments:

For our review, we only extract data about fasting insulin and glucose levels at baseline for patients, and this study is appraised according to measurement of these parameters.

© JBI, 2022. All rights reserved. JBI grants use of these tools for research purposes only. All other enquiries should be sent to jbisynthesis@adelaide.edu.au

S9 JBI checklist

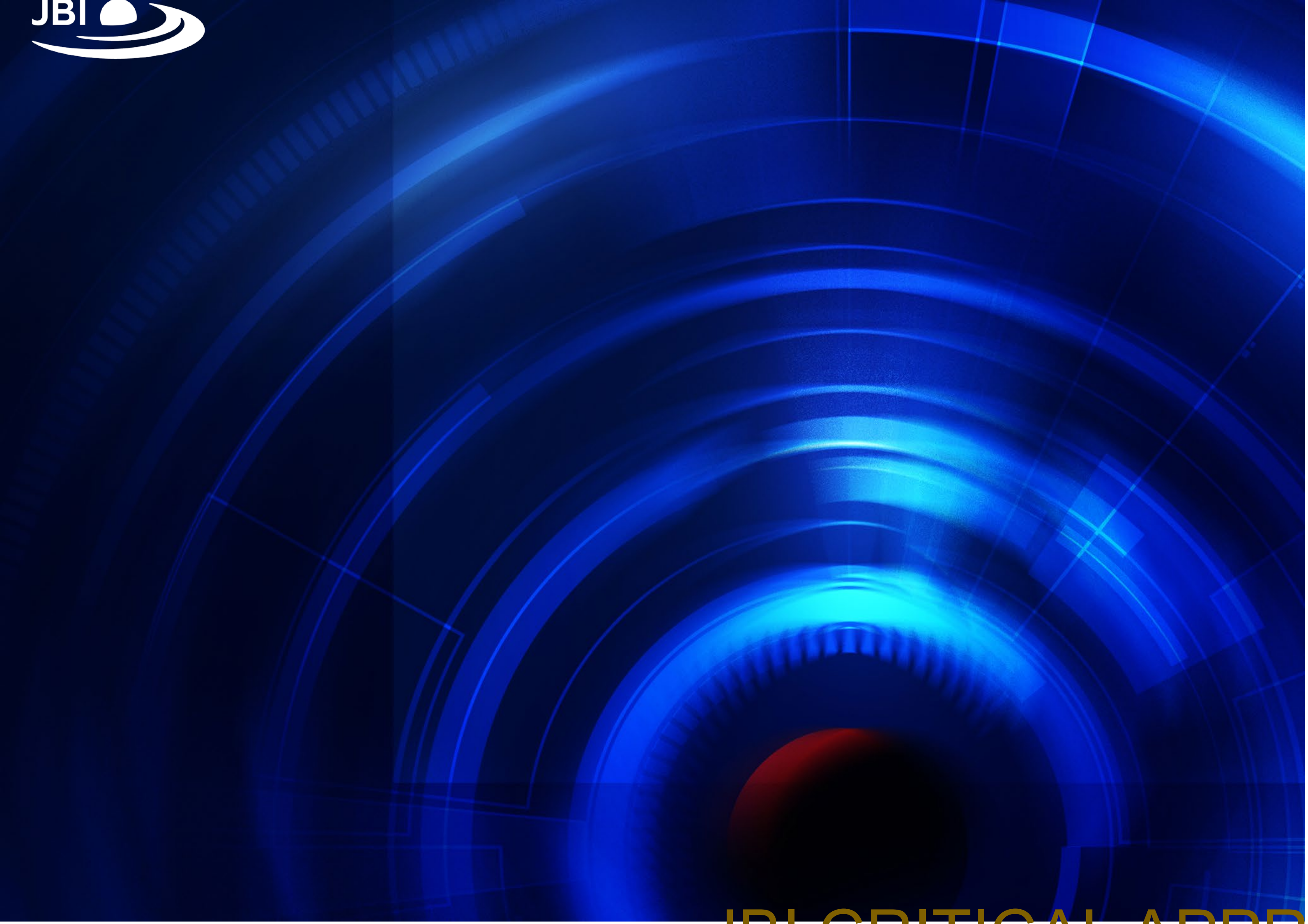

JBI CRITICAL APPRAISAL TOOL

JBI CHECKLIST FOR QUASI-EXPERIMENTAL STUDIES

2023

jbi.global

CRICOS Provider Number 00123M

INTRODUCTION

JBI is a global organization promoting and supporting evidence-based decisions that improve health and health service delivery. JBI offers a unique range of solutions to access, appraise and apply the best available evidence, servicing over 90 countries. Working with 80+ universities, hospitals and NGOs from across the globe through the JBI Collaboration, JBI is a recognized global leader in evidence-based health care.

JBI Systematic Reviews

The core of evidence synthesis is the systematic review of literature of a particular intervention, condition or issue. The systematic review is essentially an analysis of the available evidence and a judgment of the effectiveness or otherwise of a practice, involving a series of complex steps. JBI takes a particular view on what counts as evidence and the methods utilized to synthesize those different types of evidence. In line with this broader view of evidence, JBI has developed theories, methodologies and rigorous processes for the critical appraisal and synthesis of these diverse forms of evidence in order to aid in clinical decision-making in health care. Guidance now exists for conducting reviews of effectiveness research, qualitative research, prevalence/incidence, etiology/risk, economic evaluations, text/opinion, diagnostic test accuracy, mixed-methods, umbrella reviews and scoping reviews. Further information regarding JBI systematic reviews can be found in the JBI Manual for Evidence Synthesis.

JBI Critical Appraisal Tools

All systematic reviews incorporate a process of critique or appraisal of the research evidence. The purpose of this appraisal for quantitative evidence is to determine the extent to which a study has addressed the possibility of bias in its design, conduct and analysis. All papers selected for inclusion in the systematic review (that is – those that meet the inclusion criteria described in the protocol) need to be subjected to rigorous appraisal by two critical appraisers. The results of this appraisal can then be used to inform synthesis and interpretation of the results of the study. Although designed for use in systematic reviews, JBI critical appraisal tools can also be used when creating Critically Appraised Topics, in journal clubs and as an educational tool.

How were these tools developed?

JBI critical appraisal tools have been developed by JBI and collaborators. The particular iteration of this tool was developed by the JBI Effectiveness Methodology Group following oversight by the JBI Scientific Committee.

Like the previous versions of these tools, this version presents signaling questions to prompt reviewers to identify whether certain safeguards of bias have been met, in the primary literature under review. However, unlike previous iterations of this tool, this version has separated questions into whether they provide an answer relating to internal, external or statistical conclusion validity. For questions related to internal validity, these have been further separated to identify what domain of bias they are referring. Finally, this tool has also been structured to facilitate judgments related to bias at different levels (e.g. bias at the outcome level or bias at the result level) where appropriate.

These tools have been approved following extensive peer review by the JBI Scientific Committee.

How to cite

Please use the following when citing this tool: Barker TH, Habibi N, Aromataris E, Stone JC, Leonardi-Bee J, Sears K, et al. The revised JBI critical appraisal tool for the assessment of risk of bias quasi-experimental studies. JBI Evid Synth. 2024;22(3):378-88.

**RoB Assessor: A.H. and J.S.**	**Date of Appraisal: 13.03.25**	**Record Number: PMID: 6367972**
**Study Author: Eden et al.**	**Study Title: Glucose flux in relation to energy expenditure in malnourished patients with and without cancer during periods of fasting and feeding.**	**Study Year: 1984**

Internal Validity		Choice - Comments/Justification	Yes	No	Unclear	N/A
**Bias related to temporal precedence**						
1	**Is it clear in the study what is the "cause" and what is the "effect" (i.e. there is no confusion about which variable comes first)?**		☒	☐	☐	☐
**Bias related to selection and allocation**						
2	**Was there a control group?**		☒	☐	☐	☐
**Bias related to confounding factors**						
3	**Were participants included in any comparisons similar?**	Yes, 8 weight-losing cancer patients	☒	☐	☐	☐
**Bias related to administration of intervention/exposure**						
4	**Were the participants included in any comparisons receiving similar treatment/care, other than the exposure or intervention of interest?**	None of patients had received any cancer treatment before the study.	☐	☒	☐	☐

**Bias related to assessment, detection and measurement of the outcome**						
5	**Were there multiple measurements of the outcome, both pre and post the intervention/exposure?**		**Yes**	**No**	**Unclear**	**N/A**

**Outcome 1**	Fasting levels of insulin and glucose at baseline measured by a blood test at day 1 of the study for all study participants	☐	☒	☐	☐
**Outcome 2**		☐	☐	☐	☐
**Outcome 3**		☐	☐	☐	☐
**Outcome 4**		☐	☐	☐	☐
**Outcome 5**		☐	☐	☐	☐
**Outcome 6**		☐	☐	☐	☐
**Outcome 7**		☐	☐	☐	☐

6	**Were the outcomes of participants included in any comparisons measured in the same way?**		**Yes**	**No**	**Unclear**	**N/A**
	**Outcome 1**	Fasting levels of insulin and glucose at baseline measured by a blood test at day 1 of the study for all study participants	☒	☐	☐	☐
	**Outcome 2**		☐	☐	☐	☐
	**Outcome 3**		☐	☐	☐	☐
	**Outcome 4**		☐	☐	☐	☐
	**Outcome 5**		☐	☐	☐	☐
	**Outcome 6**		☐	☐	☐	☐
	**Outcome 7**		☐	☐	☐	☐

7	Were outcomes measured in a reliable way?		Yes	No	Unclear	N/A
	**Outcome 1**	Fasting levels of insulin and glucose at baseline measured by a blood test at day 1 of the study for all study participants	☒	☐	☐	☐
	**Outcome 2**		☐	☐	☐	☐
	**Outcome 3**		☐	☐	☐	☐
	**Outcome 4**		☐	☐	☐	☐
	**Outcome 5**		☐	☐	☐	☐
	**Outcome 6**		☐	☐	☐	☐
	**Outcome 7**		☐	☐	☐	☐

Bias related to participant retention

8	Was follow-up complete and if not, were differences between groups in terms of their follow-up adequately described and analyzed?	Follow up not relevant for outcome 1				
	**Outcome 1**		**Yes**	**No**	**Unclear**	**N/A**
	Result 1		☐	☐	☐	☐
	Result 2		☐	☐	☐	☐
	Result 3		☐	☐	☐	☐
	**Outcome 2**		**Yes**	**No**	**Unclear**	**N/A**
	Result 1		☐	☐	☐	☐
	Result 2		☐	☐	☐	☐

Result 3		☐	☐	☐	☐
**Outcome 3**		**Yes**	**No**	**Unclear**	**N/A**
Result 1		☐	☐	☐	☐
Result 2		☐	☐	☐	☐
Result 3		☐	☐	☐	☐
**Outcome 4**		**Yes**	**No**	**Unclear**	**N/A**
Result 1		☐	☐	☐	☐
Result 2		☐	☐	☐	☐
Result 3		☐	☐	☐	☐
**Outcome 5**		**Yes**	**No**	**Unclear**	**N/A**
Result 1		☐	☐	☐	☐
Result 2		☐	☐	☐	☐
Result 3		☐	☐	☐	☐
**Outcome 6**		**Yes**	**No**	**Unclear**	**N/A**
Result 1		☐	☐	☐	☐
Result 2		☐	☐	☐	☐
Result 3		☐	☐	☐	☐
**Outcome 7**		**Yes**	**No**	**Unclear**	**N/A**
Result 1		☐	☐	☐	☐
Result 2		☐	☐	☐	☐

	Result 3		☐	☐	☐	☐

Statistical Conclusion Validity

9	**Was appropriate statistical analysis used?**	Statical analysis not relevant for outcome 1				
	**Outcome 1**		**Yes**	**No**	**Unclear**	**N/A**
	Result 1		☐	☐	☐	☐
	Result 2		☐	☐	☐	☐
	Result 3		☐	☐	☐	☐
	**Outcome 2**		**Yes**	**No**	**Unclear**	**N/A**
	Result 1		☐	☐	☐	☐
	Result 2		☐	☐	☐	☐
	Result 3		☐	☐	☐	☐
	**Outcome 3**		**Yes**	**No**	**Unclear**	**N/A**
	Result 1		☐	☐	☐	☐
	Result 2		☐	☐	☐	☐
	Result 3		☐	☐	☐	☐
	**Outcome 4**		**Yes**	**No**	**Unclear**	**N/A**
	Result 1		☐	☐	☐	☐
	Result 2		☐	☐	☐	☐
	Result 3		☐	☐	☐	☐

Outcome 5		Yes	No	Unclear	N/A
Result 1		☐	☐	☐	☐
Result 2		☐	☐	☐	☐
Result 3		☐	☐	☐	☐
**Outcome 6**		**Yes**	**No**	**Unclear**	**N/A**
Result 1		☐	☐	☐	☐
Result 2		☐	☐	☐	☐
Result 3		☐	☐	☐	☐
**Outcome 7**		**Yes**	**No**	**Unclear**	**N/A**
Result 1		☐	☐	☐	☐
Result 2		☐	☐	☐	☐
Result 3		☐	☐	☐	☐

Overall appraisal: **Include:** ☐ **Exclude:** ☐ **Seek Further Info:** ☐

Comments:

For our review, we only extract data about fasting insulin and glucose levels at baseline for patients, and this study is appraised according to measurement of these parameters.

S10 JBI checklist

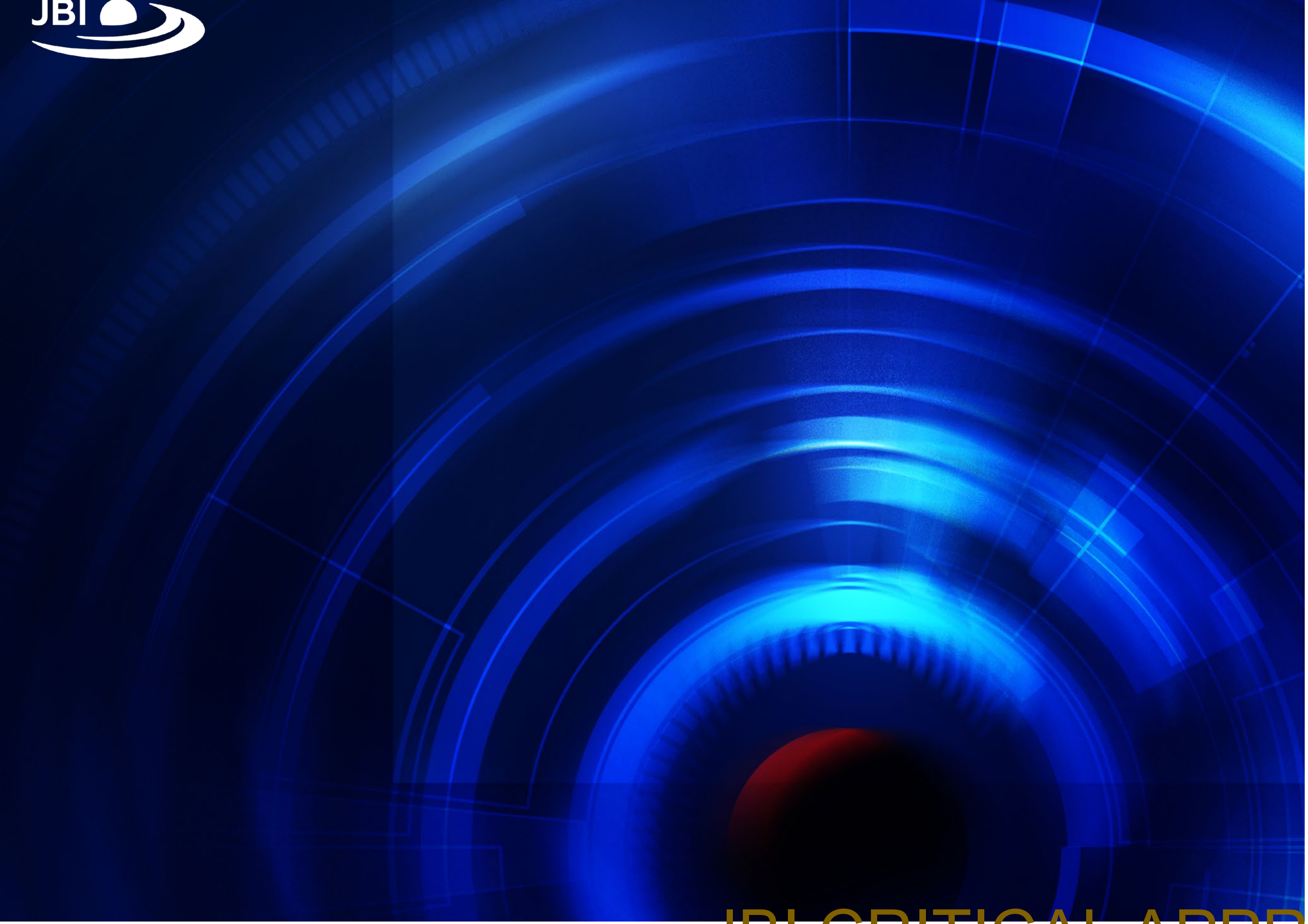

JBI CRITICAL APPRAISAL TOOL

JBI CHECKLIST FOR QUASI-EXPERIMENTAL STUDIES

2023

jbi.global

CRICOS Provider Number 00123M

INTRODUCTION

JBI is a global organization promoting and supporting evidence-based decisions that improve health and health service delivery. JBI offers a unique range of solutions to access, appraise and apply the best available evidence, servicing over 90 countries. Working with 80+ universities, hospitals and NGOs from across the globe through the JBI Collaboration, JBI is a recognized global leader in evidence-based health care.

JBI Systematic Reviews

The core of evidence synthesis is the systematic review of literature of a particular intervention, condition or issue. The systematic review is essentially an analysis of the available evidence and a judgment of the effectiveness or otherwise of a practice, involving a series of complex steps. JBI takes a particular view on what counts as evidence and the methods utilized to synthesize those different types of evidence. In line with this broader view of evidence, JBI has developed theories, methodologies and rigorous processes for the critical appraisal and synthesis of these diverse forms of evidence in order to aid in clinical decision-making in health care. Guidance now exists for conducting reviews of effectiveness research, qualitative research, prevalence/incidence, etiology/risk, economic evaluations, text/opinion, diagnostic test accuracy, mixed-methods, umbrella reviews and scoping reviews. Further information regarding JBI systematic reviews can be found in the JBI Manual for Evidence Synthesis.

JBI Critical Appraisal Tools

All systematic reviews incorporate a process of critique or appraisal of the research evidence. The purpose of this appraisal for quantitative evidence is to determine the extent to which a study has addressed the possibility of bias in its design, conduct and analysis. All papers selected for inclusion in the systematic review (that is – those that meet the inclusion criteria described in the protocol) need to be subjected to rigorous appraisal by two critical appraisers. The results of this appraisal can then be used to inform synthesis and interpretation of the results of the study. Although designed for use in systematic reviews, JBI critical appraisal tools can also be used when creating Critically Appraised Topics, in journal clubs and as an educational tool.

How were these tools developed?

JBI critical appraisal tools have been developed by JBI and collaborators. The particular iteration of this tool was developed by the JBI Effectiveness Methodology Group following oversight by the JBI Scientific Committee.

Like the previous versions of these tools, this version presents signaling questions to prompt reviewers to identify whether certain safeguards of bias have been met, in the primary literature under review. However, unlike previous iterations of this tool, this version has separated questions into whether they provide an answer relating to internal, external or statistical conclusion validity. For questions related to internal validity, these have been further separated to identify what domain of bias they are referring. Finally, this tool has also been structured to facilitate judgments related to bias at different levels (e.g. bias at the outcome level or bias at the result level) where appropriate.

These tools have been approved following extensive peer review by the JBI Scientific Committee.

How to cite

Please use the following when citing this tool: Barker TH, Habibi N, Aromataris E, Stone JC, Leonardi-Bee J, Sears K, et al. The revised JBI critical appraisal tool for the assessment of risk of bias quasi-experimental studies. JBI Evid Synth. 2024;22(3):378-88.

**RoB Assessor: A.H. and J.S.**	**Date of Appraisal: 14.03.25**	**Record Number: doi.org/10.1002/1097-0142(19850101)55:1+<225::aid cncr2820551304>3.0.co;2-7**
**Study Author: Herber D et al.**	**Study Title: Metabolic abnormalities in the cance patient**	**Study Year: 1985**

Internal Validity		Choice - Comments/Justification	Yes	No	Unclear	N/A
**Bias related to temporal precedence**						
1	**Is it clear in the study what is the "cause" and what is the "effect" (i.e. there is no confusion about which variable comes first)?**		☒	☐	☐	☐
**Bias related to selection and allocation**						
2	**Was there a control group?**		☒	☐	☐	☐
**Bias related to confounding factors**						
3	**Were participants included in any comparisons similar?**	Yes, weight-losing cancer patients	☒	☐	☐	☐
**Bias related to administration of intervention/exposure**						
4	**Were the participants included in any comparisons receiving similar treatment/care, other than the exposure or intervention of interest?**	It is unclear if the patients had received any cancer treatment before the study.	☐	☐	☒	☐

**Bias related to assessment, detection and measurement of the outcome**						
5	**Were there multiple measurements of the outcome, both pre and post the intervention/exposure?**		**Yes**	**No**	**Unclear**	**N/A**
	**Outcome 1**	Fasting levels of insulin and glucose at baseline measured by a blood test at day 1 of the study for all study participants	☐	☒	☐	☐

	**Outcome 2**		☐	☐	☐	☐
	**Outcome 3**		☐	☐	☐	☐
	**Outcome 4**		☐	☐	☐	☐
	**Outcome 5**		☐	☐	☐	☐
	**Outcome 6**		☐	☐	☐	☐
	**Outcome 7**		☐	☐	☐	☐

6	**Were the outcomes of participants included in any comparisons measured in the same way?**		**Yes**	**No**	**Unclear**	**N/A**
	**Outcome 1**	Fasting levels of insulin and glucose at baseline measured by a blood test at day 1 of the study for all study participants	☒	☐	☐	☐
	**Outcome 2**		☐	☐	☐	☐
	**Outcome 3**		☐	☐	☐	☐
	**Outcome 4**		☐	☐	☐	☐
	**Outcome 5**		☐	☐	☐	☐
	**Outcome 6**		☐	☐	☐	☐
	**Outcome 7**		☐	☐	☐	☐

7	**Were outcomes measured in a reliable way?**		**Yes**	**No**	**Unclear**	**N/A**

**Outcome 1**	Fasting levels of insulin and glucose at baseline measured by a blood test at day 1 of the study for all study participants	☒	☐	☐	☐
**Outcome 2**		☐	☐	☐	☐
**Outcome 3**		☐	☐	☐	☐
**Outcome 4**		☐	☐	☐	☐
**Outcome 5**		☐	☐	☐	☐
**Outcome 6**		☐	☐	☐	☐
**Outcome 7**		☐	☐	☐	☐

Bias related to participant retention

8	**Was follow-up complete and if not, were differences between groups in terms of their follow-up adequately described and analyzed?**	Follow up not relevant for outcome 1				
	**Outcome 1**		**Yes**	**No**	**Unclear**	**N/A**
	Result 1		☐	☐	☐	☐
	Result 2		☐	☐	☐	☐
	Result 3		☐	☐	☐	☐
	**Outcome 2**		**Yes**	**No**	**Unclear**	**N/A**
	Result 1		☐	☐	☐	☐
	Result 2		☐	☐	☐	☐
	Result 3		☐	☐	☐	☐

		Yes	No	Unclear	N/A
**Outcome 3**		**Yes**	**No**	**Unclear**	**N/A**
Result 1		☐	☐	☐	☐
Result 2		☐	☐	☐	☐
Result 3		☐	☐	☐	☐
**Outcome 4**		**Yes**	**No**	**Unclear**	**N/A**
Result 1		☐	☐	☐	☐
Result 2		☐	☐	☐	☐
Result 3		☐	☐	☐	☐
**Outcome 5**		**Yes**	**No**	**Unclear**	**N/A**
Result 1		☐	☐	☐	☐
Result 2		☐	☐	☐	☐
Result 3		☐	☐	☐	☐
**Outcome 6**		**Yes**	**No**	**Unclear**	**N/A**
Result 1		☐	☐	☐	☐
Result 2		☐	☐	☐	☐
Result 3		☐	☐	☐	☐
**Outcome 7**		**Yes**	**No**	**Unclear**	**N/A**
Result 1		☐	☐	☐	☐
Result 2		☐	☐	☐	☐
Result 3		☐	☐	☐	☐

Statistical Conclusion Validity

9	Was appropriate statistical analysis used?	Statical analysis not relevant for outcome 1				
	**Outcome 1**		**Yes**	**No**	**Unclear**	**N/A**
	Result 1		☐	☐	☐	☐
	Result 2		☐	☐	☐	☐
	Result 3		☐	☐	☐	☐
	**Outcome 2**		**Yes**	**No**	**Unclear**	**N/A**
	Result 1		☐	☐	☐	☐
	Result 2		☐	☐	☐	☐
	Result 3		☐	☐	☐	☐
	**Outcome 3**		**Yes**	**No**	**Unclear**	**N/A**
	Result 1		☐	☐	☐	☐
	Result 2		☐	☐	☐	☐
	Result 3		☐	☐	☐	☐
	**Outcome 4**		**Yes**	**No**	**Unclear**	**N/A**
	Result 1		☐	☐	☐	☐
	Result 2		☐	☐	☐	☐
	Result 3		☐	☐	☐	☐
	**Outcome 5**		**Yes**	**No**	**Unclear**	**N/A**

Result 1		☐	☐	☐	☐
Result 2		☐	☐	☐	☐
Result 3		☐	☐	☐	☐
**Outcome 6**		**Yes**	**No**	**Unclear**	**N/A**
Result 1		☐	☐	☐	☐
Result 2		☐	☐	☐	☐
Result 3		☐	☐	☐	☐
**Outcome 7**		**Yes**	**No**	**Unclear**	**N/A**
Result 1		☐	☐	☐	☐
Result 2		☐	☐	☐	☐
Result 3		☐	☐	☐	☐

Overall appraisal: **Include:** ☐ **Exclude:** ☐ **Seek Further Info:** ☐

Comments:

For our review, we only extract data about fasting insulin and glucose levels at baseline for patients, and this study is appraised according to measurement of these parameters.

S11 JBI checklist

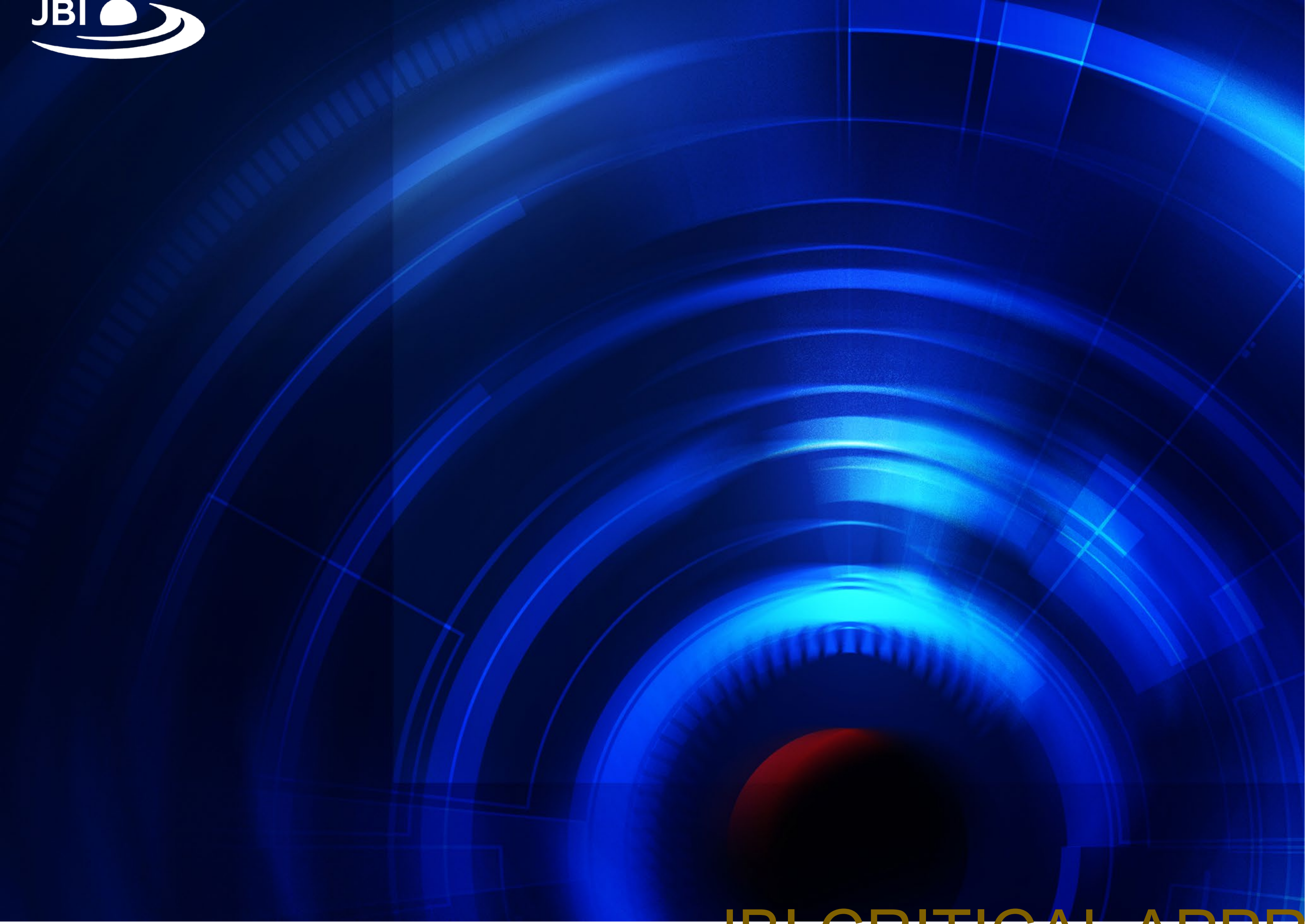

JBI CRITICAL APPRAISAL TOOL

JBI CHECKLIST FOR QUASI-EXPERIMENTAL STUDIES

2023

jbi.global

CRICOS Provider Number 00123M

INTRODUCTION

JBI is a global organization promoting and supporting evidence-based decisions that improve health and health service delivery. JBI offers a unique range of solutions to access, appraise and apply the best available evidence, servicing over 90 countries. Working with 80+ universities, hospitals and NGOs from across the globe through the JBI Collaboration, JBI is a recognized global leader in evidence-based health care.

JBI Systematic Reviews

The core of evidence synthesis is the systematic review of literature of a particular intervention, condition or issue. The systematic review is essentially an analysis of the available evidence and a judgment of the effectiveness or otherwise of a practice, involving a series of complex steps. JBI takes a particular view on what counts as evidence and the methods utilized to synthesize those different types of evidence. In line with this broader view of evidence, JBI has developed theories, methodologies and rigorous processes for the critical appraisal and synthesis of these diverse forms of evidence in order to aid in clinical decision-making in health care. Guidance now exists for conducting reviews of effectiveness research, qualitative research, prevalence/incidence, etiology/risk, economic evaluations, text/opinion, diagnostic test accuracy, mixed-methods, umbrella reviews and scoping reviews. Further information regarding JBI systematic reviews can be found in the JBI Manual for Evidence Synthesis.

JBI Critical Appraisal Tools

All systematic reviews incorporate a process of critique or appraisal of the research evidence. The purpose of this appraisal for quantitative evidence is to determine the extent to which a study has addressed the possibility of bias in its design, conduct and analysis. All papers selected for inclusion in the systematic review (that is – those that meet the inclusion criteria described in the protocol) need to be subjected to rigorous appraisal by two critical appraisers. The results of this appraisal can then be used to inform synthesis and interpretation of the results of the study. Although designed for use in systematic reviews, JBI critical appraisal tools can also be used when creating Critically Appraised Topics, in journal clubs and as an educational tool.

How were these tools developed?

JBI critical appraisal tools have been developed by JBI and collaborators. The particular iteration of this tool was developed by the JBI Effectiveness Methodology Group following oversight by the JBI Scientific Committee.

Like the previous versions of these tools, this version presents signaling questions to prompt reviewers to identify whether certain safeguards of bias have been met, in the primary literature under review. However, unlike previous iterations of this tool, this version has separated questions into whether they provide an answer relating to internal, external or statistical conclusion validity. For questions related to internal validity, these have been further separated to identify what domain of bias they are referring. Finally, this tool has also been structured to facilitate judgments related to bias at different levels (e.g. bias at the outcome level or bias at the result level) where appropriate.

These tools have been approved following extensive peer review by the JBI Scientific Committee.

How to cite

Please use the following when citing this tool: Barker TH, Habibi N, Aromataris E, Stone JC, Leonardi-Bee J, Sears K, et al. The revised JBI critical appraisal tool for the assessment of risk of bias quasi-experimental studies. JBI Evid Synth. 2024;22(3):378-88.

**RoB Assessor: A.H. and J.S.**	**Date of Appraisal: 14.03.25**	**Record Number: PMID: 1617658**
**Study Author: Heslin M et al.**	**Study Title: Effect of systemic hyperinsulinemia cancer patients.**	**Study Year: 1992**

Internal Validity		Choice - Comments/Justification	Yes	No	Unclear	N/A
**Bias related to temporal precedence**						
1	**Is it clear in the study what is the "cause" and what is the "effect" (i.e. there is no confusion about which variable comes first)?**		☒	☐	☐	☐
**Bias related to selection and allocation**						
2	**Was there a control group?**		☒	☐	☐	☐
**Bias related to confounding factors**						
3	**Were participants included in any comparisons similar?**	Yes, cancer patients with weight-loss, and cancer patients without weight-loss	☒	☐	☐	☐
**Bias related to administration of intervention/exposure**						
4	**Were the participants included in any comparisons receiving similar treatment/care, other than the exposure or intervention of interest?**	One patient was treated with chemotherapy before the study, the rest of the patients had not received any prior treatment.	☐	☒	☐	☐

**Bias related to assessment, detection and measurement of the outcome**						
5	**Were there multiple measurements of the outcome, both pre and post the intervention/exposure?**		**Yes**	**No**	**Unclear**	**N/A**

	**Outcome 1**	Fasting levels of insulin and glucose at baseline measured by a blood test at day 1 of the study for all study participants	☐	☒	☐	☐
	**Outcome 2**		☐	☐	☐	☐
	**Outcome 3**		☐	☐	☐	☐
	**Outcome 4**		☐	☐	☐	☐
	**Outcome 5**		☐	☐	☐	☐
	**Outcome 6**		☐	☐	☐	☐
	**Outcome 7**		☐	☐	☐	☐

6	**Were the outcomes of participants included in any comparisons measured in the same way?**		**Yes**	**No**	**Unclear**	**N/A**
	**Outcome 1**	Fasting levels of insulin and glucose at baseline measured by a blood test at day 1 of the study for all study participants	☒	☐	☐	☐
	**Outcome 2**		☐	☐	☐	☐
	**Outcome 3**		☐	☐	☐	☐
	**Outcome 4**		☐	☐	☐	☐
	**Outcome 5**		☐	☐	☐	☐
	**Outcome 6**		☐	☐	☐	☐
	**Outcome 7**		☐	☐	☐	☐

7	Were outcomes measured in a reliable way?		Yes	No	Unclear	N/A
	**Outcome 1**	Fasting levels of insulin and glucose at baseline measured by a blood test at day 1 of the study for all study participants	☒	☐	☐	☐
	**Outcome 2**		☐	☐	☐	☐
	**Outcome 3**		☐	☐	☐	☐
	**Outcome 4**		☐	☐	☐	☐
	**Outcome 5**		☐	☐	☐	☐
	**Outcome 6**		☐	☐	☐	☐
	**Outcome 7**		☐	☐	☐	☐

Bias related to participant retention

8	Was follow-up complete and if not, were differences between groups in terms of their follow-up adequately described and analyzed?	Follow up not relevant for outcome 1				
	**Outcome 1**		**Yes**	**No**	**Unclear**	**N/A**
	Result 1		☐	☐	☐	☐
	Result 2		☐	☐	☐	☐
	Result 3		☐	☐	☐	☐
	**Outcome 2**		**Yes**	**No**	**Unclear**	**N/A**
	Result 1		☐	☐	☐	☐
	Result 2		☐	☐	☐	☐

Result 3		☐	☐	☐	☐
**Outcome 3**		**Yes**	**No**	**Unclear**	**N/A**
Result 1		☐	☐	☐	☐
Result 2		☐	☐	☐	☐
Result 3		☐	☐	☐	☐
**Outcome 4**		**Yes**	**No**	**Unclear**	**N/A**
Result 1		☐	☐	☐	☐
Result 2		☐	☐	☐	☐
Result 3		☐	☐	☐	☐
**Outcome 5**		**Yes**	**No**	**Unclear**	**N/A**
Result 1		☐	☐	☐	☐
Result 2		☐	☐	☐	☐
Result 3		☐	☐	☐	☐
**Outcome 6**		**Yes**	**No**	**Unclear**	**N/A**
Result 1		☐	☐	☐	☐
Result 2		☐	☐	☐	☐
Result 3		☐	☐	☐	☐
**Outcome 7**		**Yes**	**No**	**Unclear**	**N/A**
Result 1		☐	☐	☐	☐

Result 2		☐	☐	☐	☐
Result 3		☐	☐	☐	☐

Statistical Conclusion Validity

9	**Was appropriate statistical analysis used?**	Statical analysis not relevant for outcome 1				
	**Outcome 1**		**Yes**	**No**	**Unclear**	**N/A**
	Result 1		☐	☐	☐	☐
	Result 2		☐	☐	☐	☐
	Result 3		☐	☐	☐	☐
	**Outcome 2**		**Yes**	**No**	**Unclear**	**N/A**
	Result 1		☐	☐	☐	☐
	Result 2		☐	☐	☐	☐
	Result 3		☐	☐	☐	☐
	**Outcome 3**		**Yes**	**No**	**Unclear**	**N/A**
	Result 1		☐	☐	☐	☐
	Result 2		☐	☐	☐	☐
	Result 3		☐	☐	☐	☐
	**Outcome 4**		**Yes**	**No**	**Unclear**	**N/A**
	Result 1		☐	☐	☐	☐

Result 2		☐	☐	☐	☐
Result 3		☐	☐	☐	☐
**Outcome 5**		**Yes**	**No**	**Unclear**	**N/A**
Result 1		☐	☐	☐	☐
Result 2		☐	☐	☐	☐
Result 3		☐	☐	☐	☐
**Outcome 6**		**Yes**	**No**	**Unclear**	**N/A**
Result 1		☐	☐	☐	☐
Result 2		☐	☐	☐	☐
Result 3		☐	☐	☐	☐
**Outcome 7**		**Yes**	**No**	**Unclear**	**N/A**
Result 1		☐	☐	☐	☐
Result 2		☐	☐	☐	☐
Result 3		☐	☐	☐	☐

Overall appraisal: **Include:** ☐ **Exclude:** ☐ **Seek Further Info:** ☐

Comments:

For our review, we only extract data about fasting insulin and glucose levels at baseline for patients with weight loss and patients with no weight loss, and this study is appraised according to measurement of these parameters.

© JBI, 2022. All rights reserved. JBI grants use of these tools for research purposes only. All other enquiries should be sent to jbisynthesis@adelaide.edu.au

S12 JBI checklist

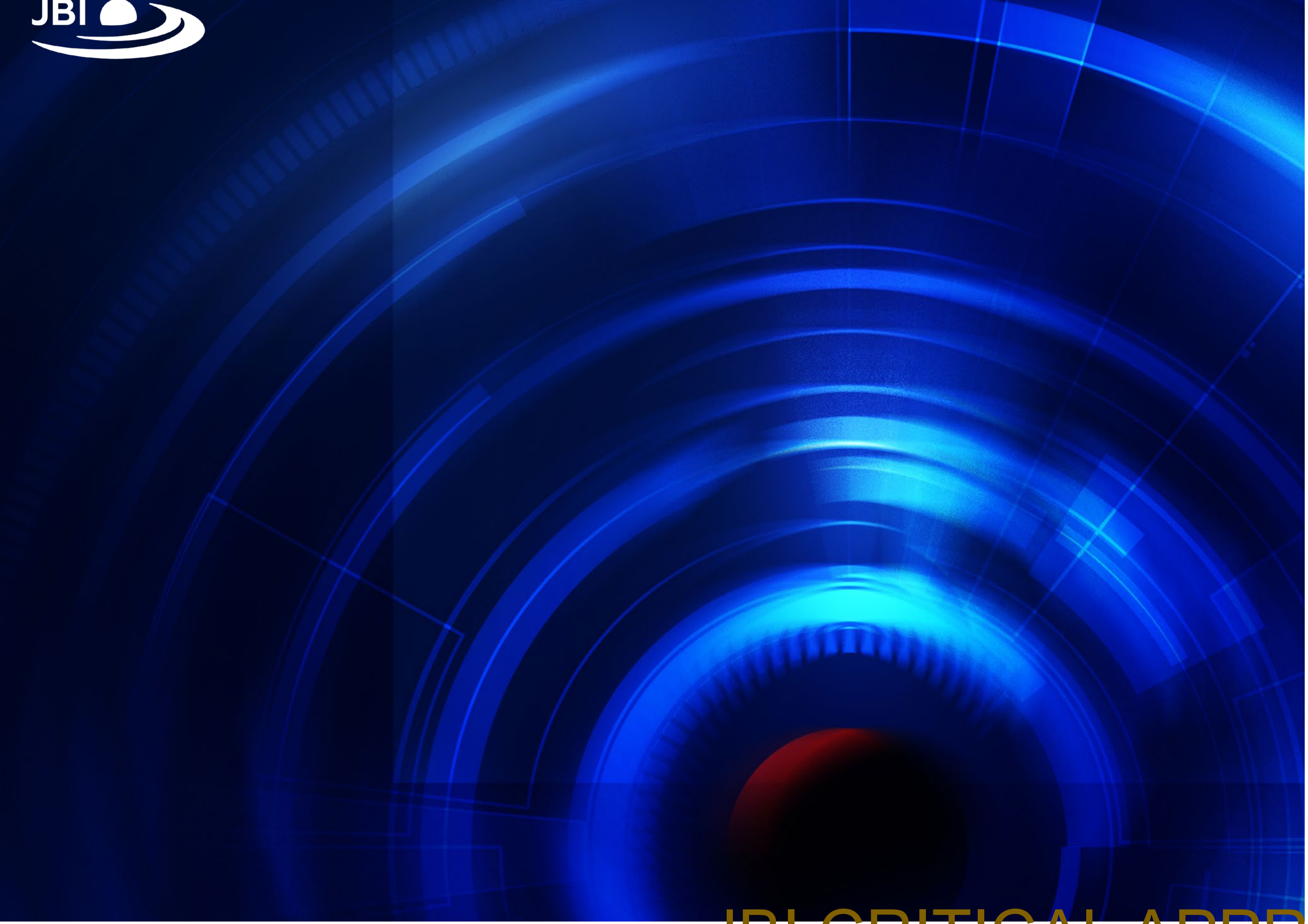

JBI CRITICAL APPRAISAL TOOL

JBI CHECKLIST FOR QUASI-EXPERIMENTAL STUDIES

2023

jbi.global

CRICOS Provider Number 00123M

INTRODUCTION

JBI is a global organization promoting and supporting evidence-based decisions that improve health and health service delivery. JBI offers a unique range of solutions to access, appraise and apply the best available evidence, servicing over 90 countries. Working with 80+ universities, hospitals and NGOs from across the globe through the JBI Collaboration, JBI is a recognized global leader in evidence-based health care.

JBI Systematic Reviews

The core of evidence synthesis is the systematic review of literature of a particular intervention, condition or issue. The systematic review is essentially an analysis of the available evidence and a judgment of the effectiveness or otherwise of a practice, involving a series of complex steps. JBI takes a particular view on what counts as evidence and the methods utilized to synthesize those different types of evidence. In line with this broader view of evidence, JBI has developed theories, methodologies and rigorous processes for the critical appraisal and synthesis of these diverse forms of evidence in order to aid in clinical decision-making in health care. Guidance now exists for conducting reviews of effectiveness research, qualitative research, prevalence/incidence, etiology/risk, economic evaluations, text/opinion, diagnostic test accuracy, mixed-methods, umbrella reviews and scoping reviews. Further information regarding JBI systematic reviews can be found in the JBI Manual for Evidence Synthesis.

JBI Critical Appraisal Tools

All systematic reviews incorporate a process of critique or appraisal of the research evidence. The purpose of this appraisal for quantitative evidence is to determine the extent to which a study has addressed the possibility of bias in its design, conduct and analysis. All papers selected for inclusion in the systematic review (that is – those that meet the inclusion criteria described in the protocol) need to be subjected to rigorous appraisal by two critical appraisers. The results of this appraisal can then be used to inform synthesis and interpretation of the results of the study. Although designed for use in systematic reviews, JBI critical appraisal tools can also be used when creating Critically Appraised Topics, in journal clubs and as an educational tool.

How were these tools developed?

JBI critical appraisal tools have been developed by JBI and collaborators. The particular iteration of this tool was developed by the JBI Effectiveness Methodology Group following oversight by the JBI Scientific Committee.

Like the previous versions of these tools, this version presents signaling questions to prompt reviewers to identify whether certain safeguards of bias have been met, in the primary literature under review. However, unlike previous iterations of this tool, this version has separated questions into whether they provide an answer relating to internal, external or statistical conclusion validity. For questions related to internal validity, these have been further separated to identify what domain of bias they are referring. Finally, this tool has also been structured to facilitate judgments related to bias at different levels (e.g. bias at the outcome level or bias at the result level) where appropriate.

These tools have been approved following extensive peer review by the JBI Scientific Committee.

How to cite

Please use the following when citing this tool: Barker TH, Habibi N, Aromataris E, Stone JC, Leonardi-Bee J, Sears K, et al. The revised JBI critical appraisal tool for the assessment of risk of bias quasi-experimental studies. JBI Evid Synth. 2024;22(3):378-88.

RoB Assessor: A.H. and J.S.	Date of Appraisal: 14.03.25	Record Number: PMID: 6388829
Study Author: Holroyde C et al.	Study Title: Glucose metabolism in cachectic patients with colorectal cancer	Study Year: 1984

	Internal Validity	Choice - Comments/Justification	Yes	No	Unclear	N/A
**Bias related to temporal precedence**						
1	**Is it clear in the study what is the "cause" and what is the "effect" (i.e. there is no confusion about which variable comes first)?**		☒	☐	☐	☐
**Bias related to selection and allocation**						
2	**Was there a control group?**		☒	☐	☐	☐
**Bias related to confounding factors**						
3	**Were participants included in any comparisons similar?**	Yes, weight-losing colorectal cancer patients	☒	☐	☐	☐
**Bias related to administration of intervention/exposure**						
4	**Were the participants included in any comparisons receiving similar treatment/care, other than the exposure or intervention of interest?**	All patients were previously treated with chemotherapy, but did not receive treatment during the study	☐	☒	☐	☐

	Bias related to assessment, detection and measurement of the outcome		Yes	No	Unclear	N/A
5	**Were there multiple measurements of the outcome, both pre and post the intervention/exposure?**					
	**Outcome 1**	Fasting levels of insulin and glucose at baseline measured by a blood test at day 1 of the study for all study participants	☐	☒	☐	☐

	**Outcome 2**		☐	☐	☐	☐
	**Outcome 3**		☐	☐	☐	☐
	**Outcome 4**		☐	☐	☐	☐
	**Outcome 5**		☐	☐	☐	☐
	**Outcome 6**		☐	☐	☐	☐
	**Outcome 7**		☐	☐	☐	☐

6	**Were the outcomes of participants included in any comparisons measured in the same way?**		**Yes**	**No**	**Unclear**	**N/A**
	**Outcome 1**	Fasting levels of insulin and glucose at baseline measured by a blood test at day 1 of the study for all study participants	☒	☐	☐	☐
	**Outcome 2**		☐	☐	☐	☐
	**Outcome 3**		☐	☐	☐	☐
	**Outcome 4**		☐	☐	☐	☐
	**Outcome 5**		☐	☐	☐	☐
	**Outcome 6**		☐	☐	☐	☐
	**Outcome 7**		☐	☐	☐	☐

7	**Were outcomes measured in a reliable way?**		**Yes**	**No**	**Unclear**	**N/A**

Outcome 1	Fasting levels of insulin and glucose at baseline measured by a blood test at day 1 of the study for all study participants	☒	☐	☐	☐
Outcome 2		☐	☐	☐	☐
Outcome 3		☐	☐	☐	☐
Outcome 4		☐	☐	☐	☐
Outcome 5		☐	☐	☐	☐
Outcome 6		☐	☐	☐	☐
Outcome 7		☐	☐	☐	☐

Bias related to participant retention

8	**Was follow-up complete and if not, were differences between groups in terms of their follow-up adequately described and analyzed?**	Follow up not relevant for outcome 1				
	**Outcome 1**		**Yes**	**No**	**Unclear**	**N/A**
	Result 1		☐	☐	☐	☐
	Result 2		☐	☐	☐	☐
	Result 3		☐	☐	☐	☐
	**Outcome 2**		**Yes**	**No**	**Unclear**	**N/A**
	Result 1		☐	☐	☐	☐
	Result 2		☐	☐	☐	☐
	Result 3		☐	☐	☐	☐

Outcome 3		Yes	No	Unclear	N/A
Result 1		☐	☐	☐	☐
Result 2		☐	☐	☐	☐
Result 3		☐	☐	☐	☐
**Outcome 4**		**Yes**	**No**	**Unclear**	**N/A**
Result 1		☐	☐	☐	☐
Result 2		☐	☐	☐	☐
Result 3		☐	☐	☐	☐
**Outcome 5**		**Yes**	**No**	**Unclear**	**N/A**
Result 1		☐	☐	☐	☐
Result 2		☐	☐	☐	☐
Result 3		☐	☐	☐	☐
**Outcome 6**		**Yes**	**No**	**Unclear**	**N/A**
Result 1		☐	☐	☐	☐
Result 2		☐	☐	☐	☐
Result 3		☐	☐	☐	☐
**Outcome 7**		**Yes**	**No**	**Unclear**	**N/A**
Result 1		☐	☐	☐	☐
Result 2		☐	☐	☐	☐
Result 3		☐	☐	☐	☐

Statistical Conclusion Validity

9	Was appropriate statistical analysis used?	Statical analysis not relevant for outcome 1				
	**Outcome 1**		**Yes**	**No**	**Unclear**	**N/A**
	Result 1		☐	☐	☐	☐
	Result 2		☐	☐	☐	☐
	Result 3		☐	☐	☐	☐
	**Outcome 2**		**Yes**	**No**	**Unclear**	**N/A**
	Result 1		☐	☐	☐	☐
	Result 2		☐	☐	☐	☐
	Result 3		☐	☐	☐	☐
	**Outcome 3**		**Yes**	**No**	**Unclear**	**N/A**
	Result 1		☐	☐	☐	☐
	Result 2		☐	☐	☐	☐
	Result 3		☐	☐	☐	☐
	**Outcome 4**		**Yes**	**No**	**Unclear**	**N/A**
	Result 1		☐	☐	☐	☐
	Result 2		☐	☐	☐	☐
	Result 3		☐	☐	☐	☐
	**Outcome 5**		**Yes**	**No**	**Unclear**	**N/A**

Result 1		☐	☐	☐	☐
Result 2		☐	☐	☐	☐
Result 3		☐	☐	☐	☐
**Outcome 6**		**Yes**	**No**	**Unclear**	**N/A**
Result 1		☐	☐	☐	☐
Result 2		☐	☐	☐	☐
Result 3		☐	☐	☐	☐
**Outcome 7**		**Yes**	**No**	**Unclear**	**N/A**
Result 1		☐	☐	☐	☐
Result 2		☐	☐	☐	☐
Result 3		☐	☐	☐	☐

Overall appraisal: **Include:** ☐ **Exclude:** ☐ **Seek Further Info:** ☐

Comments:

For our review, we only extract data about fasting insulin and glucose levels at baseline for patients, and this study is appraised according to measurement of these parameters.

S13 JBI checklist

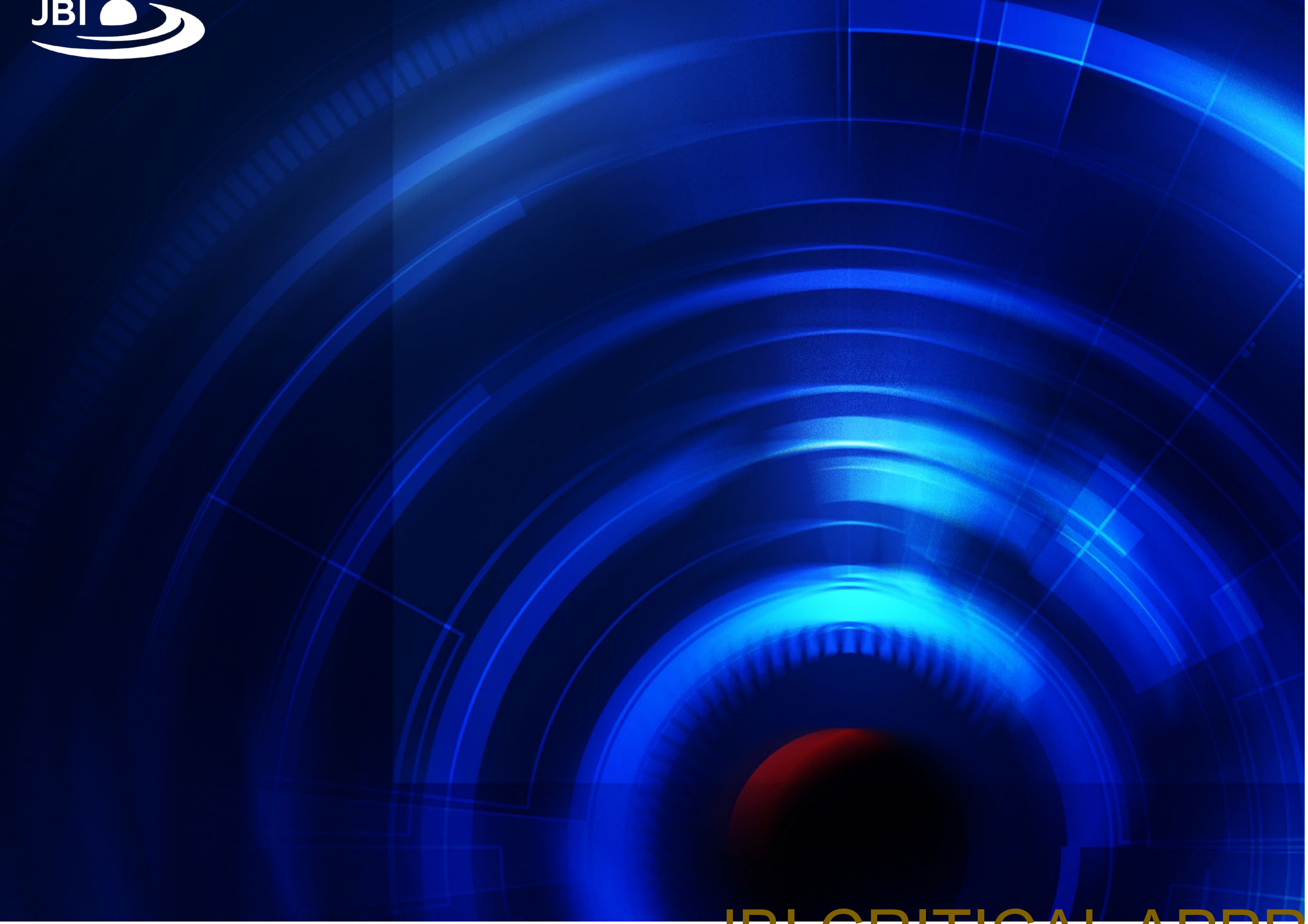

JBI CRITICAL APPRAISAL TOOL

JBI CHECKLIST FOR QUASI-EXPERIMENTAL STUDIES

2023

jbi.global

CRICOS Provider Number 00123M

INTRODUCTION

JBI is a global organization promoting and supporting evidence-based decisions that improve
health and health service delivery. JBI offers a unique range of solutions to access, appraise and apply the best available evidence, servicing over 90 countries. Working with 80+ universities, hospitals and NGOs from across the globe through the JBI Collaboration, JBI is a recognized global leader in evidence-based health care.

JBI Systematic Reviews

The core of evidence synthesis is the systematic review of literature of a particular intervention, condition or issue. The systematic review is essentially an analysis of the available evidence and a judgment of the effectiveness or otherwise of a practice, involving a series of complex steps. JBI takes a particular view on what counts as evidence and the methods utilized to synthesize those different types of evidence. In line with this broader view of evidence, JBI has developed theories, methodologies and rigorous processes for the critical appraisal and synthesis of these diverse forms of evidence in order to aid in clinical decision-making in health care. Guidance now exists for conducting reviews of effectiveness research, qualitative research, prevalence/incidence, etiology/risk, economic evaluations, text/opinion, diagnostic test accuracy, mixed-methods, umbrella reviews and scoping reviews. Further information regarding JBI systematic reviews can be found in the JBI Manual for Evidence Synthesis.

JBI Critical Appraisal Tools

All systematic reviews incorporate a process of critique or appraisal of the research evidence. The purpose of this appraisal for quantitative evidence is to determine the extent to which a study has addressed the possibility of bias in its design, conduct and analysis. All papers selected for inclusion in the systematic review (that is – those that meet the inclusion criteria described in the protocol) need to be subjected to rigorous appraisal by two critical appraisers. The results of this appraisal can then be used to inform synthesis and interpretation of the results of the study. Although designed for use in systematic reviews, JBI critical appraisal tools can also be used when creating Critically Appraised Topics, in journal clubs and as an educational tool.

How were these tools developed?

JBI critical appraisal tools have been developed by JBI and collaborators. The particular iteration of this tool was developed by the JBI Effectiveness Methodology Group following oversight by the JBI Scientific Committee.

Like the previous versions of these tools, this version presents signaling questions to prompt reviewers to identify whether certain safeguards of bias have been met, in the primary literature under review. However, unlike previous iterations of this tool, this version has separated questions into whether they provide an answer relating to internal, external or statistical conclusion validity. For questions related to internal validity, these have been further separated to identify what domain of bias they are referring. Finally, this tool has also been structured to facilitate judgments related to bias at different levels (e.g. bias at the outcome level or bias at the result level) where appropriate.

These tools have been approved following extensive peer review by the JBI Scientific Committee.

How to cite

Please use the following when citing this tool: Barker TH, Habibi N, Aromataris E, Stone JC, Leonardi-Bee J, Sears K, et al. The revised JBI critical appraisal tool for the assessment of risk of bias quasi-experimental studies. JBI Evid Synth. 2024;22(3):378-88.

**RoB Assessor: A.H. and J.S.**	**Date of Appraisal: 14.03.25**	**Record Number: PMID: 10676645**
**Study Author: Leij-Halfwerk S et al.**	**Study Title: Altered hepatic gluconeogenesis dur L-alanine infusion in weight-losing lung cancer patients as observed by phosphorus magnetic resonance spectroscopy and turnover measureme**	**Study Year: 2000**

	Internal Validity	Choice - Comments/Justification	Yes	No	Unclear	N/A
	**Bias related to temporal precedence**					
1	**Is it clear in the study what is the "cause" and what is the "effect" (i.e. there is no confusion about which variable comes first)?**		☒	☐	☐	☐
	**Bias related to selection and allocation**					
2	**Was there a control group?**		☒	☐	☐	☐
	**Bias related to confounding factors**					
3	**Were participants included in any comparisons similar?**	Yes, cancer patients with weight-loss, and cancer patients without weight-loss	☒	☐	☐	☐
	**Bias related to administration of intervention/exposure**					
4	**Were the participants included in any comparisons receiving similar treatment/care, other than the exposure or intervention of interest?**	The study includes both previously treated and untreated patients, but none of the patients received treatment during the study	☒	☒	☐	☐

	Bias related to assessment, detection and measurement of the outcome		Yes	No	Unclear	N/A
5	**Were there multiple measurements of the outcome, both pre and post the intervention/exposure?**					

	Outcome 1	Fasting levels of insulin and glucose at baseline measured by a blood test at day 1 of the study for all study participants	☐	☒	☐	☐
	Outcome 2		☐	☐	☐	☐
	Outcome 3		☐	☐	☐	☐
	Outcome 4		☐	☐	☐	☐
	Outcome 5		☐	☐	☐	☐
	Outcome 6		☐	☐	☐	☐
	Outcome 7		☐	☐	☐	☐

6	**Were the outcomes of participants included in any comparisons measured in the same way?**		**Yes**	**No**	**Unclear**	**N/A**
	**Outcome 1**	Fasting levels of insulin and glucose at baseline measured by a blood test at day 1 of the study for all study participants	☒	☐	☐	☐
	**Outcome 2**		☐	☐	☐	☐
	**Outcome 3**		☐	☐	☐	☐
	**Outcome 4**		☐	☐	☐	☐
	**Outcome 5**		☐	☐	☐	☐
	**Outcome 6**		☐	☐	☐	☐
	**Outcome 7**		☐	☐	☐	☐

7	Were outcomes measured in a reliable way?		Yes	No	Unclear	N/A
	**Outcome 1**	Fasting levels of insulin and glucose at baseline measured by a blood test at day 1 of the study for all study participants	☒	☐	☐	☐
	**Outcome 2**		☐	☐	☐	☐
	**Outcome 3**		☐	☐	☐	☐
	**Outcome 4**		☐	☐	☐	☐
	**Outcome 5**		☐	☐	☐	☐
	**Outcome 6**		☐	☐	☐	☐
	**Outcome 7**		☐	☐	☐	☐

Bias related to participant retention

8	Was follow-up complete and if not, were differences between groups in terms of their follow-up adequately described and analyzed?	Follow up not relevant for outcome 1				
	**Outcome 1**		Yes	No	Unclear	N/A
	Result 1		☐	☐	☐	☐
	Result 2		☐	☐	☐	☐
	Result 3		☐	☐	☐	☐
	**Outcome 2**		Yes	No	Unclear	N/A
	Result 1		☐	☐	☐	☐
	Result 2		☐	☐	☐	☐

Result 3		☐	☐	☐	☐
**Outcome 3**		**Yes**	**No**	**Unclear**	**N/A**
Result 1		☐	☐	☐	☐
Result 2		☐	☐	☐	☐
Result 3		☐	☐	☐	☐
**Outcome 4**		**Yes**	**No**	**Unclear**	**N/A**
Result 1		☐	☐	☐	☐
Result 2		☐	☐	☐	☐
Result 3		☐	☐	☐	☐
**Outcome 5**		**Yes**	**No**	**Unclear**	**N/A**
Result 1		☐	☐	☐	☐
Result 2		☐	☐	☐	☐
Result 3		☐	☐	☐	☐
**Outcome 6**		**Yes**	**No**	**Unclear**	**N/A**
Result 1		☐	☐	☐	☐
Result 2		☐	☐	☐	☐
Result 3		☐	☐	☐	☐
**Outcome 7**		**Yes**	**No**	**Unclear**	**N/A**
Result 1		☐	☐	☐	☐
Result 2		☐	☐	☐	☐

Result 3		☐	☐	☐	☐

Statistical Conclusion Validity

9	**Was appropriate statistical analysis used?**	Statical analysis not relevant for outcome 1				
	**Outcome 1**		**Yes**	**No**	**Unclear**	**N/A**
	Result 1		☐	☐	☐	☐
	Result 2		☐	☐	☐	☐
	Result 3		☐	☐	☐	☐
	**Outcome 2**		**Yes**	**No**	**Unclear**	**N/A**
	Result 1		☐	☐	☐	☐
	Result 2		☐	☐	☐	☐
	Result 3		☐	☐	☐	☐
	**Outcome 3**		**Yes**	**No**	**Unclear**	**N/A**
	Result 1		☐	☐	☐	☐
	Result 2		☐	☐	☐	☐
	Result 3		☐	☐	☐	☐
	**Outcome 4**		**Yes**	**No**	**Unclear**	**N/A**
	Result 1		☐	☐	☐	☐
	Result 2		☐	☐	☐	☐
	Result 3		☐	☐	☐	☐

**Outcome 5**		**Yes**	**No**	**Unclear**	**N/A**
Result 1		☐	☐	☐	☐
Result 2		☐	☐	☐	☐
Result 3		☐	☐	☐	☐
**Outcome 6**		**Yes**	**No**	**Unclear**	**N/A**
Result 1		☐	☐	☐	☐
Result 2		☐	☐	☐	☐
Result 3		☐	☐	☐	☐
**Outcome 7**		**Yes**	**No**	**Unclear**	**N/A**
Result 1		☐	☐	☐	☐
Result 2		☐	☐	☐	☐
Result 3		☐	☐	☐	☐

Overall appraisal: **Include:** ☐ **Exclude:** ☐ **Seek Further Info:** ☐

Comments:

For our review, we only extract data about fasting insulin and glucose levels at baseline for patients with weight loss and patients with no weight loss, and this study is appraised according to measurement of these parameters.

S14 JBI checklist

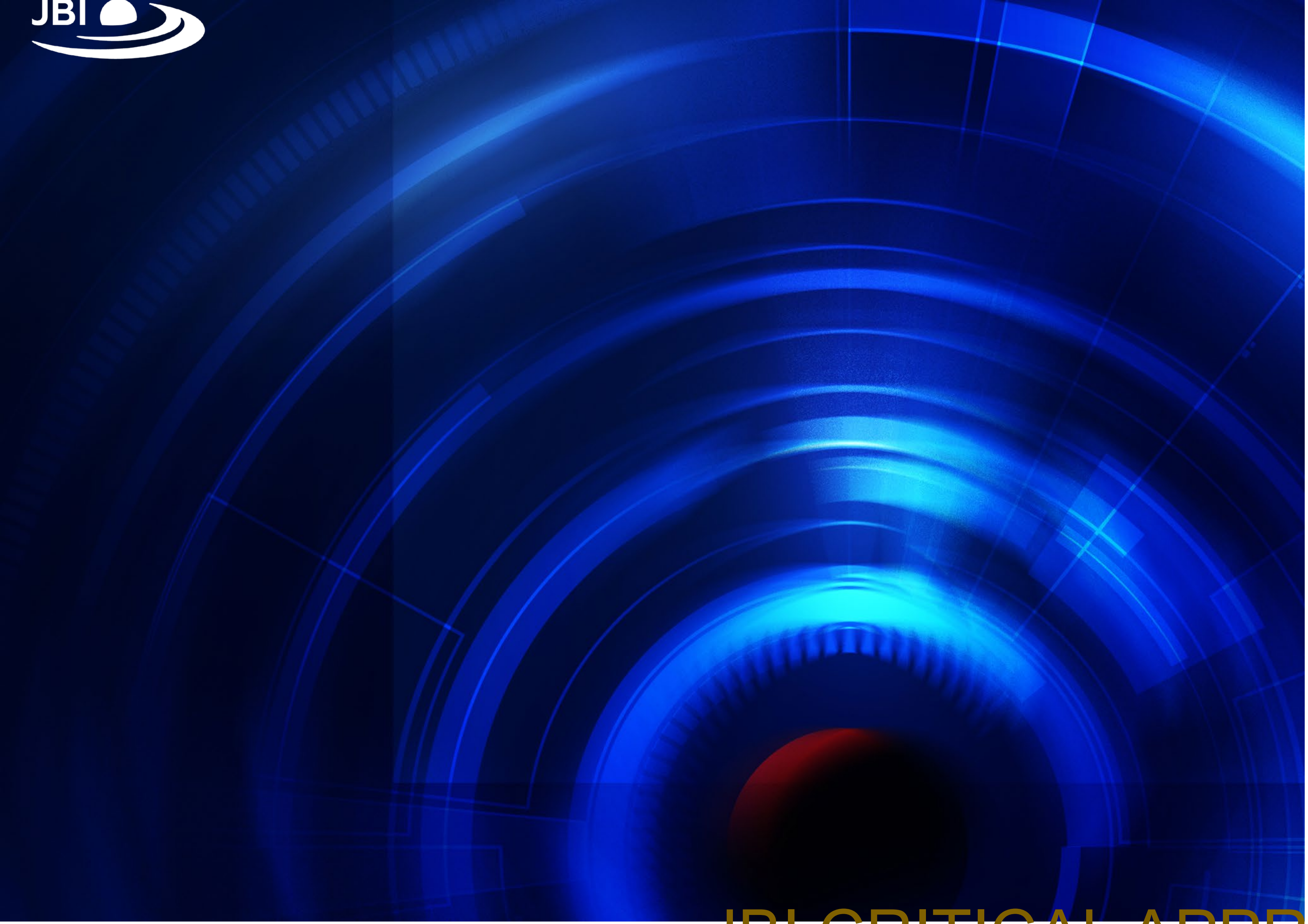

JBI CRITICAL APPRAISAL TOOL

JBI CHECKLIST FOR QUASI-EXPERIMENTAL STUDIES

2023

jbi.global

CRICOS Provider Number 00123M

INTRODUCTION

JBI is a global organization promoting and supporting evidence-based decisions that improve health and health service delivery. JBI offers a unique range of solutions to access, appraise and apply the best available evidence, servicing over 90 countries. Working with 80+ universities, hospitals and NGOs from across the globe through the JBI Collaboration, JBI is a recognized global leader in evidence-based health care.

JBI Systematic Reviews

The core of evidence synthesis is the systematic review of literature of a particular intervention, condition or issue. The systematic review is essentially an analysis of the available evidence and a judgment of the effectiveness or otherwise of a practice, involving a series of complex steps. JBI takes a particular view on what counts as evidence and the methods utilized to synthesize those different types of evidence. In line with this broader view of evidence, JBI has developed theories, methodologies and rigorous processes for the critical appraisal and synthesis of these diverse forms of evidence in order to aid in clinical decision-making in health care. Guidance now exists for conducting reviews of effectiveness research, qualitative research, prevalence/incidence, etiology/risk, economic evaluations, text/opinion, diagnostic test accuracy, mixed-methods, umbrella reviews and scoping reviews. Further information regarding JBI systematic reviews can be found in the JBI Manual for Evidence Synthesis.

JBI Critical Appraisal Tools

All systematic reviews incorporate a process of critique or appraisal of the research evidence. The purpose of this appraisal for quantitative evidence is to determine the extent to which a study has addressed the possibility of bias in its design, conduct and analysis. All papers selected for inclusion in the systematic review (that is – those that meet the inclusion criteria described in the protocol) need to be subjected to rigorous appraisal by two critical appraisers. The results of this appraisal can then be used to inform synthesis and interpretation of the results of the study. Although designed for use in systematic reviews, JBI critical appraisal tools can also be used when creating Critically Appraised Topics, in journal clubs and as an educational tool.

How were these tools developed?

JBI critical appraisal tools have been developed by JBI and collaborators. The particular iteration of this tool was developed by the JBI Effectiveness Methodology Group following oversight by the JBI Scientific Committee.

Like the previous versions of these tools, this version presents signaling questions to prompt reviewers to identify whether certain safeguards of bias have been met, in the primary literature under review. However, unlike previous iterations of this tool, this version has separated questions into whether they provide an answer relating to internal, external or statistical conclusion validity. For questions related to internal validity, these have been further separated to identify what domain of bias they are referring. Finally, this tool has also been structured to facilitate judgments related to bias at different levels (e.g. bias at the outcome level or bias at the result level) where appropriate.

These tools have been approved following extensive peer review by the JBI Scientific Committee.

How to cite

Please use the following when citing this tool: Barker TH, Habibi N, Aromataris E, Stone JC, Leonardi-Bee J, Sears K, et al. The revised JBI critical appraisal tool for the assessment of risk of bias quasi-experimental studies. JBI Evid Synth. 2024;22(3):378-88.

RoB Assessor: A.H. and J.S.	Date of Appraisal: 14.03.25	Record Number: doi.org/10.1002/bjs.1800791240
Study Author: McCall J. et al.	Study Title: Serum tumour necrosis factor alpha and insulin resistance in gastrointestinal cancer	Study Year: 1992

Internal Validity		Choice - Comments/Justification	Yes	No	Unclear	N/A
**Bias related to temporal precedence**						
1	Is it clear in the study what is the "cause" and what is the "effect" (i.e. there is no confusion about which variable comes first)?		☒	☐	☐	☐
**Bias related to selection and allocation**						
2	Was there a control group?		☒	☐	☐	☐
**Bias related to confounding factors**						
3	Were participants included in any comparisons similar?	Yes, cancer patients with weight-loss, and cancer patients without weight-loss	☒	☐	☐	☐
**Bias related to administration of intervention/exposure**						
4	Were the participants included in any comparisons receiving similar treatment/care, other than the exposure or intervention of interest?	Patients were previously treated, but did not receive treatment during the study.	☐	☒	☐	☐

**Bias related to assessment, detection and measurement of the outcome**			Yes	No	Unclear	N/A
5	Were there multiple measurements of the outcome, both pre and post the intervention/exposure?					
	Outcome 1	Fasting levels of insulin and glucose at baseline measured by a blood test at day 1 of the study for all study participants	☐	☒	☐	☐

	**Outcome 2**		☐	☐	☐	☐
	**Outcome 3**		☐	☐	☐	☐
	**Outcome 4**		☐	☐	☐	☐
	**Outcome 5**		☐	☐	☐	☐
	**Outcome 6**		☐	☐	☐	☐
	**Outcome 7**		☐	☐	☐	☐

6	**Were the outcomes of participants included in any comparisons measured in the same way?**		**Yes**	**No**	**Unclear**	**N/A**
	**Outcome 1**	Fasting levels of insulin and glucose at baseline measured by a blood test at day 1 of the study for all study participants	☒	☐	☐	☐
	**Outcome 2**		☐	☐	☐	☐
	**Outcome 3**		☐	☐	☐	☐
	**Outcome 4**		☐	☐	☐	☐
	**Outcome 5**		☐	☐	☐	☐
	**Outcome 6**		☐	☐	☐	☐
	**Outcome 7**		☐	☐	☐	☐

7	**Were outcomes measured in a reliable way?**		**Yes**	**No**	**Unclear**	**N/A**

**Outcome 1**	Fasting levels of insulin and glucose at baseline measured by a blood test at day 1 of the study for all study participants	☒	☐	☐	☐
**Outcome 2**		☐	☐	☐	☐
**Outcome 3**		☐	☐	☐	☐
**Outcome 4**		☐	☐	☐	☐
**Outcome 5**		☐	☐	☐	☐
**Outcome 6**		☐	☐	☐	☐
**Outcome 7**		☐	☐	☐	☐

Bias related to participant retention

8	**Was follow-up complete and if not, were differences between groups in terms of their follow-up adequately described and analyzed?**	Follow up not relevant for outcome 1				
	**Outcome 1**		**Yes**	**No**	**Unclear**	**N/A**
	Result 1		☐	☐	☐	☐
	Result 2		☐	☐	☐	☐
	Result 3		☐	☐	☐	☐
	**Outcome 2**		**Yes**	**No**	**Unclear**	**N/A**
	Result 1		☐	☐	☐	☐
	Result 2		☐	☐	☐	☐
	Result 3		☐	☐	☐	☐

Outcome 3		Yes	No	Unclear	N/A
Result 1		☐	☐	☐	☐
Result 2		☐	☐	☐	☐
Result 3		☐	☐	☐	☐
**Outcome 4**		**Yes**	**No**	**Unclear**	**N/A**
Result 1		☐	☐	☐	☐
Result 2		☐	☐	☐	☐
Result 3		☐	☐	☐	☐
**Outcome 5**		**Yes**	**No**	**Unclear**	**N/A**
Result 1		☐	☐	☐	☐
Result 2		☐	☐	☐	☐
Result 3		☐	☐	☐	☐
**Outcome 6**		**Yes**	**No**	**Unclear**	**N/A**
Result 1		☐	☐	☐	☐
Result 2		☐	☐	☐	☐
Result 3		☐	☐	☐	☐
**Outcome 7**		**Yes**	**No**	**Unclear**	**N/A**
Result 1		☐	☐	☐	☐
Result 2		☐	☐	☐	☐
Result 3		☐	☐	☐	☐

Statistical Conclusion Validity

9	Was appropriate statistical analysis used?	Statical analysis not relevant for outcome 1				
	**Outcome 1**		**Yes**	**No**	**Unclear**	**N/A**
	Result 1		☐	☐	☐	☐
	Result 2		☐	☐	☐	☐
	Result 3		☐	☐	☐	☐
	**Outcome 2**		**Yes**	**No**	**Unclear**	**N/A**
	Result 1		☐	☐	☐	☐
	Result 2		☐	☐	☐	☐
	Result 3		☐	☐	☐	☐
	**Outcome 3**		**Yes**	**No**	**Unclear**	**N/A**
	Result 1		☐	☐	☐	☐
	Result 2		☐	☐	☐	☐
	Result 3		☐	☐	☐	☐
	**Outcome 4**		**Yes**	**No**	**Unclear**	**N/A**
	Result 1		☐	☐	☐	☐
	Result 2		☐	☐	☐	☐
	Result 3		☐	☐	☐	☐
	**Outcome 5**		**Yes**	**No**	**Unclear**	**N/A**

Result 1		☐	☐	☐	☐
Result 2		☐	☐	☐	☐
Result 3		☐	☐	☒	☐
**Outcome 6**		**Yes**	**No**	**Unclear**	**N/A**
Result 1		☐	☐	☐	☐
Result 2		☐	☐	☐	☐
Result 3		☐	☐	☐	☐
**Outcome 7**		**Yes**	**No**	**Unclear**	**N/A**
Result 1		☐	☐	☐	☐
Result 2		☐	☐	☐	☐
Result 3		☐	☐	☐	☐

Overall appraisal: **Include:** ☐ **Exclude:** ☐ **Seek Further Info:** ☐

Comments:

For our review, we only extract data about fasting insulin and glucose levels at baseline for patients with weight loss and patients with no weight loss, and this study is appraised according to measurement of these parameters.

S15 JBI checklist

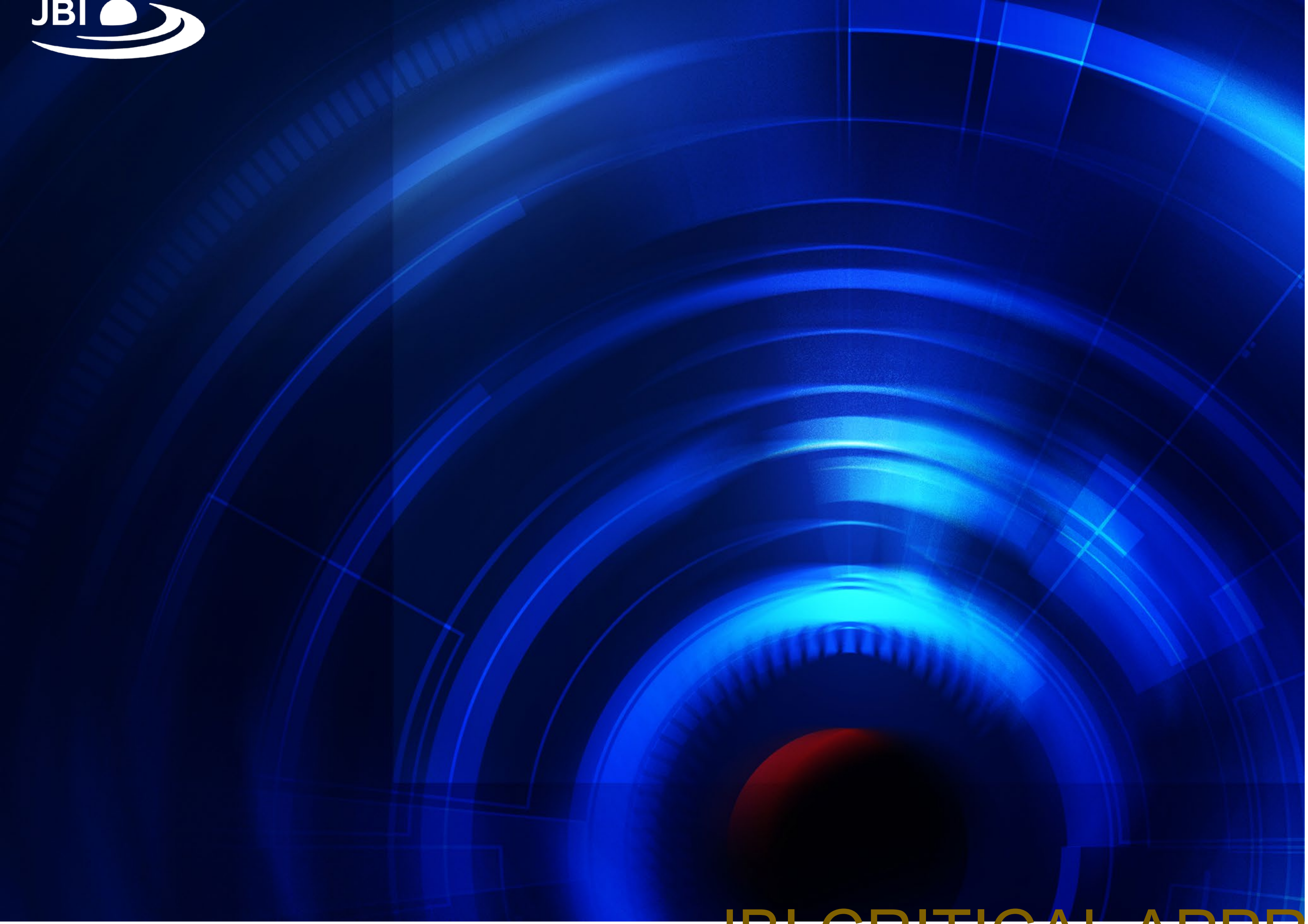

JBI CRITICAL APPRAISAL TOOL

JBI CHECKLIST FOR QUASI-EXPERIMENTAL STUDIES

2023

jbi.global

CRICOS Provider Number 00123M

INTRODUCTION

JBI is a global organization promoting and supporting evidence-based decisions that improve health and health service delivery. JBI offers a unique range of solutions to access, appraise and apply the best available evidence, servicing over 90 countries. Working with 80+ universities, hospitals and NGOs from across the globe through the JBI Collaboration, JBI is a recognized global leader in evidence-based health care.

JBI Systematic Reviews

The core of evidence synthesis is the systematic review of literature of a particular intervention, condition or issue. The systematic review is essentially an analysis of the available evidence and a judgment of the effectiveness or otherwise of a practice, involving a series of complex steps. JBI takes a particular view on what counts as evidence and the methods utilized to synthesize those different types of evidence. In line with this broader view of evidence, JBI has developed theories, methodologies and rigorous processes for the critical appraisal and synthesis of these diverse forms of evidence in order to aid in clinical decision-making in health care. Guidance now exists for conducting reviews of effectiveness research, qualitative research, prevalence/incidence, etiology/risk, economic evaluations, text/opinion, diagnostic test accuracy, mixed-methods, umbrella reviews and scoping reviews. Further information regarding JBI systematic reviews can be found in the JBI Manual for Evidence Synthesis.

JBI Critical Appraisal Tools

All systematic reviews incorporate a process of critique or appraisal of the research evidence. The purpose of this appraisal for quantitative evidence is to determine the extent to which a study has addressed the possibility of bias in its design, conduct and analysis. All papers selected for inclusion in the systematic review (that is – those that meet the inclusion criteria described in the protocol) need to be subjected to rigorous appraisal by two critical appraisers. The results of this appraisal can then be used to inform synthesis and interpretation of the results of the study. Although designed for use in systematic reviews, JBI critical appraisal tools can also be used when creating Critically Appraised Topics, in journal clubs and as an educational tool.

How were these tools developed?

JBI critical appraisal tools have been developed by JBI and collaborators. The particular iteration of this tool was developed by the JBI Effectiveness Methodology Group following oversight by the JBI Scientific Committee.

Like the previous versions of these tools, this version presents signaling questions to prompt reviewers to identify whether certain safeguards of bias have been met, in the primary literature under review. However, unlike previous iterations of this tool, this version has separated questions into whether they provide an answer relating to internal, external or statistical conclusion validity. For questions related to internal validity, these have been further separated to identify what domain of bias they are referring. Finally, this tool has also been structured to facilitate judgments related to bias at different levels (e.g. bias at the outcome level or bias at the result level) where appropriate.

These tools have been approved following extensive peer review by the JBI Scientific Committee.

How to cite

Please use the following when citing this tool: Barker TH, Habibi N, Aromataris E, Stone JC, Leonardi-Bee J, Sears K, et al. The revised JBI critical appraisal tool for the assessment of risk of bias quasi-experimental studies. JBI Evid Synth. 2024;22(3):378-88.

RoB Assessor: A.H. and J.S.	Date of Appraisal: 14.03.25	Record Number: PMID: 1542855
Study Author: Pisters P et al.	Study Title: Insulin action on glucose and branched-chain amino acid metabolism in cancer cachexia: differential effects of insulin	Study Year: 1992

Internal Validity		Choice - Comments/Justification	Yes	No	Unclear	N/A
**Bias related to temporal precedence**						
1	Is it clear in the study what is the "cause" and what is the "effect" (i.e. there is no confusion about which variable comes first)?		☒	☐	☐	☐
**Bias related to selection and allocation**						
2	Was there a control group?		☒	☐	☐	☐
**Bias related to confounding factors**						
3	Were participants included in any comparisons similar?	Yes, weight-losing patients with localized gastrointestinal cancer	☒	☐	☐	☐
**Bias related to administration of intervention/exposure**						
4	Were the participants included in any comparisons receiving similar treatment/care, other than the exposure or intervention of interest?	The study was conducted before the patients received treatment	☐	☒	☐	☐

Bias related to assessment, detection and measurement of the outcome						
5	Were there multiple measurements of the outcome, both pre and post the intervention/exposure?		Yes	No	Unclear	N/A

	**Outcome 1**	Fasting levels of insulin and glucose at baseline measured by a blood test at day 1 of the study for all study participants	☐	☒	☐	☐
	**Outcome 2**		☐	☐	☐	☐
	**Outcome 3**		☐	☐	☐	☐
	**Outcome 4**		☐	☐	☐	☐
	**Outcome 5**		☐	☐	☐	☐
	**Outcome 6**		☐	☐	☐	☐
	**Outcome 7**		☐	☐	☐	☐

6	**Were the outcomes of participants included in any comparisons measured in the same way?**		**Yes**	**No**	**Unclear**	**N/A**
	**Outcome 1**	Fasting levels of insulin and glucose at baseline measured by a blood test at day 1 of the study for all study participants	☒	☐	☐	☐
	**Outcome 2**		☐	☐	☐	☐
	**Outcome 3**		☐	☐	☐	☐
	**Outcome 4**		☐	☐	☐	☐
	**Outcome 5**		☐	☐	☐	☐
	**Outcome 6**		☐	☐	☐	☐
	**Outcome 7**		☐	☐	☐	☐

7	Were outcomes measured in a reliable way?		Yes	No	Unclear	N/A
	**Outcome 1**	Fasting levels of insulin and glucose at baseline measured by a blood test at day 1 of the study for all study participants	☒	☐	☐	☐
	**Outcome 2**		☐	☐	☐	☐
	**Outcome 3**		☐	☐	☐	☐
	**Outcome 4**		☐	☐	☐	☐
	**Outcome 5**		☐	☐	☐	☐
	**Outcome 6**		☐	☐	☐	☐
	**Outcome 7**		☐	☐	☐	☐

Bias related to participant retention

8	Was follow-up complete and if not, were differences between groups in terms of their follow-up adequately described and analyzed?	Follow up not relevant for outcome 1				
	**Outcome 1**		**Yes**	**No**	**Unclear**	**N/A**
	Result 1		☐	☐	☐	☐
	Result 2		☐	☐	☐	☐
	Result 3		☐	☐	☐	☐
	**Outcome 2**		**Yes**	**No**	**Unclear**	**N/A**
	Result 1		☐	☐	☐	☐
	Result 2		☐	☐	☐	☐

Result 3		☐	☐	☐	☐
**Outcome 3**		**Yes**	**No**	**Unclear**	**N/A**
Result 1		☐	☐	☐	☐
Result 2		☐	☐	☐	☐
Result 3		☐	☐	☐	☐
**Outcome 4**		**Yes**	**No**	**Unclear**	**N/A**
Result 1		☐	☐	☐	☐
Result 2		☐	☐	☐	☐
Result 3		☐	☐	☐	☐
**Outcome 5**		**Yes**	**No**	**Unclear**	**N/A**
Result 1		☐	☐	☐	☐
Result 2		☐	☐	☐	☐
Result 3		☐	☐	☐	☐
**Outcome 6**		**Yes**	**No**	**Unclear**	**N/A**
Result 1		☐	☐	☐	☐
Result 2		☐	☐	☐	☐
Result 3		☐	☐	☐	☐
**Outcome 7**		**Yes**	**No**	**Unclear**	**N/A**
Result 1		☐	☐	☐	☐
Result 2		☐	☐	☐	☐

Result 3		☐	☐	☐	☐

Statistical Conclusion Validity

9	**Was appropriate statistical analysis used?**	Statical analysis not relevant for outcome 1				
	**Outcome 1**		**Yes**	**No**	**Unclear**	**N/A**
	Result 1		☐	☐	☐	☐
	Result 2		☐	☐	☐	☐
	Result 3		☐	☐	☐	☐
	**Outcome 2**		**Yes**	**No**	**Unclear**	**N/A**
	Result 1		☐	☐	☐	☐
	Result 2		☐	☐	☐	☐
	Result 3		☐	☐	☐	☐
	**Outcome 3**		**Yes**	**No**	**Unclear**	**N/A**
	Result 1		☐	☐	☐	☐
	Result 2		☐	☐	☐	☐
	Result 3		☐	☐	☐	☐
	**Outcome 4**		**Yes**	**No**	**Unclear**	**N/A**
	Result 1		☐	☐	☐	☐
	Result 2		☐	☐	☐	☐
	Result 3		☐	☐	☐	☐

**Outcome 5**		**Yes**	**No**	**Unclear**	**N/A**
Result 1		☐	☐	☐	☐
Result 2		☐	☐	☐	☐
Result 3		☐	☐	☐	☐
**Outcome 6**		**Yes**	**No**	**Unclear**	**N/A**
Result 1		☐	☐	☐	☐
Result 2		☐	☐	☐	☐
Result 3		☐	☐	☐	☐
**Outcome 7**		**Yes**	**No**	**Unclear**	**N/A**
Result 1		☐	☐	☐	☐
Result 2		☐	☐	☐	☐
Result 3		☐	☐	☐	☐

Overall appraisal: **Include:** ☐ **Exclude:** ☐ **Seek Further Info:** ☐

Comments:

For our review, we only extract data about fasting insulin and glucose levels at baseline for patients, and this study is appraised according to measurement of these parameters.

S16 JBI checklist

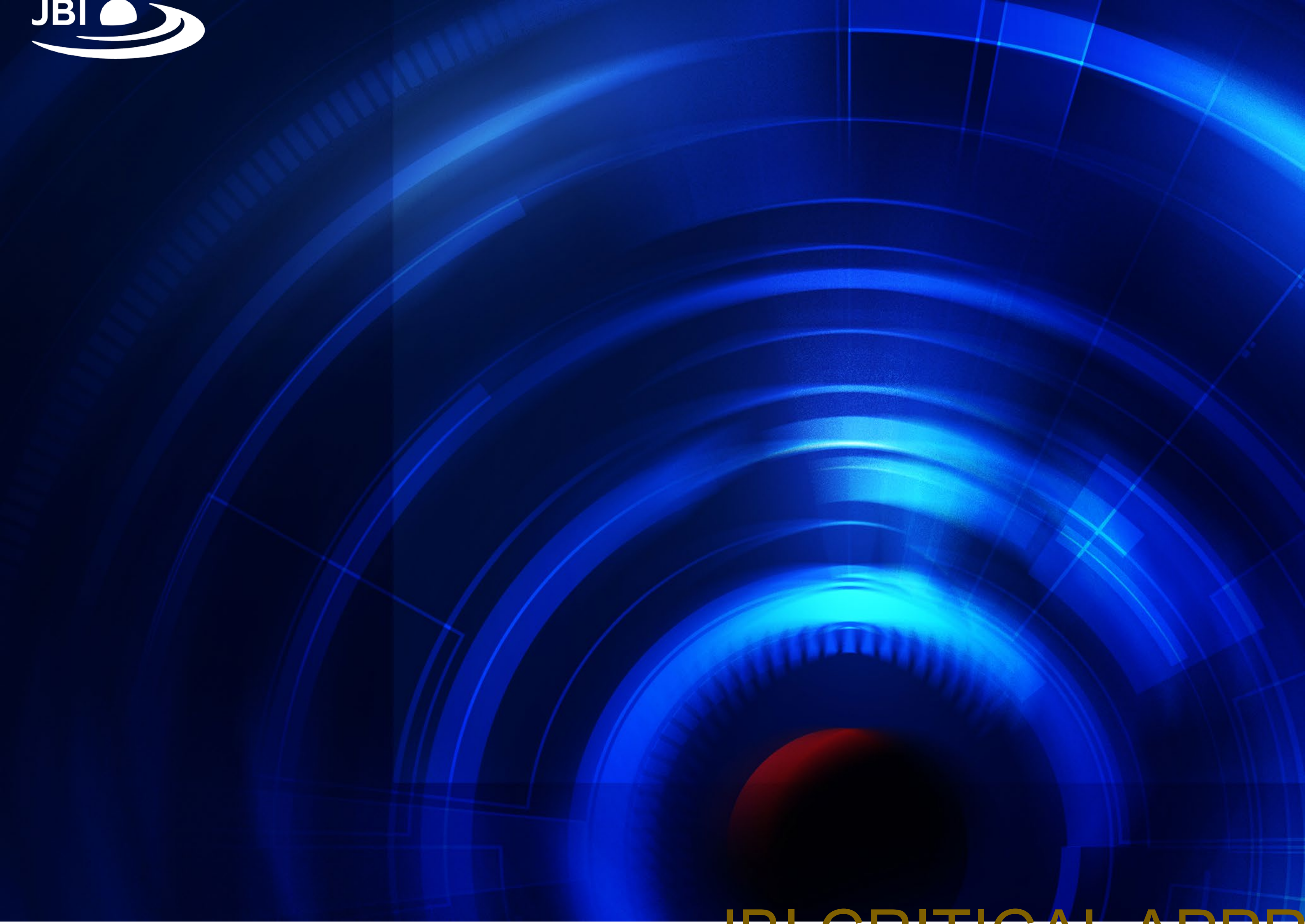

JBI CRITICAL APPRAISAL TOOL

JBI CHECKLIST FOR QUASI-EXPERIMENTAL STUDIES

2023

jbi.global

CRICOS Provider Number 00123M

INTRODUCTION

JBI is a global organization promoting and supporting evidence-based decisions that improve health and health service delivery. JBI offers a unique range of solutions to access, appraise and apply the best available evidence, servicing over 90 countries. Working with 80+ universities, hospitals and NGOs from across the globe through the JBI Collaboration, JBI is a recognized global leader in evidence-based health care.

JBI Systematic Reviews

The core of evidence synthesis is the systematic review of literature of a particular intervention, condition or issue. The systematic review is essentially an analysis of the available evidence and a judgment of the effectiveness or otherwise of a practice, involving a series of complex steps. JBI takes a particular view on what counts as evidence and the methods utilized to synthesize those different types of evidence. In line with this broader view of evidence, JBI has developed theories, methodologies and rigorous processes for the critical appraisal and synthesis of these diverse forms of evidence in order to aid in clinical decision-making in health care. Guidance now exists for conducting reviews of effectiveness research, qualitative research, prevalence/incidence, etiology/risk, economic evaluations, text/opinion, diagnostic test accuracy, mixed-methods, umbrella reviews and scoping reviews. Further information regarding JBI systematic reviews can be found in the JBI Manual for Evidence Synthesis.

JBI Critical Appraisal Tools

All systematic reviews incorporate a process of critique or appraisal of the research evidence. The purpose of this appraisal for quantitative evidence is to determine the extent to which a study has addressed the possibility of bias in its design, conduct and analysis. All papers selected for inclusion in the systematic review (that is – those that meet the inclusion criteria described in the protocol) need to be subjected to rigorous appraisal by two critical appraisers. The results of this appraisal can then be used to inform synthesis and interpretation of the results of the study. Although designed for use in systematic reviews, JBI critical appraisal tools can also be used when creating Critically Appraised Topics, in journal clubs and as an educational tool.

How were these tools developed?

JBI critical appraisal tools have been developed by JBI and collaborators. The particular iteration of this tool was developed by the JBI Effectiveness Methodology Group following oversight by the JBI Scientific Committee.

Like the previous versions of these tools, this version presents signaling questions to prompt reviewers to identify whether certain safeguards of bias have been met, in the primary literature under review. However, unlike previous iterations of this tool, this version has separated questions into whether they provide an answer relating to internal, external or statistical conclusion validity. For questions related to internal validity, these have been further separated to identify what domain of bias they are referring. Finally, this tool has also been structured to facilitate judgments related to bias at different levels (e.g. bias at the outcome level or bias at the result level) where appropriate.

These tools have been approved following extensive peer review by the JBI Scientific Committee.

How to cite

Please use the following when citing this tool: Barker TH, Habibi N, Aromataris E, Stone JC, Leonardi-Bee J, Sears K, et al. The revised JBI critical appraisal tool for the assessment of risk of bias quasi-experimental studies. JBI Evid Synth. 2024;22(3):378-88.

**RoB Assessor: A.H. and J.S.**	**Date of Appraisal: 14.03.25**	**Record Number: PMID: 8010722**
**Study Author: Rofe A et al**	**Study Title: Altered insulin response to glucose i weight-losing cancer patients**	**Study Year: 1994**

Internal Validity		Choice - Comments/Justification	Yes	No	Unclear	N/A
**Bias related to temporal precedence**						
1	**Is it clear in the study what is the "cause" and what is the "effect" (i.e. there is no confusion about which variable comes first)?**		☒	☐	☐	☐
**Bias related to selection and allocation**						
2	**Was there a control group?**		☒	☐	☐	☐
**Bias related to confounding factors**						
3	**Were participants included in any comparisons similar?**	Yes, weight-losing patients with advanced cancer	☒	☐	☐	☐
**Bias related to administration of intervention/exposure**						
4	**Were the participants included in any comparisons receiving similar treatment/care, other than the exposure or intervention of interest?**	Patients were previously treated, but did not receive treatment during the study	☐	☒	☐	☐

Bias related to assessment, detection and measurement of the outcome			Yes	No	Unclear	N/A
5	**Were there multiple measurements of the outcome, both pre and post the intervention/exposure?**					

**Outcome 1**	Fasting levels of insulin and glucose at baseline measured by a blood test at day 1 of the study for all study participants	☐	☒	☐	☐
**Outcome 2**		☐	☐	☐	☐
**Outcome 3**		☐	☐	☐	☐
**Outcome 4**		☐	☐	☐	☐
**Outcome 5**		☐	☐	☐	☐
**Outcome 6**		☐	☐	☐	☐
**Outcome 7**		☐	☐	☐	☐

6	**Were the outcomes of participants included in any comparisons measured in the same way?**		**Yes**	**No**	**Unclear**	**N/A**
	**Outcome 1**	Fasting levels of insulin and glucose at baseline measured by a blood test at day 1 of the study for all study participants	☒	☐	☐	☐
	**Outcome 2**		☐	☐	☐	☐
	**Outcome 3**		☐	☐	☐	☐
	**Outcome 4**		☐	☐	☐	☐
	**Outcome 5**		☐	☐	☐	☐
	**Outcome 6**		☐	☐	☐	☐
	**Outcome 7**		☐	☐	☐	☐

7	Were outcomes measured in a reliable way?		Yes	No	Unclear	N/A
	**Outcome 1**	Fasting levels of insulin and glucose at baseline measured by a blood test at day 1 of the study for all study participants	☒	☐	☐	☐
	**Outcome 2**		☐	☐	☐	☐
	**Outcome 3**		☐	☐	☐	☐
	**Outcome 4**		☐	☐	☐	☐
	**Outcome 5**		☐	☐	☐	☐
	**Outcome 6**		☐	☐	☐	☐
	**Outcome 7**		☐	☐	☐	☐

Bias related to participant retention

8	Was follow-up complete and if not, were differences between groups in terms of their follow-up adequately described and analyzed?	Follow up not relevant for outcome 1				
	**Outcome 1**		**Yes**	**No**	**Unclear**	**N/A**
	Result 1		☐	☐	☐	☐
	Result 2		☐	☐	☐	☐
	Result 3		☐	☐	☐	☐
	**Outcome 2**		**Yes**	**No**	**Unclear**	**N/A**
	Result 1		☐	☐	☐	☐
	Result 2		☐	☐	☐	☐

		Yes	No	Unclear	N/A
Result 3		☐	☐	☐	☐
**Outcome 3**		**Yes**	**No**	**Unclear**	**N/A**
Result 1		☐	☐	☐	☐
Result 2		☐	☐	☐	☐
Result 3		☐	☐	☐	☐
**Outcome 4**		**Yes**	**No**	**Unclear**	**N/A**
Result 1		☐	☐	☐	☐
Result 2		☐	☐	☐	☐
Result 3		☐	☐	☐	☐
**Outcome 5**		**Yes**	**No**	**Unclear**	**N/A**
Result 1		☐	☐	☐	☐
Result 2		☐	☐	☐	☐
Result 3		☐	☐	☐	☐
**Outcome 6**		**Yes**	**No**	**Unclear**	**N/A**
Result 1		☐	☐	☐	☐
Result 2		☐	☐	☐	☐
Result 3		☐	☐	☐	☐
**Outcome 7**		**Yes**	**No**	**Unclear**	**N/A**
Result 1		☐	☐	☐	☐

Result 2		☐	☐	☐	☐
Result 3		☐	☐	☐	☐

Statistical Conclusion Validity

9	**Was appropriate statistical analysis used?**	Statical analysis not relevant for outcome 1				
	**Outcome 1**		**Yes**	**No**	**Unclear**	**N/A**
	Result 1		☐	☐	☐	☐
	Result 2		☐	☐	☐	☐
	Result 3		☐	☐	☐	☐
	**Outcome 2**		**Yes**	**No**	**Unclear**	**N/A**
	Result 1		☐	☐	☐	☐
	Result 2		☐	☐	☐	☐
	Result 3		☐	☐	☐	☐
	**Outcome 3**		**Yes**	**No**	**Unclear**	**N/A**
	Result 1		☐	☐	☐	☐
	Result 2		☐	☐	☐	☐
	Result 3		☐	☐	☐	☐
	**Outcome 4**		**Yes**	**No**	**Unclear**	**N/A**
	Result 1		☐	☐	☐	☐

Result 2		☐	☐	☐	☐
Result 3		☐	☐	☐	☐
**Outcome 5**		**Yes**	**No**	**Unclear**	**N/A**
Result 1		☐	☐	☐	☐
Result 2		☐	☐	☐	☐
Result 3		☐	☐	☐	☐
**Outcome 6**		**Yes**	**No**	**Unclear**	**N/A**
Result 1		☐	☐	☐	☐
Result 2		☐	☐	☐	☐
Result 3		☐	☐	☐	☐
**Outcome 7**		**Yes**	**No**	**Unclear**	**N/A**
Result 1		☐	☐	☐	☐
Result 2		☐	☐	☐	☐
Result 3		☐	☐	☐	☐

Overall appraisal: **Include:** ☐ **Exclude:** ☐ **Seek Further Info:** ☐

Comments:

For our review, we only extract data about fasting insulin and glucose levels at baseline for patients, and this study is appraised according to measurement of these parameters.

© JBI, 2022. All rights reserved. JBI grants use of these tools for research purposes only. All other enquiries should be sent to jbisynthesis@adelaide.edu.au

S17 JBI checklist

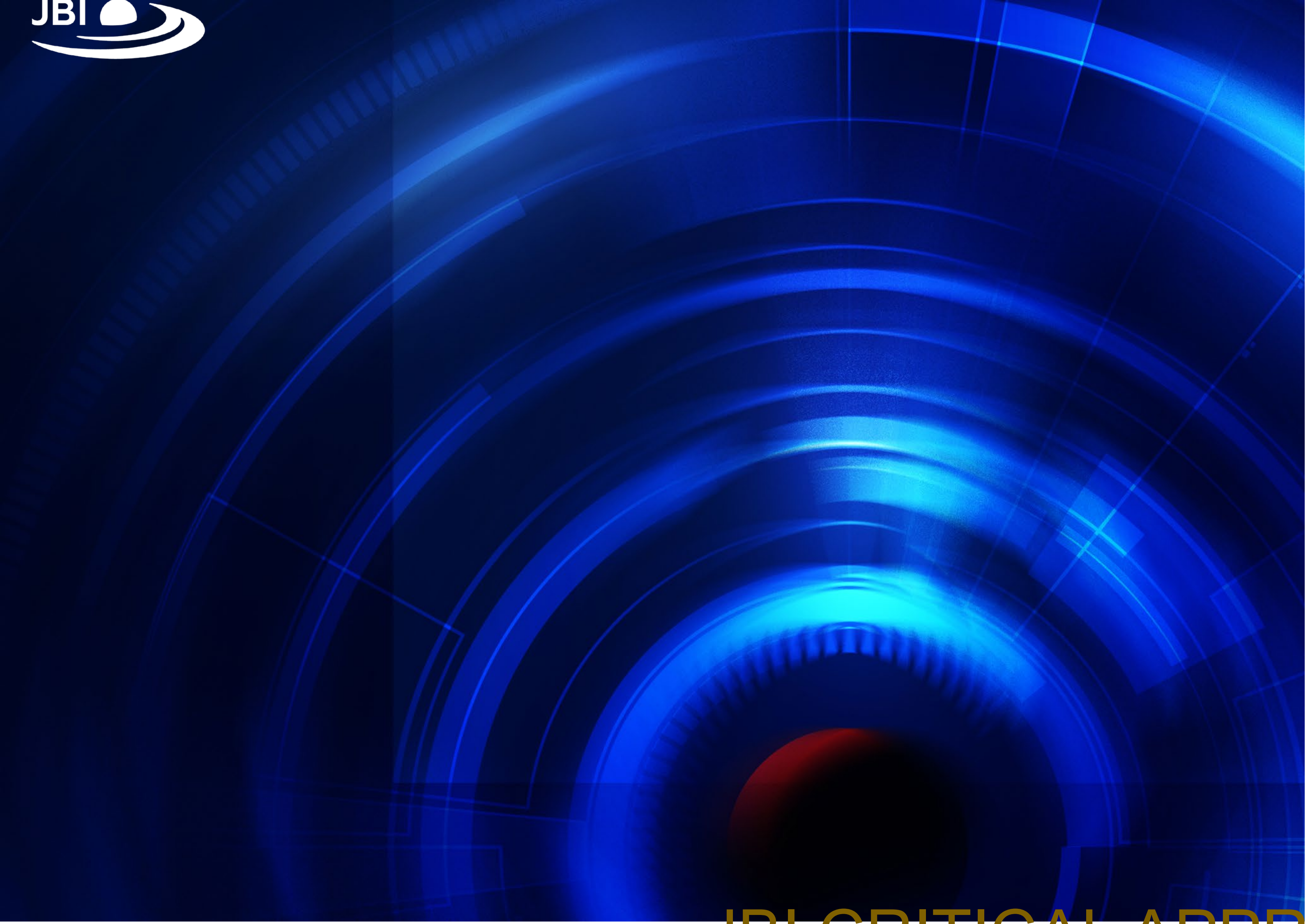

JBI CRITICAL APPRAISAL TOOL

JBI CHECKLIST FOR QUASI-EXPERIMENTAL STUDIES

2023

jbi.global

CRICOS Provider Number 00123M

INTRODUCTION

JBI is a global organization promoting and supporting evidence-based decisions that improve health and health service delivery. JBI offers a unique range of solutions to access, appraise and apply the best available evidence, servicing over 90 countries. Working with 80+ universities, hospitals and NGOs from across the globe through the JBI Collaboration, JBI is a recognized global leader in evidence-based health care.

JBI Systematic Reviews

The core of evidence synthesis is the systematic review of literature of a particular intervention, condition or issue. The systematic review is essentially an analysis of the available evidence and a judgment of the effectiveness or otherwise of a practice, involving a series of complex steps. JBI takes a particular view on what counts as evidence and the methods utilized to synthesize those different types of evidence. In line with this broader view of evidence, JBI has developed theories, methodologies and rigorous processes for the critical appraisal and synthesis of these diverse forms of evidence in order to aid in clinical decision-making in health care. Guidance now exists for conducting reviews of effectiveness research, qualitative research, prevalence/incidence, etiology/risk, economic evaluations, text/opinion, diagnostic test accuracy, mixed-methods, umbrella reviews and scoping reviews. Further information regarding JBI systematic reviews can be found in the JBI Manual for Evidence Synthesis.

JBI Critical Appraisal Tools

All systematic reviews incorporate a process of critique or appraisal of the research evidence. The purpose of this appraisal for quantitative evidence is to determine the extent to which a study has addressed the possibility of bias in its design, conduct and analysis. All papers selected for inclusion in the systematic review (that is – those that meet the inclusion criteria described in the protocol) need to be subjected to rigorous appraisal by two critical appraisers. The results of this appraisal can then be used to inform synthesis and interpretation of the results of the study. Although designed for use in systematic reviews, JBI critical appraisal tools can also be used when creating Critically Appraised Topics, in journal clubs and as an educational tool.

How were these tools developed?

JBI critical appraisal tools have been developed by JBI and collaborators. The particular iteration of this tool was developed by the JBI Effectiveness Methodology Group following oversight by the JBI Scientific Committee.

Like the previous versions of these tools, this version presents signaling questions to prompt reviewers to identify whether certain safeguards of bias have been met, in the primary literature under review. However, unlike previous iterations of this tool, this version has separated questions into whether they provide an answer relating to internal, external or statistical conclusion validity. For questions related to internal validity, these have been further separated to identify what domain of bias they are referring. Finally, this tool has also been structured to facilitate judgments related to bias at different levels (e.g. bias at the outcome level or bias at the result level) where appropriate.

These tools have been approved following extensive peer review by the JBI Scientific Committee.

How to cite

Please use the following when citing this tool: Barker TH, Habibi N, Aromataris E, Stone JC, Leonardi-Bee J, Sears K, et al. The revised JBI critical appraisal tool for the assessment of risk of bias quasi-experimental studies. JBI Evid Synth. 2024;22(3):378-88.

**RoB Assessor: A.H. and J.S.**	**Date of Appraisal: 14.03.25**	**Record Number: PMID: 8023270**
**Study Author: Yoshikawa T et al.**	**Study Title: Effects of tumor removal and body weight loss on insulin resistance in patients with cancer**	**Study Year: 1994**

Internal Validity		Choice - Comments/Justification	Yes	No	Unclear	N/A
**Bias related to temporal precedence**						
1	**Is it clear in the study what is the "cause" and what is the "effect" (i.e. there is no confusion about which variable comes first)?**		☒	☐	☐	☐
**Bias related to selection and allocation**						
2	**Was there a control group?**		☒	☐	☐	☐
**Bias related to confounding factors**						
3	**Were participants included in any comparisons similar?**	Yes, cancer patients with weight-loss, and cancer patients without weight-loss	☒	☐	☐	☐
**Bias related to administration of intervention/exposure**						
4	**Were the participants included in any comparisons receiving similar treatment/care, other than the exposure or intervention of interest?**	It is unclear if the patients receive treatment during the study	☐	☐	☒	☐

**Bias related to assessment, detection and measurement of the outcome**						
5	**Were there multiple measurements of the outcome, both pre and post the intervention/exposure?**		**Yes**	**No**	**Unclear**	**N/A**

	**Outcome 1**	Fasting levels of insulin and glucose at baseline measured by a blood test at day 1 of the study for all study participants	☐	☒	☐	☐
	**Outcome 2**		☐	☐	☐	☐
	**Outcome 3**		☐	☐	☐	☐
	**Outcome 4**		☐	☐	☐	☐
	**Outcome 5**		☐	☐	☐	☐
	**Outcome 6**		☐	☐	☐	☐
	**Outcome 7**		☐	☐	☐	☐

6	**Were the outcomes of participants included in any comparisons measured in the same way?**		**Yes**	**No**	**Unclear**	**N/A**
	**Outcome 1**	Fasting levels of insulin and glucose at baseline measured by a blood test at day 1 of the study for all study participants	☒	☐	☐	☐
	**Outcome 2**		☐	☐	☐	☐
	**Outcome 3**		☐	☐	☐	☐
	**Outcome 4**		☐	☐	☐	☐
	**Outcome 5**		☐	☐	☐	☐
	**Outcome 6**		☐	☐	☐	☐
	**Outcome 7**		☐	☐	☐	☐

7	Were outcomes measured in a reliable way?		Yes	No	Unclear	N/A
	**Outcome 1**	Fasting levels of insulin and glucose at baseline measured by a blood test at day 1 of the study for all study participants	☒	☐	☐	☐
	**Outcome 2**		☐	☐	☐	☐
	**Outcome 3**		☐	☐	☐	☐
	**Outcome 4**		☐	☐	☐	☐
	**Outcome 5**		☐	☐	☐	☐
	**Outcome 6**		☐	☐	☐	☐
	**Outcome 7**		☐	☐	☐	☐

Bias related to participant retention

8	Was follow-up complete and if not, were differences between groups in terms of their follow-up adequately described and analyzed?	Follow up not relevant for outcome 1				
	**Outcome 1**		**Yes**	**No**	**Unclear**	**N/A**
	Result 1		☐	☐	☐	☐
	Result 2		☐	☐	☐	☐
	Result 3		☐	☐	☐	☐
	**Outcome 2**		**Yes**	**No**	**Unclear**	**N/A**
	Result 1		☐	☐	☐	☐
	Result 2		☐	☐	☐	☐

Result 3		☐	☐	☐	☐
**Outcome 3**		**Yes**	**No**	**Unclear**	**N/A**
Result 1		☐	☐	☐	☐
Result 2		☐	☐	☐	☐
Result 3		☐	☐	☐	☐
**Outcome 4**		**Yes**	**No**	**Unclear**	**N/A**
Result 1		☐	☐	☐	☐
Result 2		☐	☐	☐	☐
Result 3		☐	☐	☐	☐
**Outcome 5**		**Yes**	**No**	**Unclear**	**N/A**
Result 1		☐	☐	☐	☐
Result 2		☐	☐	☐	☐
Result 3		☐	☐	☐	☐
**Outcome 6**		**Yes**	**No**	**Unclear**	**N/A**
Result 1		☐	☐	☐	☐
Result 2		☐	☐	☐	☐
Result 3		☐	☐	☐	☐
**Outcome 7**		**Yes**	**No**	**Unclear**	**N/A**
Result 1		☐	☐	☐	☐
Result 2		☐	☐	☐	☐

Result 3		☐	☐	☐	☐

Statistical Conclusion Validity

9	Was appropriate statistical analysis used?	Statical analysis not relevant for outcome 1				
	**Outcome 1**		**Yes**	**No**	**Unclear**	**N/A**
	Result 1		☐	☐	☐	☐
	Result 2		☐	☐	☐	☐
	Result 3		☐	☐	☐	☐
	**Outcome 2**		**Yes**	**No**	**Unclear**	**N/A**
	Result 1		☐	☐	☐	☐
	Result 2		☐	☐	☐	☐
	Result 3		☐	☐	☐	☐
	**Outcome 3**		**Yes**	**No**	**Unclear**	**N/A**
	Result 1		☐	☐	☐	☐
	Result 2		☐	☐	☐	☐
	Result 3		☐	☐	☐	☐
	**Outcome 4**		**Yes**	**No**	**Unclear**	**N/A**
	Result 1		☐	☐	☐	☐
	Result 2		☐	☐	☐	☐
	Result 3		☐	☐	☐	☐

**Outcome 5**		**Yes**	**No**	**Unclear**	**N/A**
Result 1		☐	☐	☐	☐
Result 2		☐	☐	☐	☐
Result 3		☐	☐	☐	☐
**Outcome 6**		**Yes**	**No**	**Unclear**	**N/A**
Result 1		☐	☐	☐	☐
Result 2		☐	☐	☐	☐
Result 3		☐	☐	☐	☐
**Outcome 7**		**Yes**	**No**	**Unclear**	**N/A**
Result 1		☐	☐	☐	☐
Result 2		☐	☐	☐	☐
Result 3		☐	☐	☐	☐

Overall appraisal: **Include:** ☐ **Exclude:** ☐ **Seek Further Info:** ☐

Comments:

For our review, we only extract data about fasting insulin and glucose levels at baseline for patients with weight loss and patients with no weight loss, and this study is appraised according to measurement of these parameters.

S18 JBI checklist

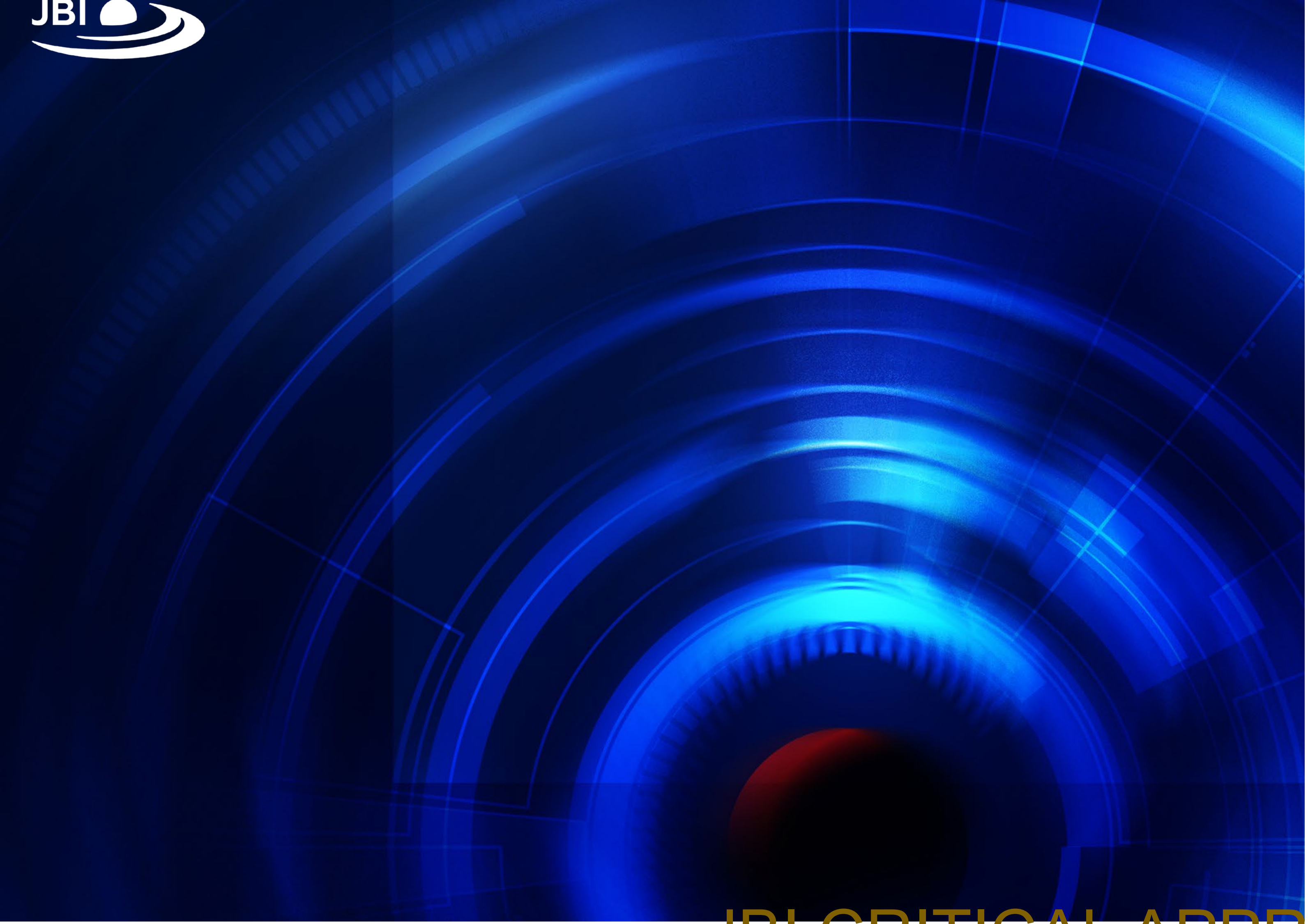

JBI CRITICAL APPRAISAL TOOL

JBI CHECKLIST FOR QUASI-EXPERIMENTAL STUDIES

2023

jbi.global

CRICOS Provider Number 00123M

INTRODUCTION

JBI is a global organization promoting and supporting evidence-based decisions that improve health and health service delivery. JBI offers a unique range of solutions to access, appraise and apply the best available evidence, servicing over 90 countries. Working with 80+ universities, hospitals and NGOs from across the globe through the JBI Collaboration, JBI is a recognized global leader in evidence-based health care.

JBI Systematic Reviews

The core of evidence synthesis is the systematic review of literature of a particular intervention, condition or issue. The systematic review is essentially an analysis of the available evidence and a judgment of the effectiveness or otherwise of a practice, involving a series of complex steps. JBI takes a particular view on what counts as evidence and the methods utilized to synthesize those different types of evidence. In line with this broader view of evidence, JBI has developed theories, methodologies and rigorous processes for the critical appraisal and synthesis of these diverse forms of evidence in order to aid in clinical decision-making in health care. Guidance now exists for conducting reviews of effectiveness research, qualitative research, prevalence/incidence, etiology/risk, economic evaluations, text/opinion, diagnostic test accuracy, mixed-methods, umbrella reviews and scoping reviews. Further information regarding JBI systematic reviews can be found in the JBI Manual for Evidence Synthesis.

JBI Critical Appraisal Tools

All systematic reviews incorporate a process of critique or appraisal of the research evidence. The purpose of this appraisal for quantitative evidence is to determine the extent to which a study has addressed the possibility of bias in its design, conduct and analysis. All papers selected for inclusion in the systematic review (that is – those that meet the inclusion criteria described in the protocol) need to be subjected to rigorous appraisal by two critical appraisers. The results of this appraisal can then be used to inform synthesis and interpretation of the results of the study. Although designed for use in systematic reviews, JBI critical appraisal tools can also be used when creating Critically Appraised Topics, in journal clubs and as an educational tool.

How were these tools developed?

JBI critical appraisal tools have been developed by JBI and collaborators. The particular iteration of this tool was developed by the JBI Effectiveness Methodology Group following oversight by the JBI Scientific Committee.

Like the previous versions of these tools, this version presents signaling questions to prompt reviewers to identify whether certain safeguards of bias have been met, in the primary literature under review. However, unlike previous iterations of this tool, this version has separated questions into whether they provide an answer relating to internal, external or statistical conclusion validity. For questions related to internal validity, these have been further separated to identify what domain of bias they are referring. Finally, this tool has also been structured to facilitate judgments related to bias at different levels (e.g. bias at the outcome level or bias at the result level) where appropriate.

These tools have been approved following extensive peer review by the JBI Scientific Committee.

How to cite

Please use the following when citing this tool: Barker TH, Habibi N, Aromataris E, Stone JC, Leonardi-Bee J, Sears K, et al. The revised JBI critical appraisal tool for the assessment of risk of bias quasi-experimental studies. JBI Evid Synth. 2024;22(3):378-88.

RoB Assessor: A.H. and J.S.	Date of Appraisal: 14.03.25	Record Number: 10.1016/0261-5614(90)90047-V
Study Author: Selberg O et al.	Study Title: Palmitate turnover and its response glucose infusion in weight-losing cancer patients	Study Year: 1990

Internal Validity		Choice - Comments/Justification	Yes	No	Unclear	N/A
**Bias related to temporal precedence**						
1	Is it clear in the study what is the "cause" and what is the "effect" (i.e. there is no confusion about which variable comes first)?		☒	☐	☐	☐
**Bias related to selection and allocation**						
2	Was there a control group?		☒	☐	☐	☐
**Bias related to confounding factors**						
3	Were participants included in any comparisons similar?	Yes, weight-losing cancer patients	☒	☐	☐	☐
**Bias related to administration of intervention/exposure**						
4	Were the participants included in any comparisons receiving similar treatment/care, other than the exposure or intervention of interest?	It is unclear if the patients receive treatment during the study	☐	☐	☒	☐

Bias related to assessment, detection and measurement of the outcome						
5	Were there multiple measurements of the outcome, both pre and post the intervention/exposure?		Yes	No	Unclear	N/A
	Outcome 1	Fasting levels of insulin and glucose at baseline measured by a blood test at day 1 of the study for all study participants	☐	☒	☐	☐

	Outcome 2		☐	☐	☐	☐
	Outcome 3		☐	☐	☐	☐
	Outcome 4		☐	☐	☐	☐
	Outcome 5		☐	☐	☐	☐
	Outcome 6		☐	☐	☐	☐
	Outcome 7		☐	☐	☐	☐

6	**Were the outcomes of participants included in any comparisons measured in the same way?**		**Yes**	**No**	**Unclear**	**N/A**
	**Outcome 1**	Fasting levels of insulin and glucose at baseline measured by a blood test at day 1 of the study for all study participants	☒	☐	☐	☐
	**Outcome 2**		☐	☐	☐	☐
	**Outcome 3**		☐	☐	☐	☐
	**Outcome 4**		☐	☐	☐	☐
	**Outcome 5**		☐	☐	☐	☐
	**Outcome 6**		☐	☐	☐	☐
	**Outcome 7**		☐	☐	☐	☐

7	**Were outcomes measured in a reliable way?**		**Yes**	**No**	**Unclear**	**N/A**

**Outcome 1**	Fasting levels of insulin and glucose at baseline measured by a blood test at day 1 of the study for all study participants	☒	☐	☐	☐
**Outcome 2**		☐	☐	☐	☐
**Outcome 3**		☐	☐	☐	☐
**Outcome 4**		☐	☐	☐	☐
**Outcome 5**		☐	☐	☐	☐
**Outcome 6**		☐	☐	☐	☐
**Outcome 7**		☐	☐	☐	☐

Bias related to participant retention

8	**Was follow-up complete and if not, were differences between groups in terms of their follow-up adequately described and analyzed?**	Follow up not relevant for outcome 1				
	**Outcome 1**		**Yes**	**No**	**Unclear**	**N/A**
	Result 1		☐	☐	☐	☐
	Result 2		☐	☐	☐	☐
	Result 3		☐	☐	☐	☐
	**Outcome 2**		**Yes**	**No**	**Unclear**	**N/A**
	Result 1		☐	☐	☐	☐
	Result 2		☐	☐	☐	☐
	Result 3		☐	☐	☐	☐

		Yes	No	Unclear	N/A
**Outcome 3**		**Yes**	**No**	**Unclear**	**N/A**
Result 1		☐	☐	☐	☐
Result 2		☐	☐	☐	☐
Result 3		☐	☐	☐	☐
**Outcome 4**		**Yes**	**No**	**Unclear**	**N/A**
Result 1		☐	☐	☐	☐
Result 2		☐	☐	☐	☐
Result 3		☐	☐	☐	☐
**Outcome 5**		**Yes**	**No**	**Unclear**	**N/A**
Result 1		☐	☐	☐	☐
Result 2		☐	☐	☐	☐
Result 3		☐	☐	☐	☐
**Outcome 6**		**Yes**	**No**	**Unclear**	**N/A**
Result 1		☐	☐	☐	☐
Result 2		☐	☐	☐	☐
Result 3		☐	☐	☐	☐
**Outcome 7**		**Yes**	**No**	**Unclear**	**N/A**
Result 1		☐	☐	☐	☐
Result 2		☐	☐	☐	☐
Result 3		☐	☐	☐	☐

Statistical Conclusion Validity

9	Was appropriate statistical analysis used?	Statical analysis not relevant for outcome 1				
	**Outcome 1**		**Yes**	**No**	**Unclear**	**N/A**
	Result 1		☐	☐	☐	☐
	Result 2		☐	☐	☐	☐
	Result 3		☐	☐	☐	☐
	**Outcome 2**		**Yes**	**No**	**Unclear**	**N/A**
	Result 1		☐	☐	☐	☐
	Result 2		☐	☐	☐	☐
	Result 3		☐	☐	☐	☐
	**Outcome 3**		**Yes**	**No**	**Unclear**	**N/A**
	Result 1		☐	☐	☐	☐
	Result 2		☐	☐	☐	☐
	Result 3		☐	☐	☐	☐
	**Outcome 4**		**Yes**	**No**	**Unclear**	**N/A**
	Result 1		☐	☐	☐	☐
	Result 2		☐	☐	☐	☐
	Result 3		☐	☐	☐	☐
	**Outcome 5**		**Yes**	**No**	**Unclear**	**N/A**

Result 1		☐	☐	☐	☐
Result 2		☐	☐	☐	☐
Result 3		☐	☐	☐	☐
**Outcome 6**		**Yes**	**No**	**Unclear**	**N/A**
Result 1		☐	☐	☐	☐
Result 2		☐	☐	☐	☐
Result 3		☐	☐	☐	☐
**Outcome 7**		**Yes**	**No**	**Unclear**	**N/A**
Result 1		☐	☐	☐	☐
Result 2		☐	☐	☐	☐
Result 3		☐	☐	☐	☐

Overall appraisal: **Include:** ☐ **Exclude:** ☐ **Seek Further Info:** ☐

Comments:

For our review, we only extract data about fasting insulin and glucose levels at baseline for patients, and this study is appraised according to measurement of these parameters.

S19 JBI checklist

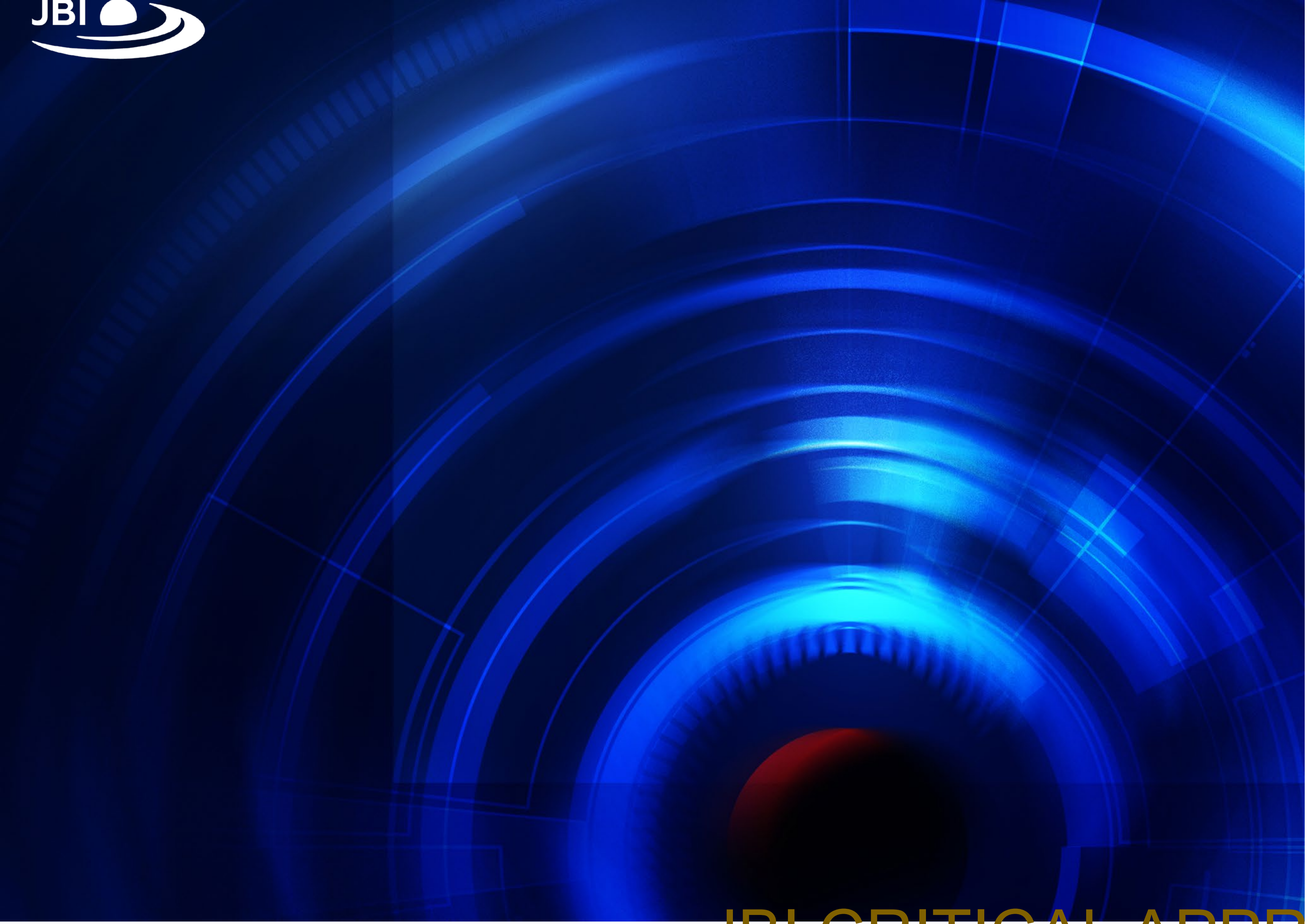

JBI CRITICAL APPRAISAL TOOL

JBI CHECKLIST FOR QUASI-EXPERIMENTAL STUDIES

2023

jbi.global

CRICOS Provider Number 00123M

INTRODUCTION

JBI is a global organization promoting and supporting evidence-based decisions that improve health and health service delivery. JBI offers a unique range of solutions to access, appraise and apply the best available evidence, servicing over 90 countries. Working with 80+ universities, hospitals and NGOs from across the globe through the JBI Collaboration, JBI is a recognized global leader in evidence-based health care.

JBI Systematic Reviews

The core of evidence synthesis is the systematic review of literature of a particular intervention, condition or issue. The systematic review is essentially an analysis of the available evidence and a judgment of the effectiveness or otherwise of a practice, involving a series of complex steps. JBI takes a particular view on what counts as evidence and the methods utilized to synthesize those different types of evidence. In line with this broader view of evidence, JBI has developed theories, methodologies and rigorous processes for the critical appraisal and synthesis of these diverse forms of evidence in order to aid in clinical decision-making in health care. Guidance now exists for conducting reviews of effectiveness research, qualitative research, prevalence/incidence, etiology/risk, economic evaluations, text/opinion, diagnostic test accuracy, mixed-methods, umbrella reviews and scoping reviews. Further information regarding JBI systematic reviews can be found in the JBI Manual for Evidence Synthesis.

JBI Critical Appraisal Tools

All systematic reviews incorporate a process of critique or appraisal of the research evidence. The purpose of this appraisal for quantitative evidence is to determine the extent to which a study has addressed the possibility of bias in its design, conduct and analysis. All papers selected for inclusion in the systematic review (that is – those that meet the inclusion criteria described in the protocol) need to be subjected to rigorous appraisal by two critical appraisers. The results of this appraisal can then be used to inform synthesis and interpretation of the results of the study. Although designed for use in systematic reviews, JBI critical appraisal tools can also be used when creating Critically Appraised Topics, in journal clubs and as an educational tool.

How were these tools developed?

JBI critical appraisal tools have been developed by JBI and collaborators. The particular iteration of this tool was developed by the JBI Effectiveness Methodology Group following oversight by the JBI Scientific Committee.

Like the previous versions of these tools, this version presents signaling questions to prompt reviewers to identify whether certain safeguards of bias have been met, in the primary literature under review. However, unlike previous iterations of this tool, this version has separated questions into whether they provide an answer relating to internal, external or statistical conclusion validity. For questions related to internal validity, these have been further separated to identify what domain of bias they are referring. Finally, this tool has also been structured to facilitate judgments related to bias at different levels (e.g. bias at the outcome level or bias at the result level) where appropriate.

These tools have been approved following extensive peer review by the JBI Scientific Committee.

How to cite

Please use the following when citing this tool: Barker TH, Habibi N, Aromataris E, Stone JC, Leonardi-Bee J, Sears K, et al. The revised JBI critical appraisal tool for the assessment of risk of bias quasi-experimental studies. JBI Evid Synth. 2024;22(3):378-88.

**RoB Assessor: A.H and J.S.**	**Date of Appraisal: 14.03.25**	**Record Number: oi:10.1158/0008-5472.CAN-06-4585**
**Study Author: Agustsson T et al.**	**Study Title: Mechanism of Increased Lipolysis in Cancer Cachexia**	**Study Year: 2007**

	Internal Validity	Choice - Comments/Justification	Yes	No	Unclear	N/A
	**Bias related to temporal precedence**					
1	**Is it clear in the study what is the "cause" and what is the "effect" (i.e. there is no confusion about which variable comes first)?**		☒	☐	☐	☐
	**Bias related to selection and allocation**					
2	**Was there a control group?**		☒	☐	☐	☐
	**Bias related to confounding factors**					
3	**Were participants included in any comparisons similar?**	Yes, cancer patients with weight-loss, and cancer patients without weight-loss	☒	☐	☐	☐
	**Bias related to administration of intervention/exposure**					
4	**Were the participants included in any comparisons receiving similar treatment/care, other than the exposure or intervention of interest?**	Patients had not received any prior treatment.	☐	☒	☐	☐

	Bias related to assessment, detection and measurement of the outcome		Yes	No	Unclear	N/A
5	**Were there multiple measurements of the outcome, both pre and post the intervention/exposure?**					
	**Outcome 1**	Fasting levels of insulin and glucose at baseline measured by a blood test at day 1 of the study for all study participants	☐	☒	☐	☐

	**Outcome 2**		☐	☐	☐	☐
	**Outcome 3**		☐	☐	☐	☐
	**Outcome 4**		☐	☐	☐	☐
	**Outcome 5**		☐	☐	☐	☐
	**Outcome 6**		☐	☐	☐	☐
	**Outcome 7**		☐	☐	☐	☐

6	**Were the outcomes of participants included in any comparisons measured in the same way?**		**Yes**	**No**	**Unclear**	**N/A**
	**Outcome 1**	Fasting levels of insulin and glucose at baseline measured by a blood test at day 1 of the study for all study participants	☒	☐	☐	☐
	**Outcome 2**		☐	☐	☐	☐
	**Outcome 3**		☐	☐	☐	☐
	**Outcome 4**		☐	☐	☐	☐
	**Outcome 5**		☐	☐	☐	☐
	**Outcome 6**		☐	☐	☐	☐
	**Outcome 7**		☐	☐	☐	☐

7	**Were outcomes measured in a reliable way?**		**Yes**	**No**	**Unclear**	**N/A**

**Outcome 1**	Fasting levels of insulin and glucose at baseline measured by a blood test at day 1 of the study for all study participants	☒	☐	☐	☐
**Outcome 2**		☐	☐	☐	☐
**Outcome 3**		☐	☐	☐	☐
**Outcome 4**		☐	☐	☐	☐
**Outcome 5**		☐	☐	☐	☐
**Outcome 6**		☐	☐	☐	☐
**Outcome 7**		☐	☐	☐	☐

Bias related to participant retention

8	**Was follow-up complete and if not, were differences between groups in terms of their follow-up adequately described and analyzed?**	Follow up not relevant for outcome 1				
	**Outcome 1**		**Yes**	**No**	**Unclear**	**N/A**
	Result 1		☐	☐	☐	☐
	Result 2		☐	☐	☐	☐
	Result 3		☐	☐	☐	☐
	**Outcome 2**		**Yes**	**No**	**Unclear**	**N/A**
	Result 1		☐	☐	☐	☐
	Result 2		☐	☐	☐	☐
	Result 3		☐	☐	☐	☐

Outcome 3		Yes	No	Unclear	N/A
Result 1		☐	☐	☐	☐
Result 2		☐	☐	☐	☐
Result 3		☐	☐	☐	☐
**Outcome 4**		**Yes**	**No**	**Unclear**	**N/A**
Result 1		☐	☐	☐	☐
Result 2		☐	☐	☐	☐
Result 3		☐	☐	☐	☐
**Outcome 5**		**Yes**	**No**	**Unclear**	**N/A**
Result 1		☐	☐	☐	☐
Result 2		☐	☐	☐	☐
Result 3		☐	☐	☐	☐
**Outcome 6**		**Yes**	**No**	**Unclear**	**N/A**
Result 1		☐	☐	☐	☐
Result 2		☐	☐	☐	☐
Result 3		☐	☐	☐	☐
**Outcome 7**		**Yes**	**No**	**Unclear**	**N/A**
Result 1		☐	☐	☐	☐
Result 2		☐	☐	☐	☐
Result 3		☐	☐	☐	☐

Statistical Conclusion Validity

9	Was appropriate statistical analysis used?	Statical analysis not relevant for outcome 1				
	**Outcome 1**		**Yes**	**No**	**Unclear**	**N/A**
	Result 1		☐	☐	☐	☐
	Result 2		☐	☐	☐	☐
	Result 3		☐	☐	☐	☐
	**Outcome 2**		**Yes**	**No**	**Unclear**	**N/A**
	Result 1		☐	☐	☐	☐
	Result 2		☐	☐	☐	☐
	Result 3		☐	☐	☐	☐
	**Outcome 3**		**Yes**	**No**	**Unclear**	**N/A**
	Result 1		☐	☐	☐	☐
	Result 2		☐	☐	☐	☐
	Result 3		☐	☐	☐	☐
	**Outcome 4**		**Yes**	**No**	**Unclear**	**N/A**
	Result 1		☐	☐	☐	☐
	Result 2		☐	☐	☐	☐
	Result 3		☐	☐	☐	☐
	**Outcome 5**		**Yes**	**No**	**Unclear**	**N/A**

Result 1		☐	☐	☐	☐
Result 2		☐	☐	☐	☐
Result 3		☐	☐	☐	☐
**Outcome 6**		**Yes**	**No**	**Unclear**	**N/A**
Result 1		☐	☐	☐	☐
Result 2		☐	☐	☐	☐
Result 3		☐	☐	☐	☐
**Outcome 7**		**Yes**	**No**	**Unclear**	**N/A**
Result 1		☐	☐	☐	☐
Result 2		☐	☐	☐	☐
Result 3		☐	☐	☐	☐

Overall appraisal: **Include:** ☐ **Exclude:** ☐ **Seek Further Info:** ☐

Comments:

For our review, we only extract data about fasting insulin and glucose levels at baseline for patients with weight loss and patients with no weight loss, and this study is appraised according to measurement of these parameters.

Table S20: Excluded reports after full-text screening

Report	Reason for exclusion
Allen S, Brown V, White D, King D, Hunt J, Prabhu P, Rockall T, Preston S, Sultan J. Multi-modal prehabilitation during neoadjuvant therapy prior to resection for oesophagogastric cancer: a pilot randomised controlled trial. Br J Surg. 2019;106:90-1.	No fasting glucose/insulin data
Barber MD, Fearon KC, Tisdale MJ, McMillan DC, Ross JA. Effect of a fish oil-enriched nutritional supplement on metabolic mediators in patients with pancreatic cancer cachexia. Nutr Cancer. 2001;40(2):118-24.	No fasting glucose/insulin data
Barber MD, McMillan DC, Wallace AM, Ross JA, Preston T, Fearon KC. The response of leptin, interleukin-6 and fat oxidation to feeding in weight-losing patients with pancreatic cancer. Br J Cancer. 2004 Mar 22;90(6):1129-32.	Other reason (uses subgroup from other study)
Begenik H, Aslan M, Dulger AC, Emre H, Kemik A, Kemik O, Esen R. Serum leptin levels in gastric cancer patients and the relationship with insulin resistance. Arch Med Sci. 2015 Apr 25;11(2):346-52.	No weight loss data
Bennegård K, Lindmark L, Edén E, Svaninger G, Lundholm K. Flux of amino acids across the leg in weight-losing cancer patients. Cancer Res. 1984 Jan;44(1):386-93.	Other reason (uses subgroup from other study)
Byerley LO, Heber D, Bergman RN, Dubria M, Chi J. Insulin action and metabolism in patients with head and neck cancer. Cancer. 1991 Jun 1;67(11):2900-6.	No weight loss data
Caeiro L, Anderson LJ, Liu H, Krumm K, Garcia JM. THU512 Elevated insulin like growth factor binding protein 2 levels are associated with muscle wasting and increased insulin sensitivity in cancer patients. J Endocr Soc. 2023 Nov 1;7(Suppl 1):bvad114.2140.	No weight loss data
Chlebowski RT, Heber D, Richardson B, Block JB. Influence of hydrazine sulfate on abnormal carbohydrate metabolism in cancer patients with weight loss. Cancer Res. 1984 Feb;44(2):857-61.	No fasting glucose/insulin data

Copeland GP, Al-Sumidaie AM, Leinster SJ, Davis JC, Hipkin LH. Glucose metabolism in patients with gastrointestinal malignancy but without excessive weight loss. Eur J Surg Oncol. 1987 Feb;13(1):11-6.	No fasting glucose/insulin data
Copeland GP, Leinster SJ, Davis JC, Hipkin LH. Postoperative glucose metabolism in patients with gastrointestinal malignancy. Eur J Surg Oncol. 1988 Dec;14(6):677-83.	Not cancer
Dawson JK, Dorff TB, Todd Schroeder E, Lane CJ, Gross ME, Dieli-Conwright CM. Impact of resistance training on body composition and metabolic syndrome variables during androgen deprivation therapy for prostate cancer: a pilot randomized controlled trial. BMC Cancer. 2018 Apr 3;18(1):368.	No weight loss data
de Carvalho TM, Miguel Marin D, da Silva CA, de Souza AL, Talamoni M, Lima CS, Monte Alegre S. Evaluation of patients with head and neck cancer performing standard treatment in relation to body composition, resting metabolic rate, and inflammatory cytokines. Head Neck. 2015 Jan;37(1):97-102.	No weight loss data
Dong J, Zeng Y, Zhang P, Li C, Chen Y, Li Y, Wang K. Serum IGFBP2 Level Is a New Candidate Biomarker of Severe Malnutrition in Advanced Lung Cancer. Nutr Cancer. 2020;72(5):858-63.	No weight loss data
Dülger H, Alici S, Sekeroğlu MR, Erkog R, Ozbek H, Noyan T, Yavuz M. Serum levels of leptin and proinflammatory cytokines in patients with gastrointestinal cancer. Int J Clin Pract. 2004 Jun;58(6):545-9.	No weight loss data
Fouladiun M, Körner U, Bosaeus I, Daneryd P, Hyltander A, Lundholm KG. Body composition and time course changes in regional distribution of fat and lean tissue in unselected cancer patients on palliative care--correlations with food intake, metabolism, exercise capacity, and hormones. Cancer. 2005 May 15;103(10):2189-98.	No fasting glucose/insulin data
Gambardella A, Paolisso G, D'Amore A, Granato M, Verza M, Varricchio M. Different contribution of substrates oxidation to insulin resistance in malnourished elderly patients with cancer. Cancer. 1993 Nov 15;72(10):3106-13.	No fasting glucose/insulin data
Garcia JM, Garcia-Touza M, Hijazi RA, Taffet G, Epner D, Mann D, Smith RG, Cunningham GR, Marcelli M. Active ghrelin levels and active to total ghrelin ratio in cancer-induced cachexia. J Clin Endocrinol Metab. 2005 May;90(5):2920-6.	Other reason (includes patients with diabetes)

Iwai N, Sakai H, Oka K, Sakagami J, Okuda T, Hattori C, Taniguchi M, Hara T, Tsuji T, Komaki T, Kagawa K, Doi T, Ishikawa T, Yasuda H, Itoh Y. Predictors of response to anamorelin in gastrointestinal cancer patients with cachexia: a retrospective study. Support Care Cancer. 2023 Jan 14;31(2):115.	No weight loss data
Kerem M, Ferahkose Z, Yilmaz UT, Pasaoglu H, Ofluoglu E, Bedirli A, Salman B, Sahin TT, Akin M. Adipokines and ghrelin in gastric cancer cachexia. World J Gastroenterol. 2008 Jun 21;14(23):3633-41.	Other reason (includes patients with diabetes)
Kokal WA, McCulloch A, Wright PD, Johnston ID. Glucose turnover and recycling in colorectal carcinoma. Ann Surg. 1983 Nov;198(5):601-4.	No fasting glucose/insulin data
Körber J, Pricelius S, Heidrich M, Müller MJ. Increased lipid utilization in weight losing and weight stable cancer patients with normal body weight. Eur J Clin Nutr. 1999 Sep;53(9):740-5.	No fasting glucose/insulin data
Li R, Ma ML, Song YY, Pei J, Zhong RB, Qian JL, Yan B, Zhang XY, Shen J, Han BH. Investigation on the nutrition status of 132 advanced lung cancer patients in primary treatment. Tumor. 2008;28(4):353-6.	No weight loss data
Liao WC, Chen PR, Huang CC, Chang YT, Huang BS, Chang CC, Wu MS, Chow LP. Relationship between pancreatic cancer-associated diabetes and cachexia. J Cachexia Sarcopenia Muscle. 2020 Aug;11(4):899-908.	No fasting glucose/insulin data
Lyu H, Yang X, Ding R, Cui H, Qiao J, Zhu M, Wei J. A prospective observational study on nutritional status of patients with pancreatic tumor. Chin J Clin Nutr. 2017;25(2):94-8.	No weight loss data
Makino T, Noguchi Y, Yoshikawa T, Doi C, Nomura K. Circulating interleukin 6 concentrations and insulin resistance in patients with cancer. Br J Surg. 1998 Dec;85(12):1658-62.	No fasting glucose/insulin data
Mendes MCS, Juliani FL, Branbilla SR, Souza AL, Alegre SM, Costa FO, Martinez CAR, Carvalheira JBC. Body composition and insulin sensitivity in patients with rectal cancer. Hematol Transfus Cell Ther. 2024;46(Suppl 2):S5-6.	No weight loss data

Newman E, Heslin MJ, Wolf RF, Pisters PW, Brennan MF. The effect of insulin on glucose and protein metabolism in the forearm of cancer patients. Surg Oncol. 1992 Aug;1(4):257-67.	No fasting glucose/insulin data
Rantaniemi L, Siltari A, Harju E, Murtola TJ. Can supervised exercise impact on metabolic markers and physical activity during androgen-deprivation therapy in prostate cancer patients? - randomized controlled pilot trial. Cancer Res. 2022;82(Suppl 12):CT171.	No weight loss data
Ruan GT, Deng L, Xie HL, Shi JY, Liu XY, Zheng X, Chen Y, Lin SQ, Zhang HY, Liu CA, Ge YZ, Song MM, Hu CL, Zhang XW, Yang M, Hu W, Cong MH, Zhu LC, Wang KH, Shi HP. Systemic inflammation and insulin resistance-related indicator predicts poor outcome in patients with cancer cachexia. Cancer Metab. 2024 Jan 25;12(1):3.	No fasting glucose/insulin data
Shaw JH, Humberstone DA, Holdaway C. Weight loss in patients with head and neck cancer: malnutrition or tumour effect? Aust N Z J Surg. 1988 Jun;58(6):505-9.	No weight loss data
Smiechowska J, Utech A, Taffet G, Hayes T, Marcelli M, Garcia JM. Adipokines in patients with cancer anorexia and cachexia. J Investig Med. 2010 Mar;58(3):554-9.	Other reason (uses subgroup from other study)
Tayek JA, Bulcavage L, Chlebowski RT. Relationship of hepatic glucose production to growth hormone and severity of malnutrition in a population with colorectal carcinoma. Cancer Res. 1990 Apr 1;50(7):2119-22.	No weight loss data
Tayek JA, Chlebowski RT. Metabolic response to chemotherapy in colon cancer patients. JPEN J Parenter Enteral Nutr. 1992 Nov-Dec;16(6 Suppl):65S-71S.	No weight loss data
Tayek JA, Manglik S, Abemayor E. Insulin secretion, glucose production, and insulin sensitivity in underweight and normal-weight volunteers, and in underweight and normal-weight cancer patients: a Clinical Research Center study. Metabolism. 1997 Feb;46(2):140-5.	No weight loss data
Tůma P, Hložek T, Kamišová J, Gojda J. Monitoring of circulating amino acids in patients with pancreatic cancer and cancer cachexia using capillary electrophoresis and contactless conductivity detection. Electrophoresis. 2021 Oct;42(19):1885-1891.	No weight loss data

Winter A, MacAdams J, Chevalier S. Normal protein anabolic response to hyperaminoacidemia in insulin-resistant patients with lung cancer cachexia. Clin Nutr. 2012 Oct;31(5):765-73.	No weight loss data
Yoshikawa T, Noguchi Y, Doi C, Makino T, Nomura K. Insulin resistance in patients with cancer: relationships with tumor site, tumor stage, body-weight loss, acute-phase response, and energy expenditure. Nutrition. 2001 Jul-Aug;17(7-8):590-3.	No weight loss data
Zwickl H, Hackner K, Köfeler H, Krzizek EC, Muqaku B, Pils D, Scharnagl H, Solheim TS, Zwickl-Traxler E, Pecherstorfer M. Reduced LDL-Cholesterol and Reduced Total Cholesterol as Potential Indicators of Early Cancer in Male Treatment-Naïve Cancer Patients With Pre-cachexia and Cachexia. Front Oncol. 2020 Aug 4;10:1262.	Wrong population
